# Supplementary material for: Rare coding variants in NOX4 link high ROS levels to psoriatic arthritis mutilans
Source: EMBO Mol Med. 2024 Feb 20;16(3):9. doi: 10.1038/s44321-024-00035-z (PMC10940640; doi:10.1038/s44321-024-00035-z)
Supplement: Supplementary file 1 — Appendix [file 44321_2024_35_MOESM1_ESM.pdf]

Appendix for:

“Rare coding variants in *NOX4* link high ROS levels to psoriatic arthritis mutilans”

Sailan Wang, Pernilla Nikamo *et al*.

Contents: Page

Contents:	Page
Appendix Figure S1:	2
Appendix Figure S2:	3
Appendix Table S1:	4
Appendix Table S2:	5
Appendix Table S3:	6
Appendix Table S4:	7
Appendix Table S5:	8
Appendix Table S6:	9
Appendix Table S7:	10

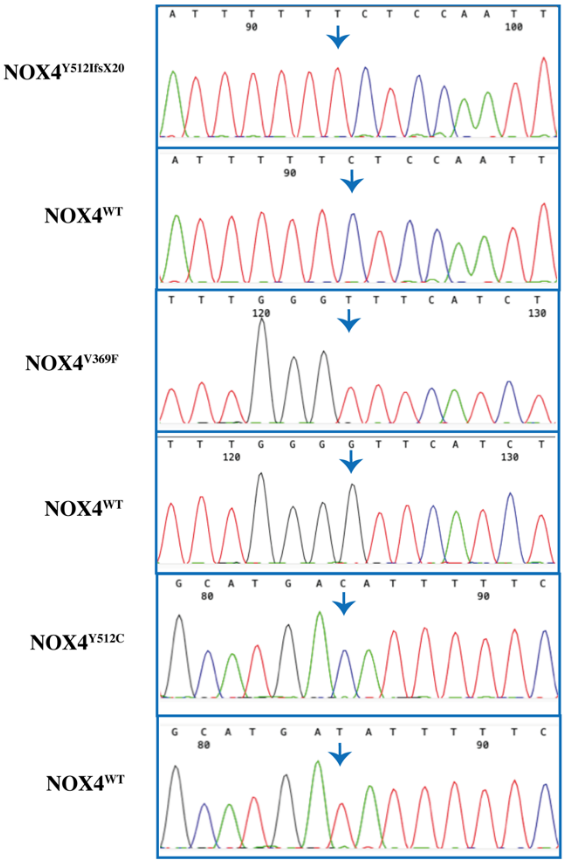

Appendix Figure S1. Sanger sequencing results from plasmid constructs.

Forward-sequencing result of the plasmid NOX4^{Y512IfsX20} showing an extra T nucleotide insertion, which caused a frameshift. Forward-sequencing result of the plasmid NOX4^{V369F} showing Phenylalanine (F) replaced by Valine (V) on protein. Forward-sequencing result of the plasmid NOX4^{Y512C} showing Cystine (C) replaced by Tyrosine (Y) on protein. The arrow indicates the vector/insert junction.

Data information: DNA bases are color-coded: A, green; G, black; C, blue; and T, red.

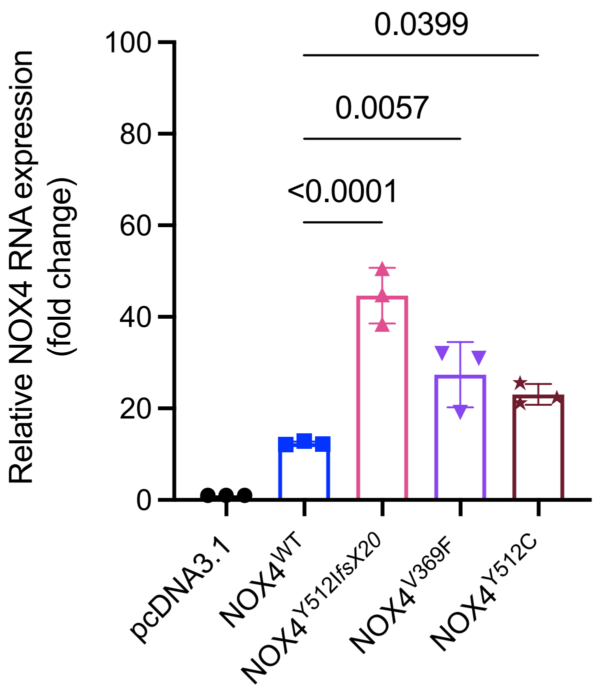

Appendix Figure S2. NOX4 mRNA expression in HEK293 stable cells.

Primers were designed between exons 1-3. Quantitative real-time PCR analysis of the NOX4 from wild-type or NOX4-variants expressing HEK293 cells. The quantification of stable cells with NOX4 mutants were up-regulated compared with WT. Relative levels were normalized to β-actin. *N*=3.

Data information: *N* = biological replicates. error bars in figure represent mean ± SD (one-way ANOVA).

Appendix Table S1. Genotyping of the *NOX4* variant- rs11018268 in PsO, PsA and control groups.

Alleles[1]	Controls (N=451)	PsO No PsA (N=562)	PsA (N=492)	PAM (N=63)
T/C	364/75/1	462/87/3	400/74/4	52/10/1

[1]Major/Minor

Appendix Table S2. Primers used for qPCR

Target gene	Primer sequence (5'-3')	
	Forward	Reverse
ACTB	CAACCGCGAGAAGATGAC	AGGAAGGCTGGAAGAGTG
NOX4_exon1_3	CTGTGTCCTGGAGGAGCTGG	AAGCCAAGAGTGTTCGGCAC
NOX4_exon15_18	CTTCCGTTGGTTTGCAGATT	TGGGTCCACAACAGAAAACA

Appendix Table S3. Primers and probes used for Genotyping.

SNP	Genotyping PCR primers	Genotyping probes
rs781430033	F: CGAGGACGTCCTATAAACAGTCTTG	VIC reporter: CATGATATTTTTTCTCC
	R: GTAGTGCTTTCTTTTTGGCAGAAGAT	FAM reporter: GCATGATATTTTTCTCC
rs144215891	F: CGAGGACGTCCTATAAACAGTCTTG	VIC reporter: AGTGCATGATATTTTTC
	R: GTAGTGCTTTCTTTTTGGCAGAAGAT	FAM reporter: TGCATGACATTTTTC
rs765662279	F: CTACATACCTGTCCAGTCTCCTACT	VIC reporter: AAGATGAACCCCAAATGT
	R: GTGTCCAACTGAAACCAAAGCA	FAM reporter: AAGATGAAACCCAAATGT

F: forward; R: reverse

Appendix Table S4. Primers used for genomic DNA Sanger sequencing.

SNP	Forward	Reverse
rs781430033/ rs144215891	CAAAAGTTTCCACCGAGGACG	ACAGCAATTTGGTGGGAAGCC
rs765662279	AATTATTACATTCCACTAT	GTTGTAATTGAATTATAATT

Appendix Table S5. Primers used for site-directed mutagenesis.

Plasmids	Mutagenesis primers
$NOX4^{Y512IfsX20}$	oligonucleotide primer #1: GAATTCAGTGCATGATATTTTTTCTCCAATTATCTTCTGTATCCCATCT
	oligonucleotide primer #2: AGATGGGATACAGAAGATAATTGGAGAAAAAATATCATGCACTGAATTC
$NOX4^{V369F}$	oligonucleotide primer #1: GTCTCCTACTATTTTAAGATGAAACCCAAATGTTGCTTTGGTTTCAG
	oligonucleotide primer #2: CTGAAACCAAAGCAACATTTGGTTTCATCTTAAAATAGTAGGAGAC
$NOX4^{Y512C}$	oligonucleotide primer #1: TGAATTCAGTGCATGACATTTTTCTCCAATTATCTTCTGTATCCCATC
	oligonucleotide primer #2: GATGGGATACAGAAGATAATTGGAGAAAAATGTCATGCACTGAATTCA

Appendix Table S6. Primers used for Sanger sequencing of plasmids

Target gene	Primer sequence (5'-3')	
	Forward	Reverse
$NOX4^{Y512IfsX20}$ / $NOX4^{Y512C}$	CTTCCGTTGGTTTGCAGATT	TGGGTCCACAACAGAAAACA
$NOX4^{V369F}$	TCCCTCAGATGTCATGGAAATC	TGAAGGGCAGAATTTCGGAG

Appendix Table S7. Number of zebrafish embryos imaged in each experimental group.

Number	NOX4^{WT}/ *nox4* atg MO	NOX4^{Y512fsX20}/ *nox4* atg MO	NOX4^{V369F}/ *nox4* atg MO	NOX4^{Y512C}/ *nox4* atg MO
First experiment	11	10	11	16
Second experiment	11	13	10	12
Third experiment	11	10	11	9
