# Supplementary figures and images for: Rare coding variants in NOX4 link high ROS levels to psoriatic arthritis mutilans
Source: EMBO Mol Med. 2024 Feb 20;16(3):9. doi: 10.1038/s44321-024-00035-z (PMC10940640; doi:10.1038/s44321-024-00035-z)

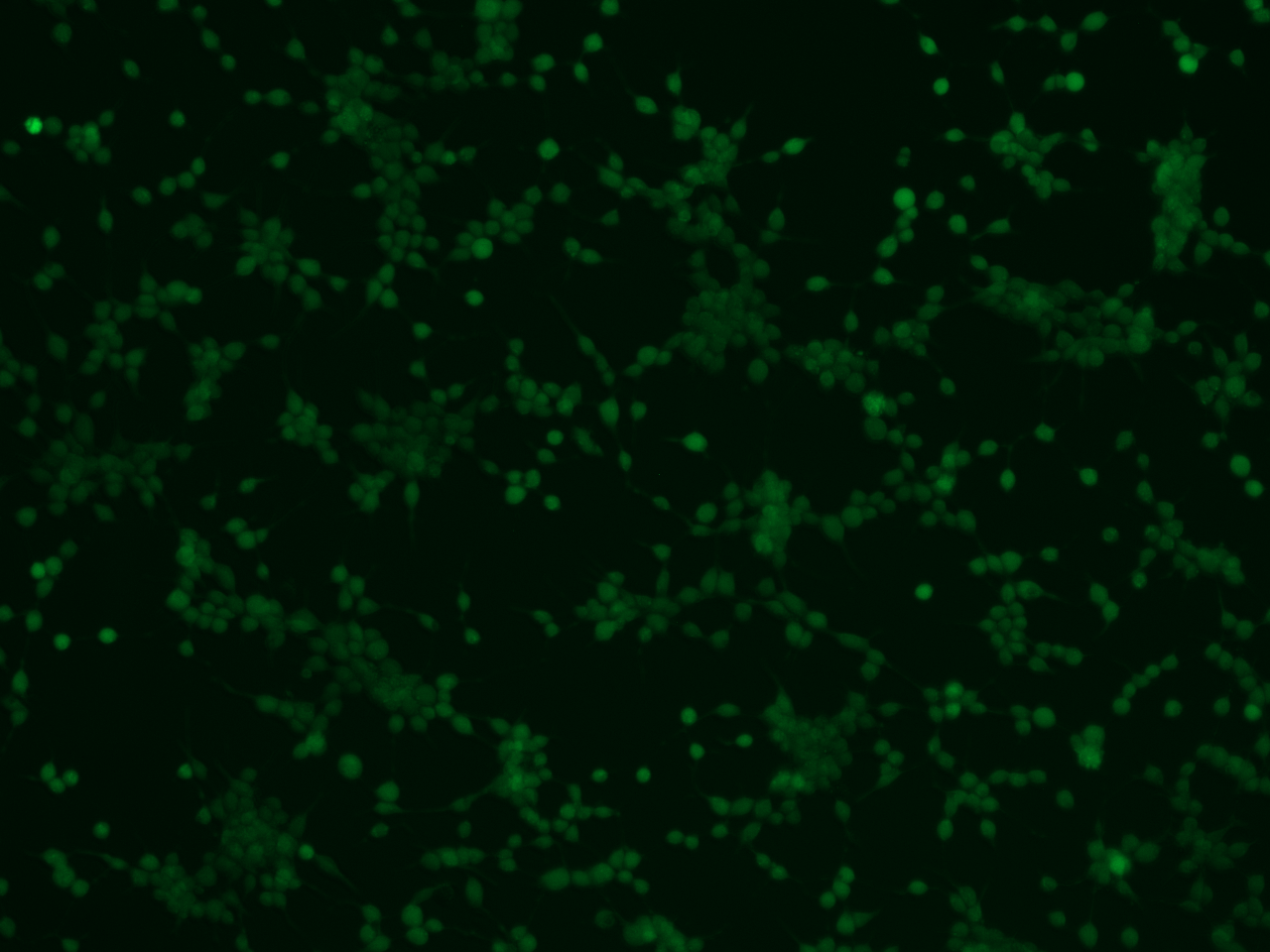

Supplement: Supplementary file 4 — Source Data Fig. 3 [file 44321_2024_35_MOESM4_ESM.zip › Figure 3/3D/4 NOX4V369F.tif]

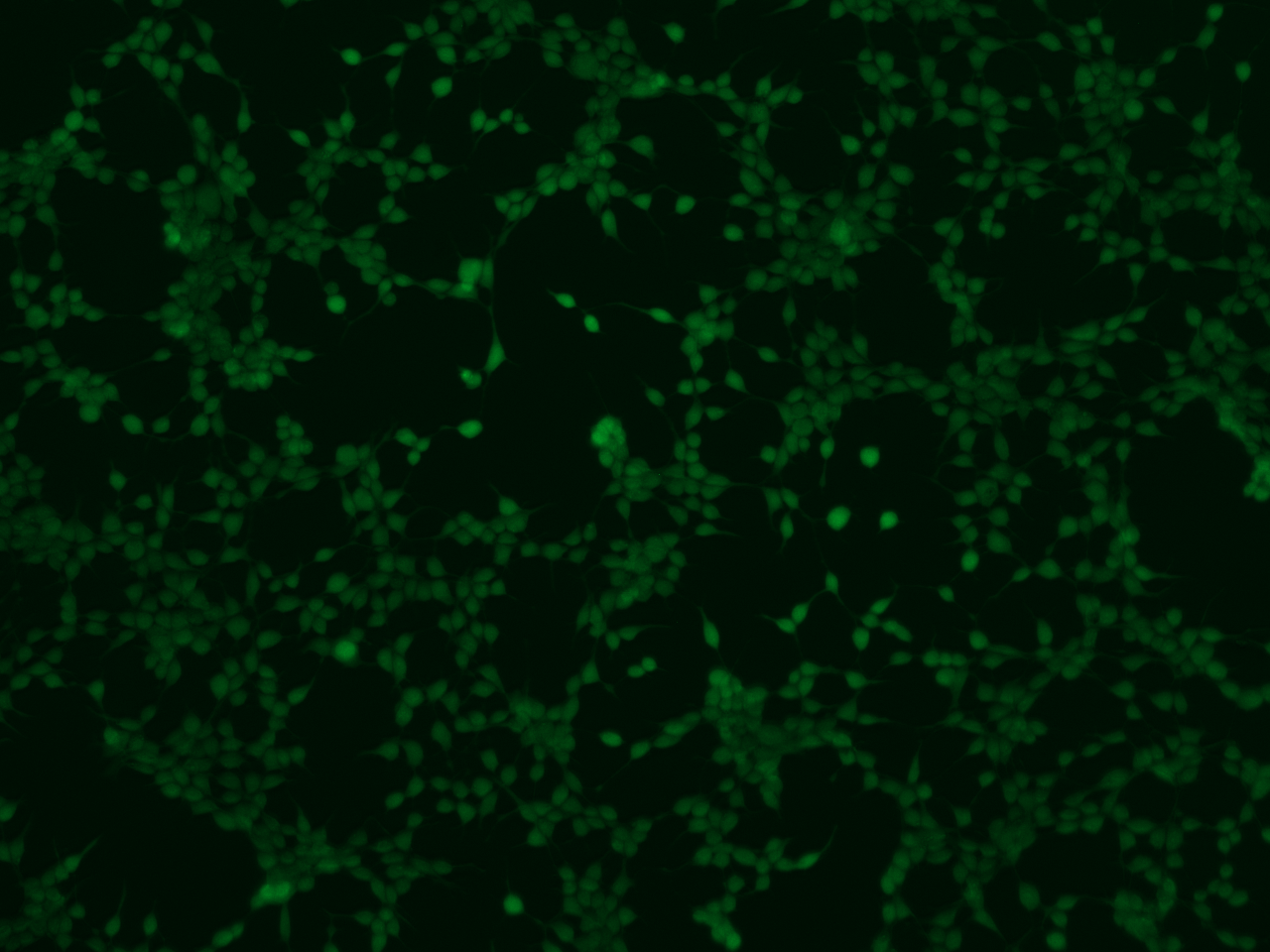

Supplement: Supplementary file 4 — Source Data Fig. 3 [file 44321_2024_35_MOESM4_ESM.zip › Figure 3/3D/5 NOX4Y512C.tif]

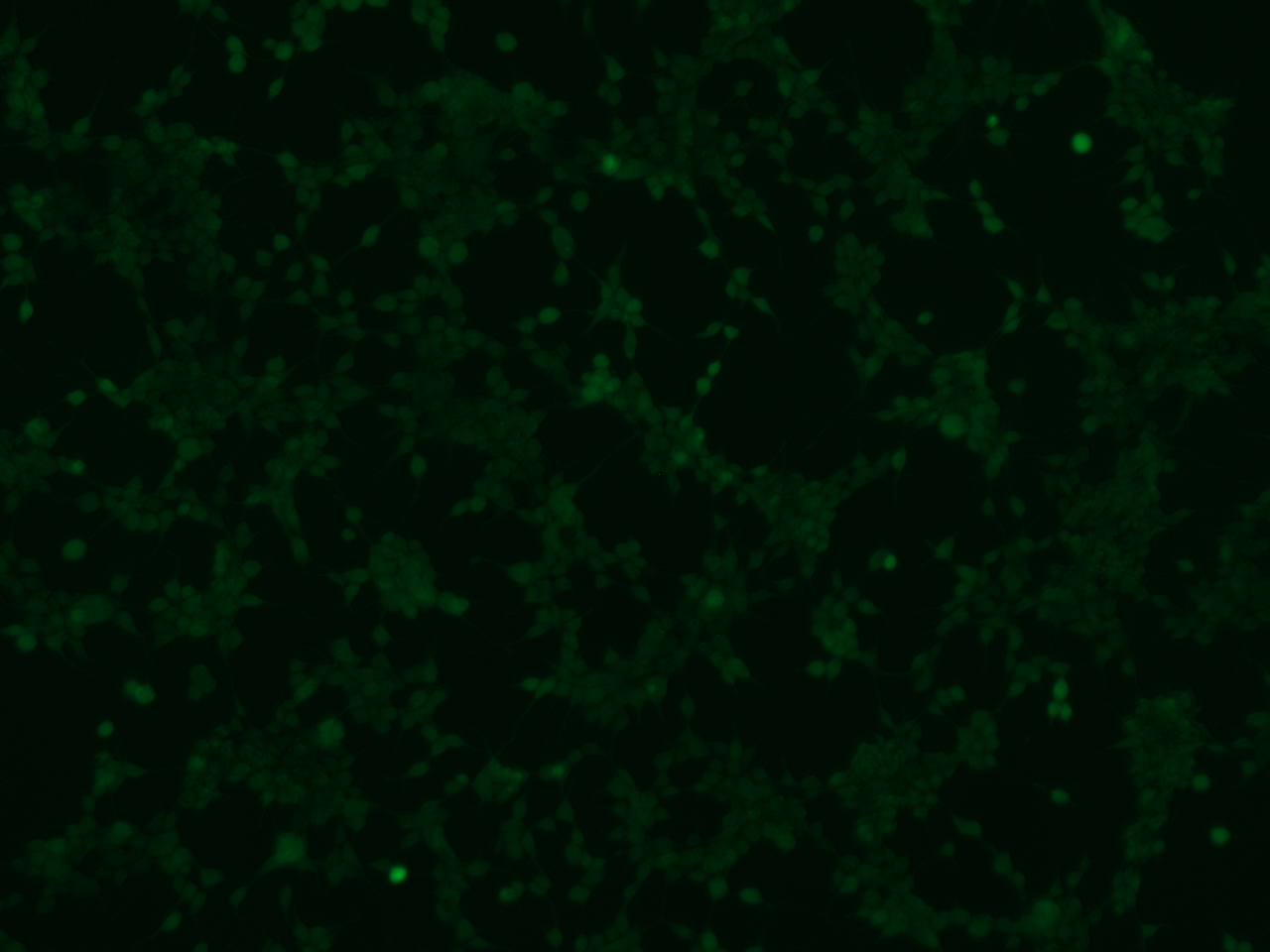

Supplement: Supplementary file 4 — Source Data Fig. 3 [file 44321_2024_35_MOESM4_ESM.zip › Figure 3/3D/1 pcDNA3.1.tif]

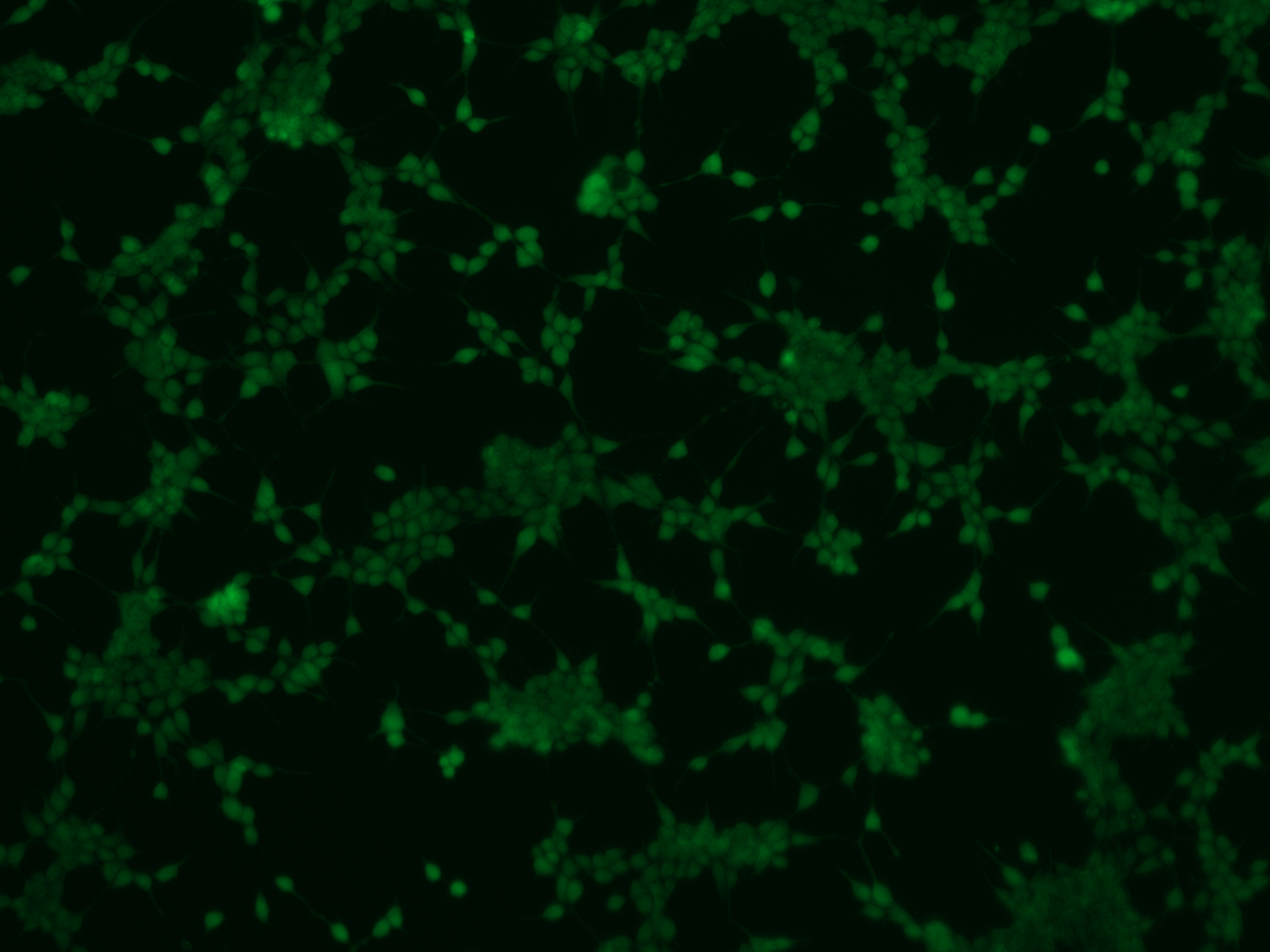

Supplement: Supplementary file 4 — Source Data Fig. 3 [file 44321_2024_35_MOESM4_ESM.zip › Figure 3/3D/3 NOX4Y512IfsX20.tif]

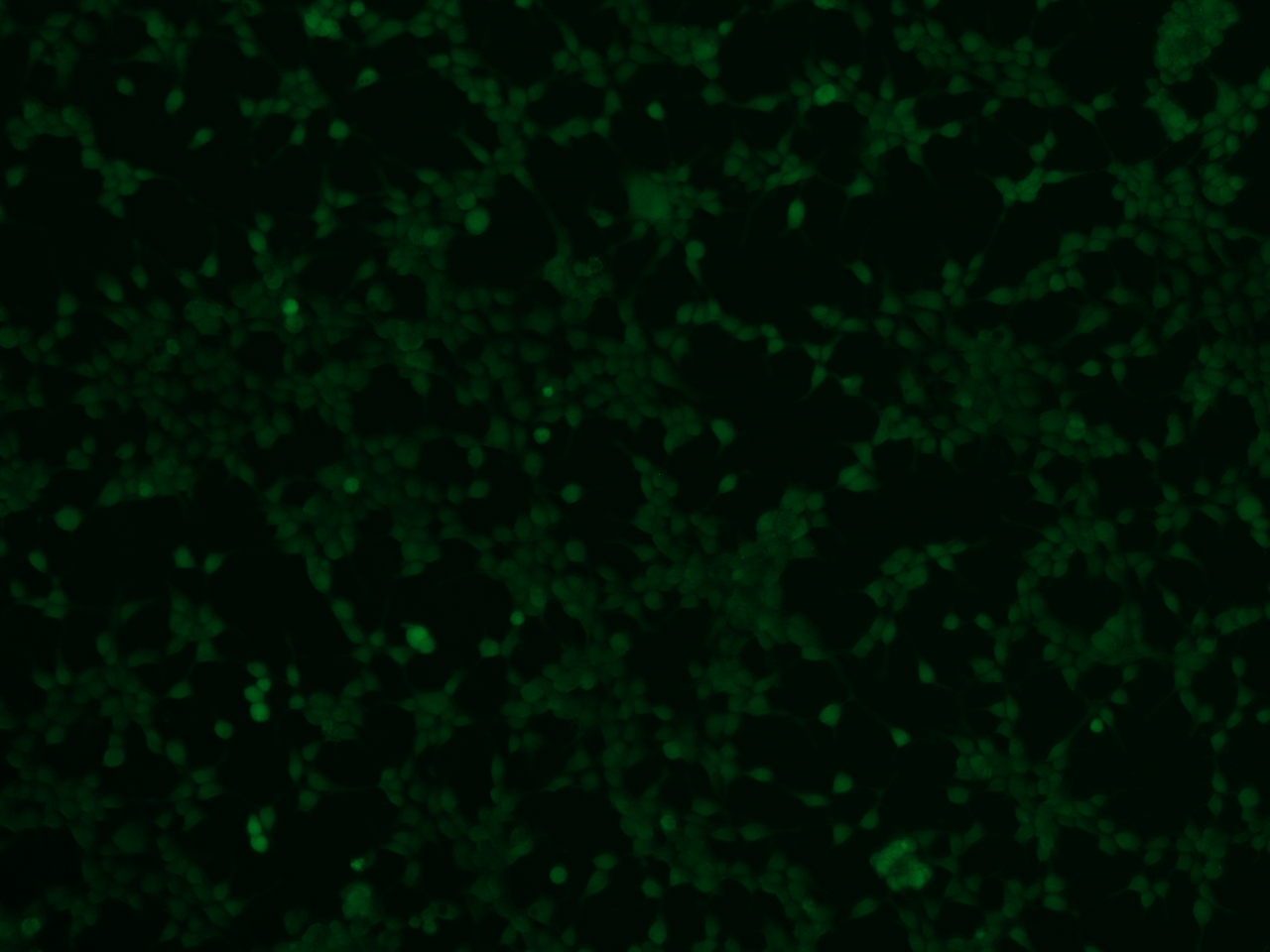

Supplement: Supplementary file 4 — Source Data Fig. 3 [file 44321_2024_35_MOESM4_ESM.zip › Figure 3/3D/2 NOX4WT.tif]

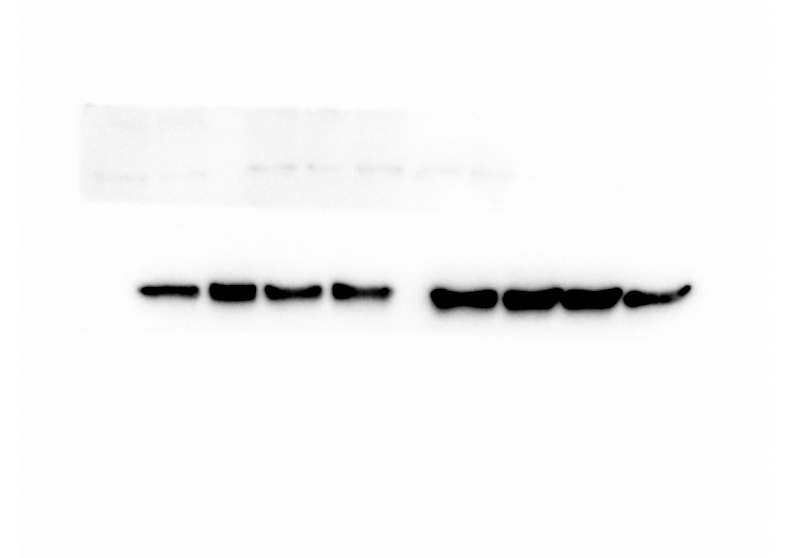

Supplement: Supplementary file 6 — Source Data Fig. 5 [file 44321_2024_35_MOESM6_ESM.zip › Figure 5/5D/western GAPDH left.tif]

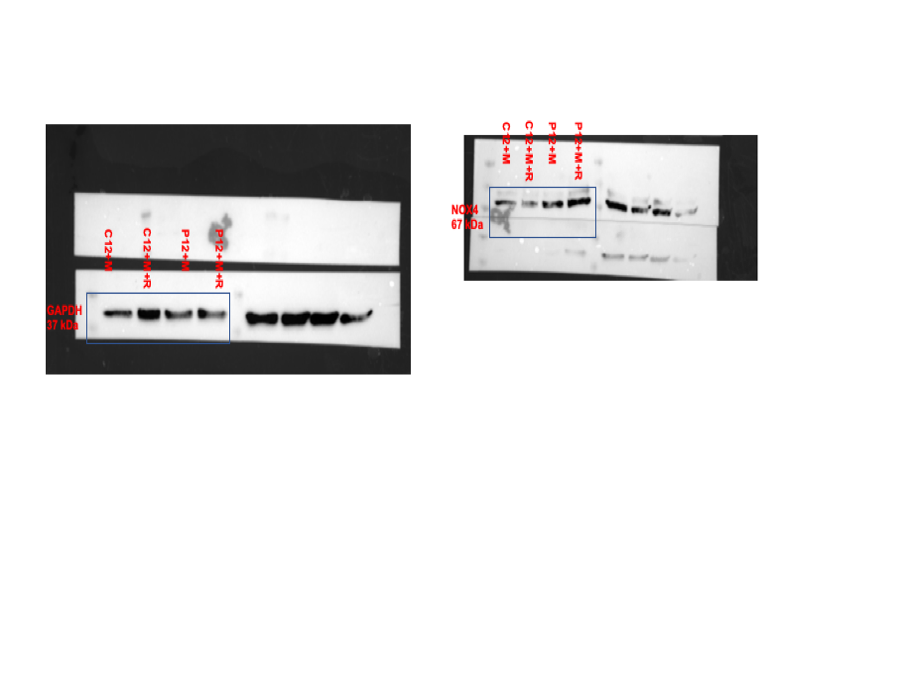

Supplement: Supplementary file 6 — Source Data Fig. 5 [file 44321_2024_35_MOESM6_ESM.zip › Figure 5/5D/wb3 merge with marker.tiff]

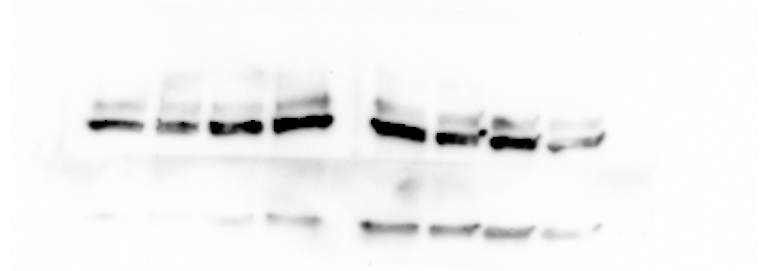

Supplement: Supplementary file 6 — Source Data Fig. 5 [file 44321_2024_35_MOESM6_ESM.zip › Figure 5/5D/western NOX4 left.tif]

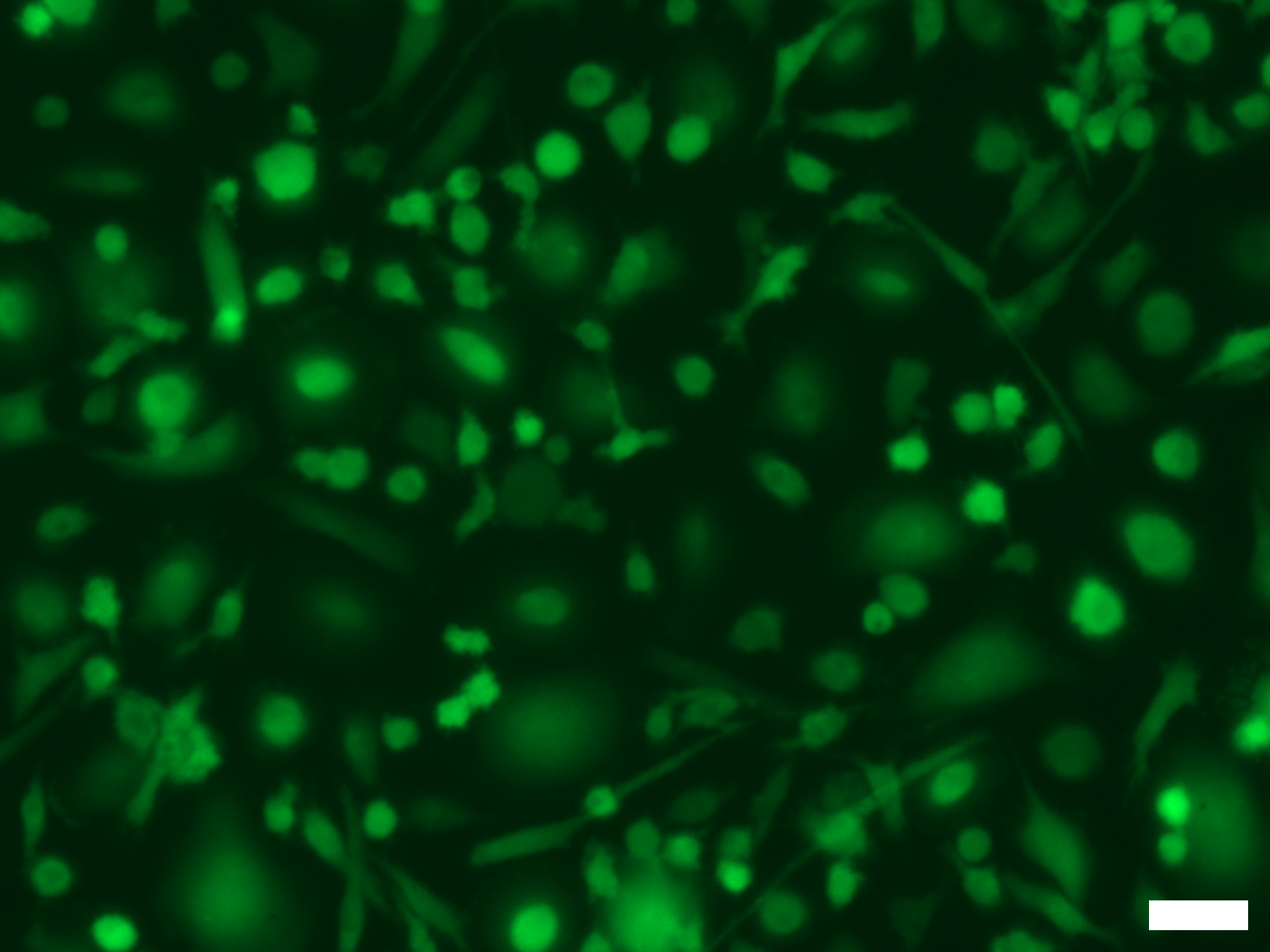

Supplement: Supplementary file 6 — Source Data Fig. 5 [file 44321_2024_35_MOESM6_ESM.zip › Figure 5/5E/Day8 PAM12 M-CSF+RANKL.tif]

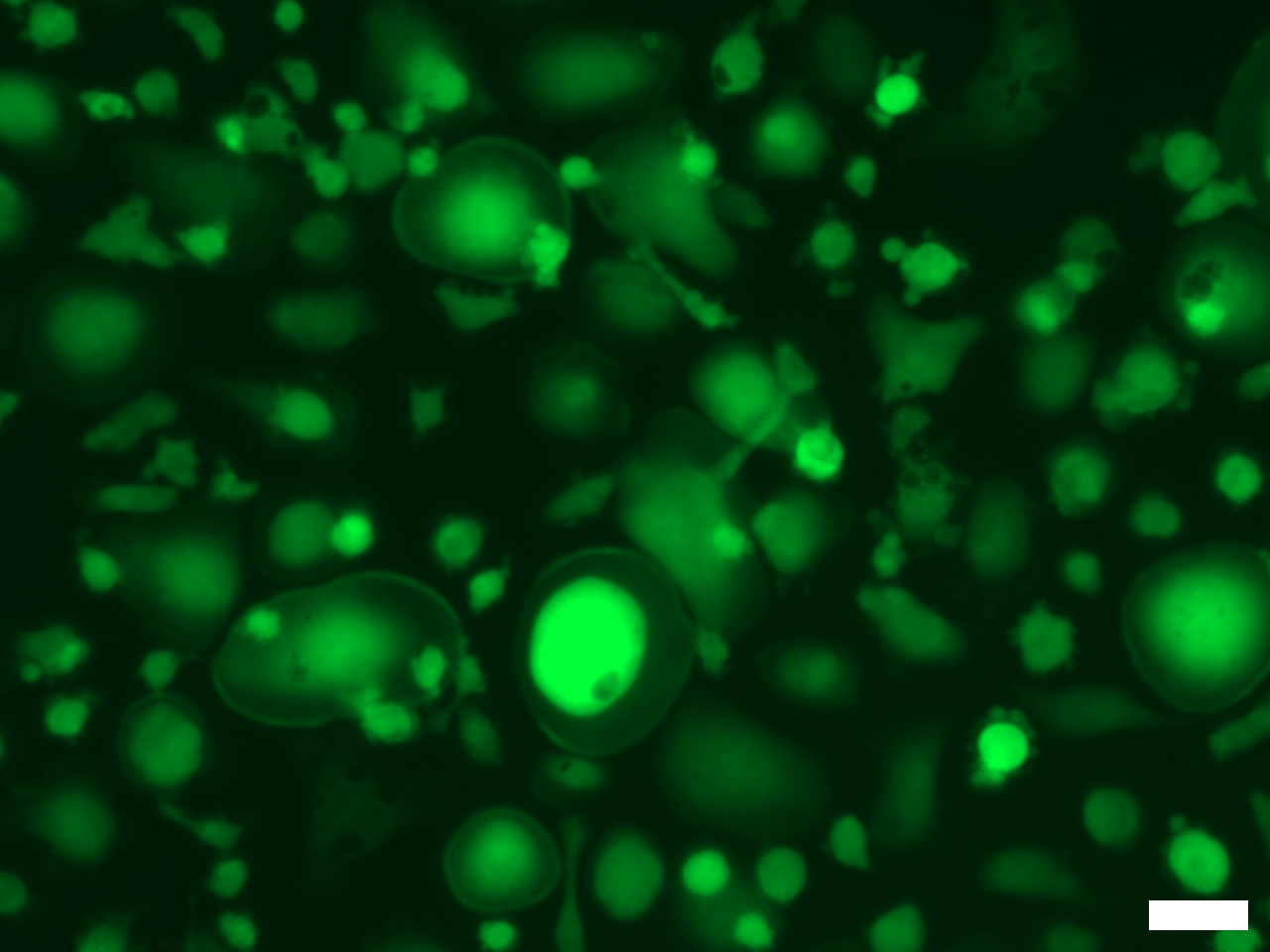

Supplement: Supplementary file 6 — Source Data Fig. 5 [file 44321_2024_35_MOESM6_ESM.zip › Figure 5/5E/Day12 PAM12 M-CSF+RANKL.tif]

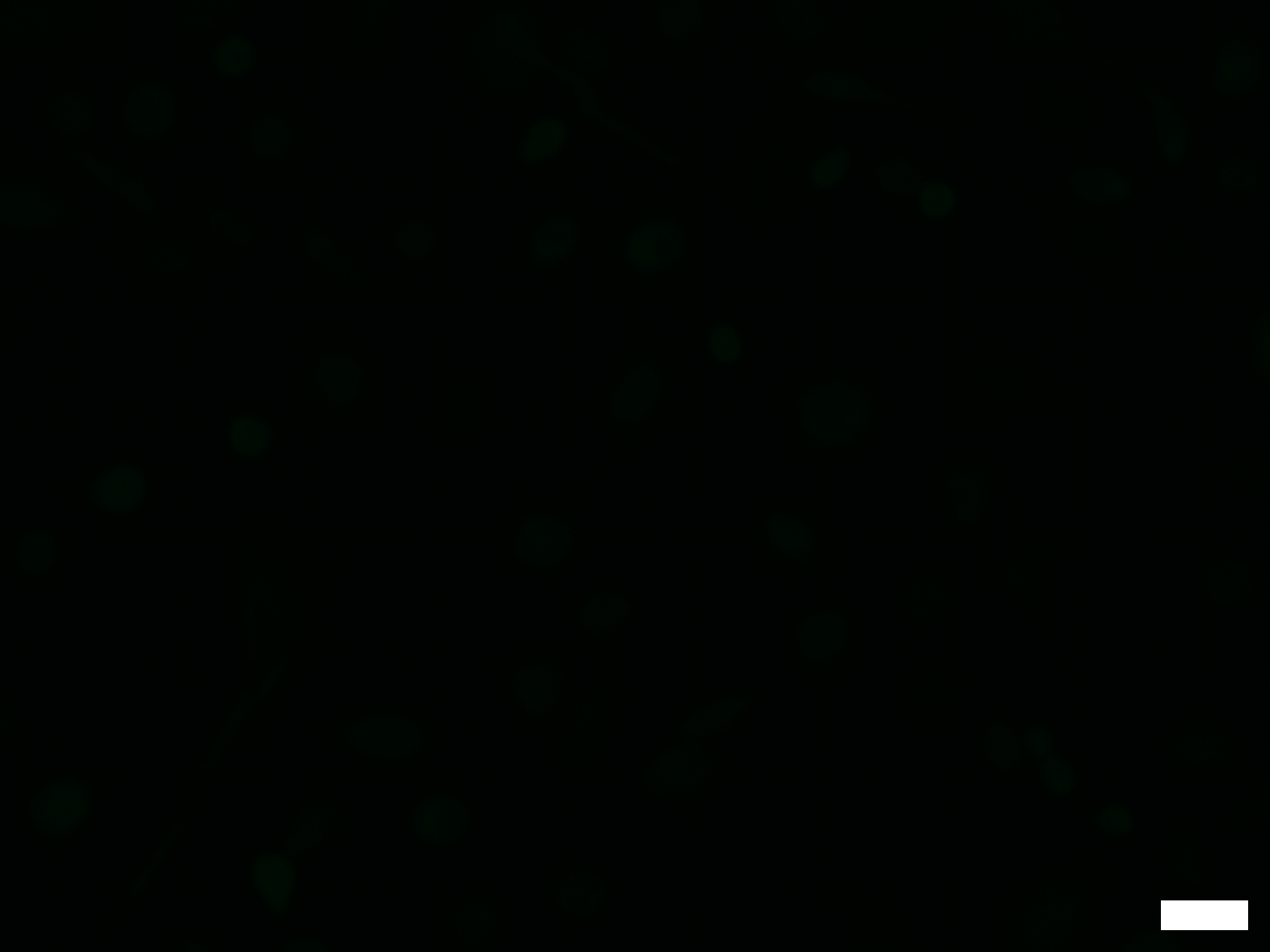

Supplement: Supplementary file 6 — Source Data Fig. 5 [file 44321_2024_35_MOESM6_ESM.zip › Figure 5/5E/Day8 C12 M-CSF.tif]

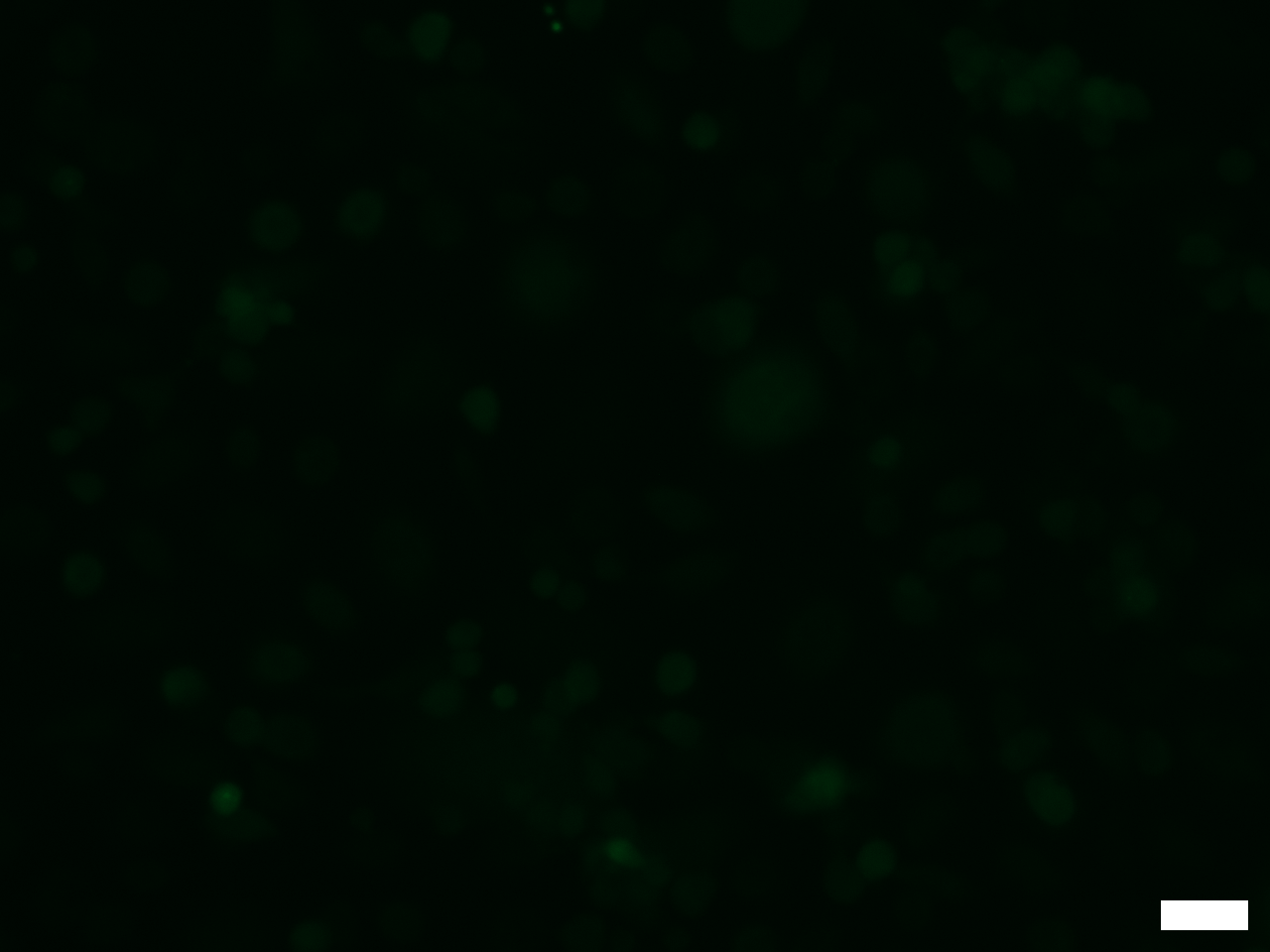

Supplement: Supplementary file 6 — Source Data Fig. 5 [file 44321_2024_35_MOESM6_ESM.zip › Figure 5/5E/Day8 C12 M-CSF+RANKL.tif]

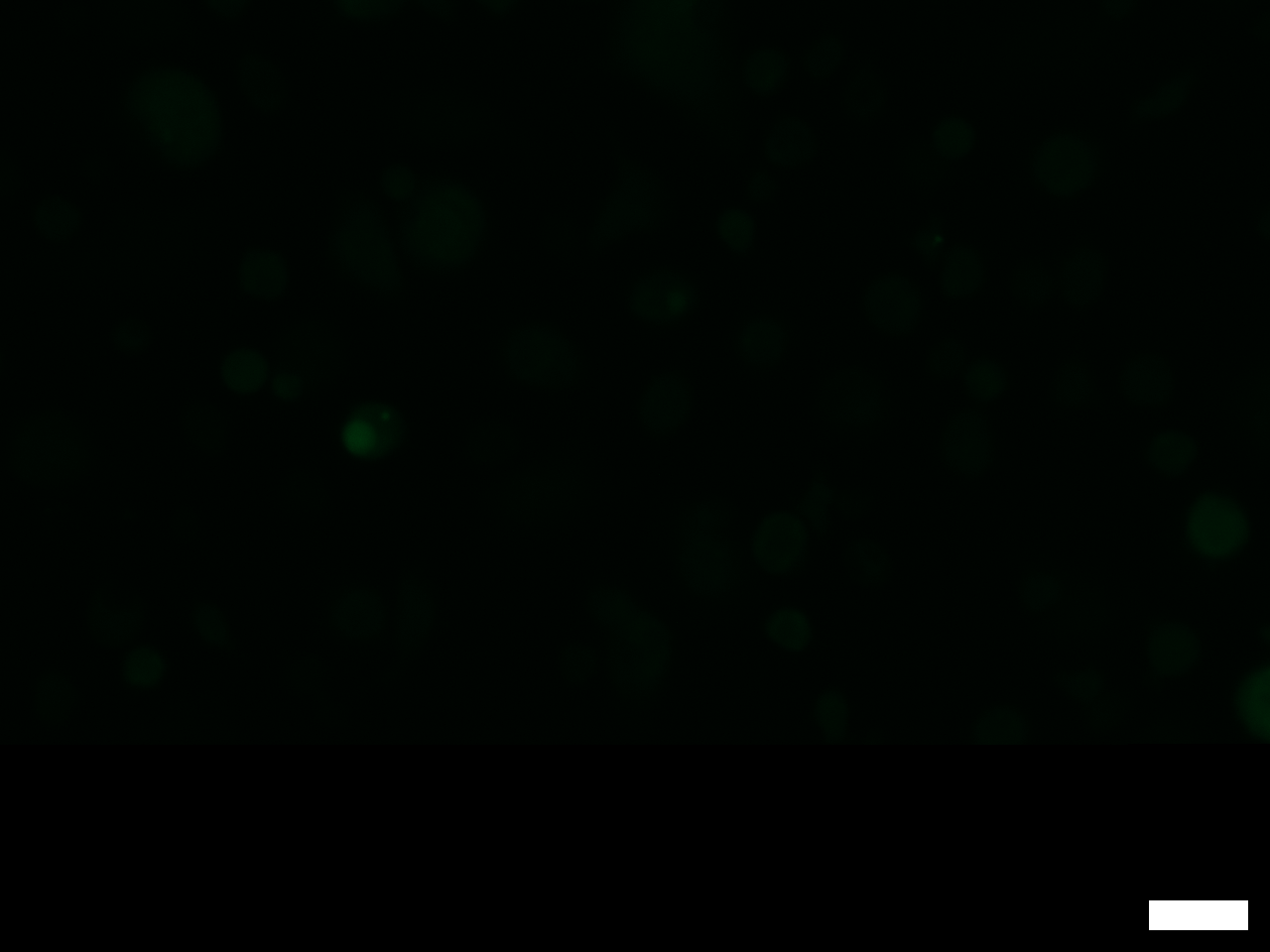

Supplement: Supplementary file 6 — Source Data Fig. 5 [file 44321_2024_35_MOESM6_ESM.zip › Figure 5/5E/Day12 C12 M-CSF.tif]

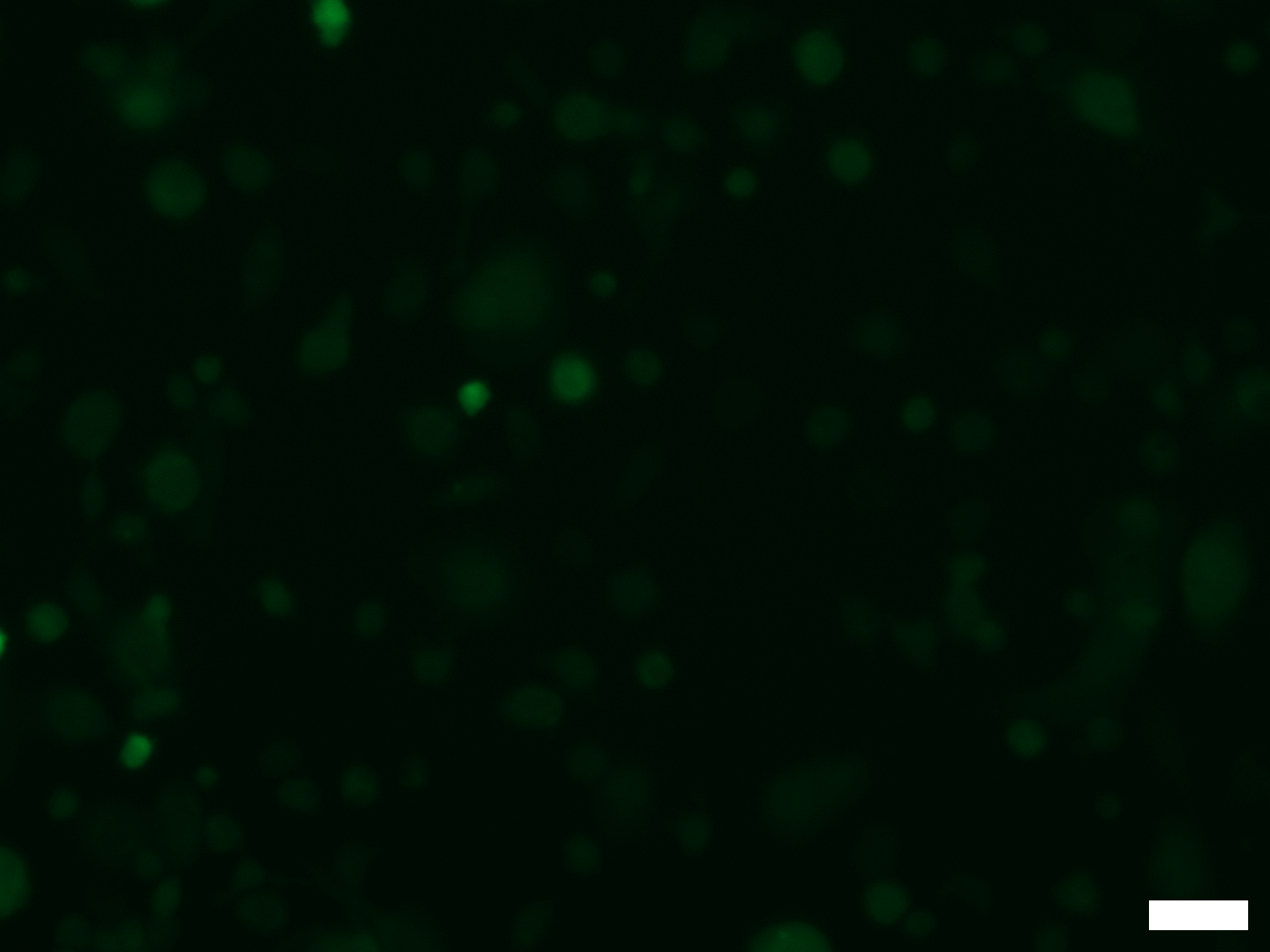

Supplement: Supplementary file 6 — Source Data Fig. 5 [file 44321_2024_35_MOESM6_ESM.zip › Figure 5/5E/Day12 PAM12 M-CSF.tif]

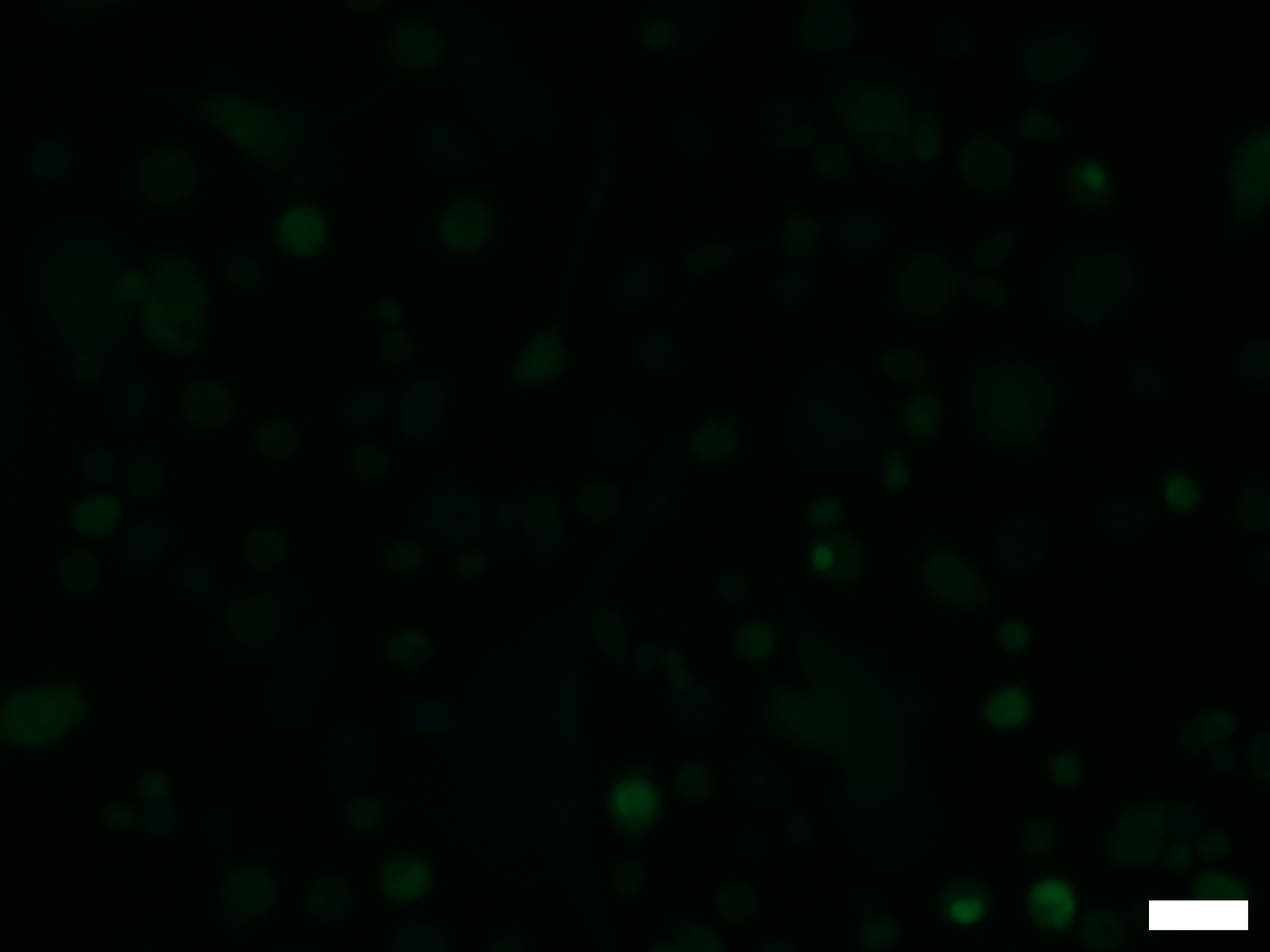

Supplement: Supplementary file 6 — Source Data Fig. 5 [file 44321_2024_35_MOESM6_ESM.zip › Figure 5/5E/Day12 C12 M-CSF+RANKL.tif]

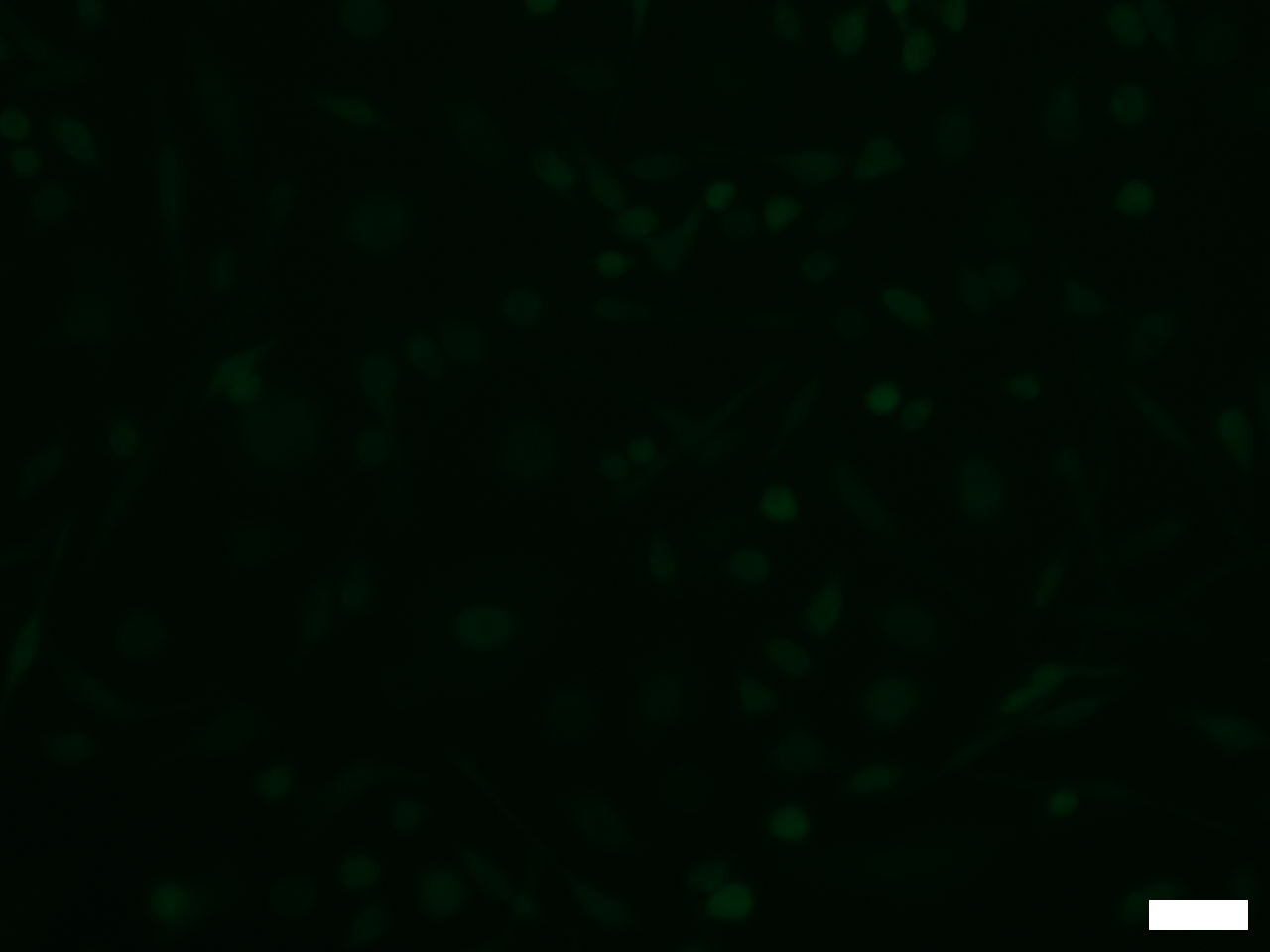

Supplement: Supplementary file 6 — Source Data Fig. 5 [file 44321_2024_35_MOESM6_ESM.zip › Figure 5/5E/Day8 PAM12 M-CSF.tif]

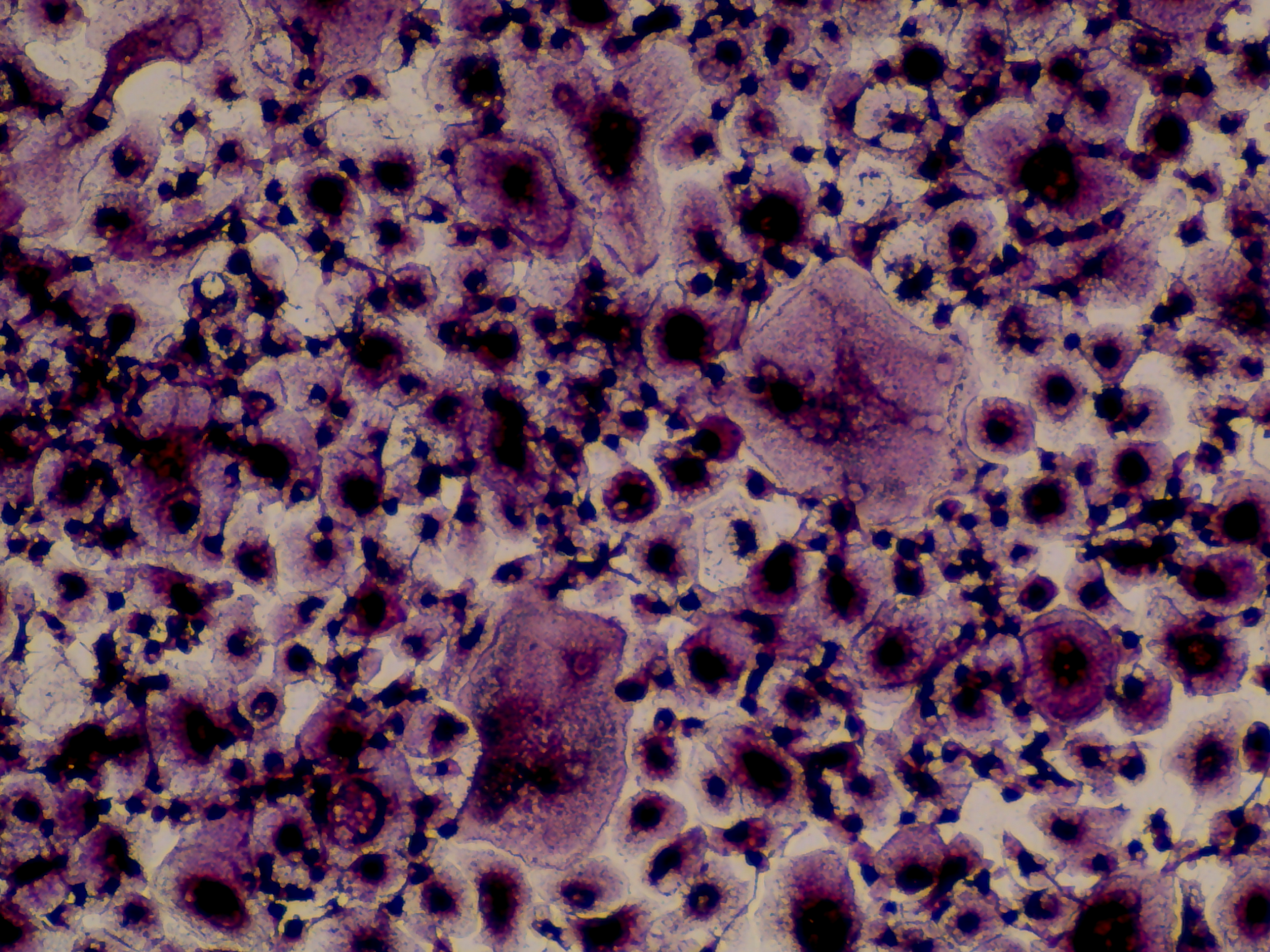

Supplement: Supplementary file 6 — Source Data Fig. 5 [file 44321_2024_35_MOESM6_ESM.zip › Figure 5/5B/Day12 PAM 12 M-CSF+RANKL.tif]

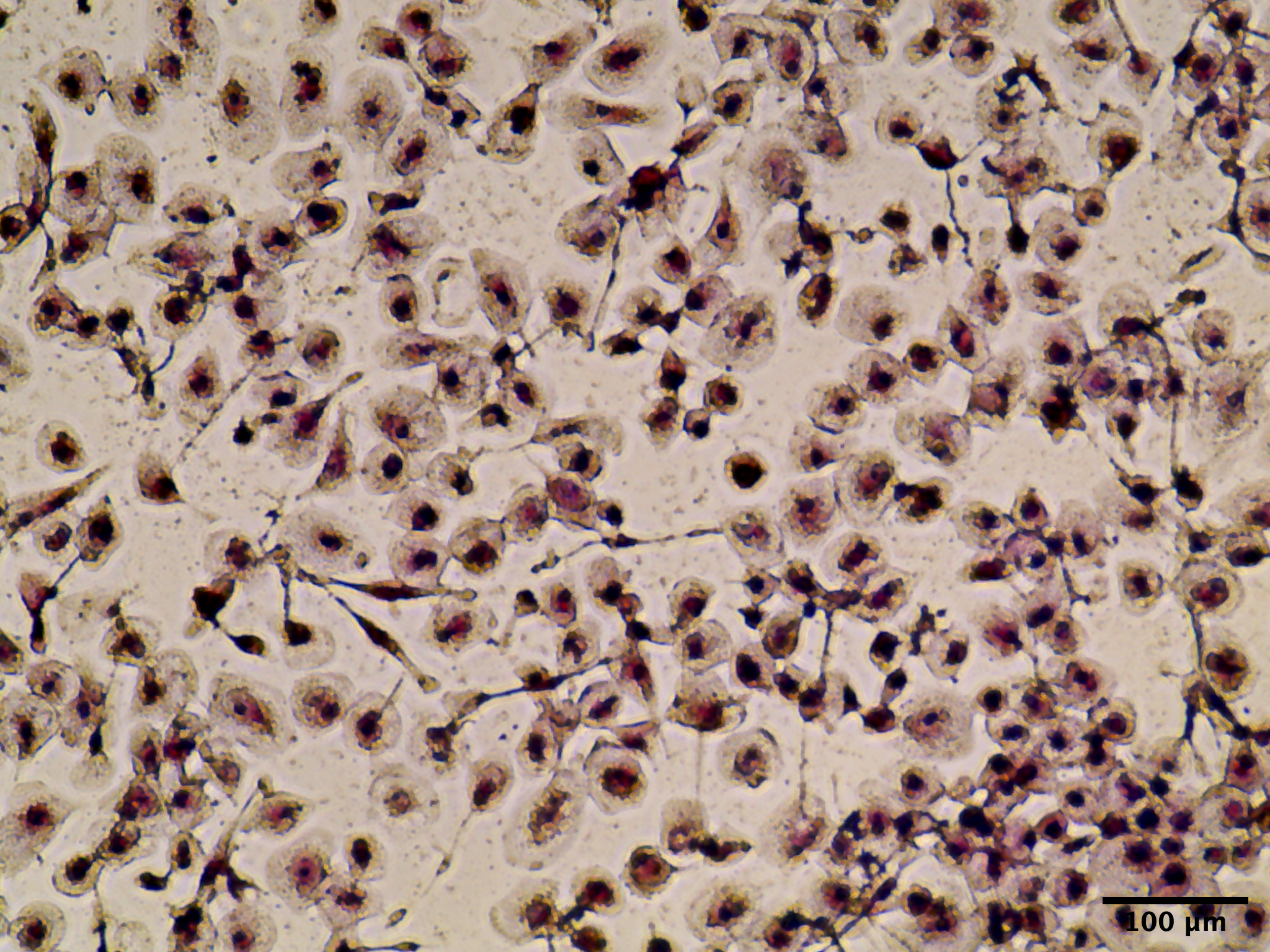

Supplement: Supplementary file 6 — Source Data Fig. 5 [file 44321_2024_35_MOESM6_ESM.zip › Figure 5/5B/Day8 C12 M-CSF.tif]

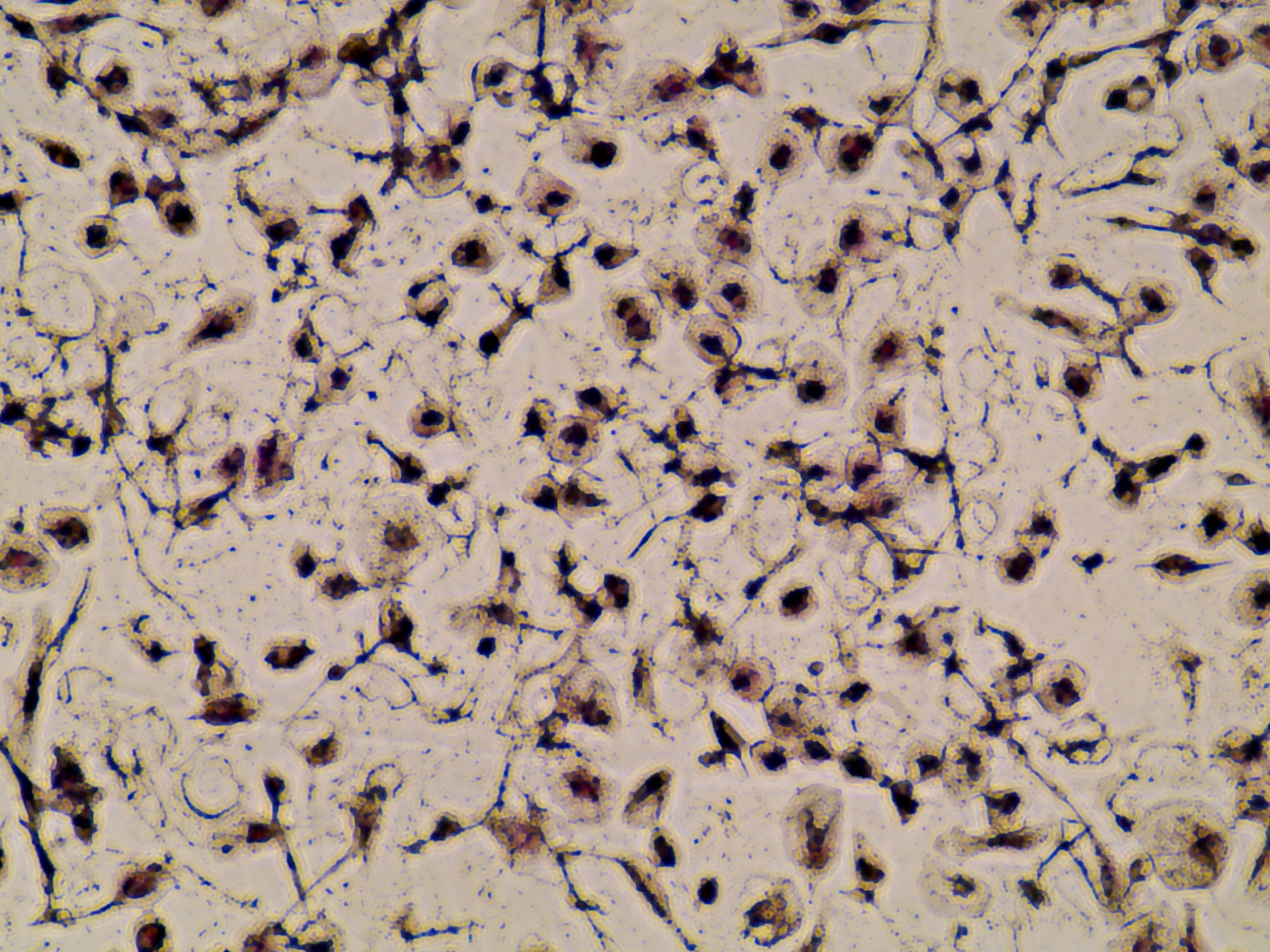

Supplement: Supplementary file 6 — Source Data Fig. 5 [file 44321_2024_35_MOESM6_ESM.zip › Figure 5/5B/Day8 C12 M-CSF+RANKL.tif]

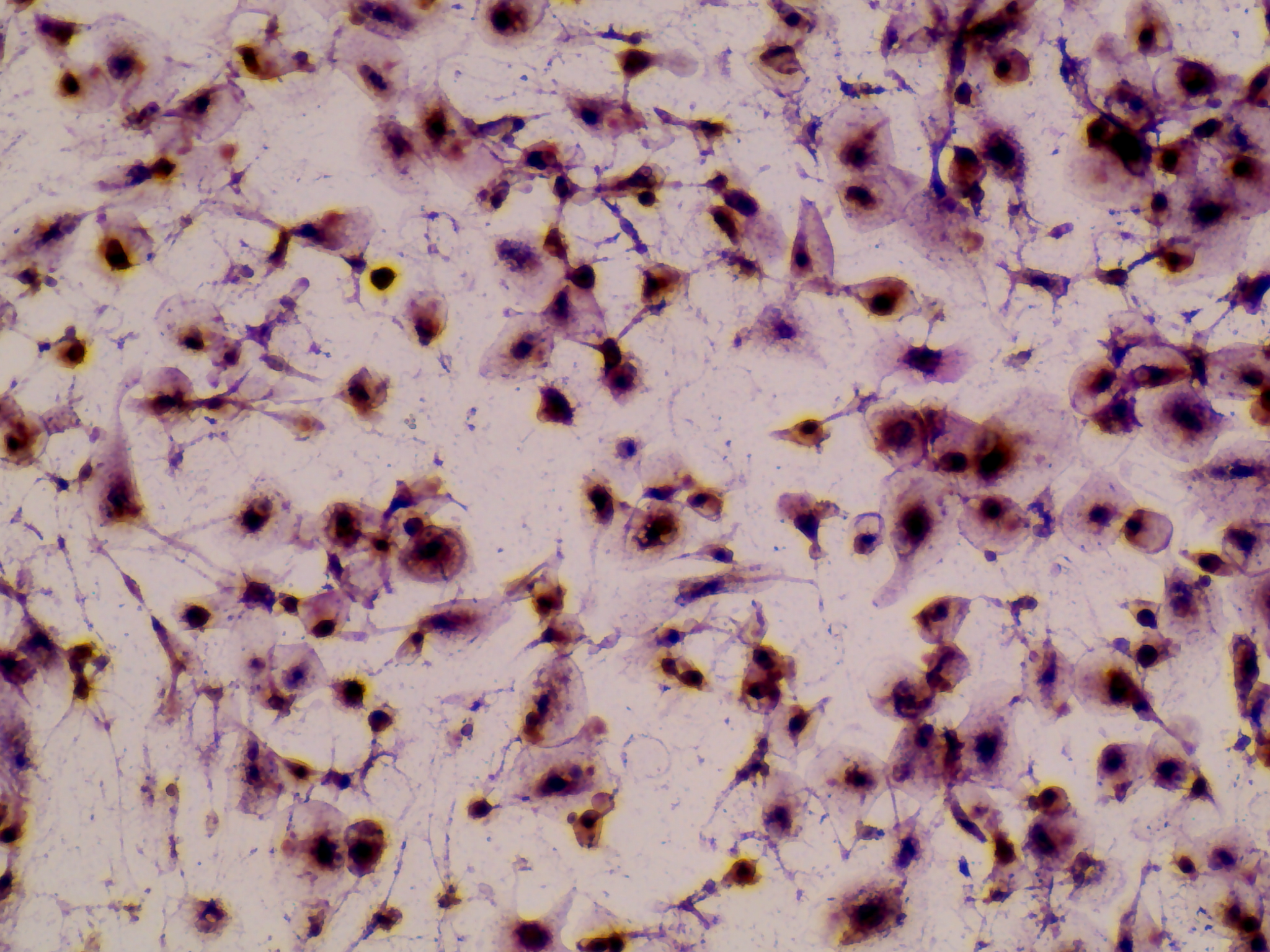

Supplement: Supplementary file 6 — Source Data Fig. 5 [file 44321_2024_35_MOESM6_ESM.zip › Figure 5/5B/Day12 C12 M-CSF.tif]

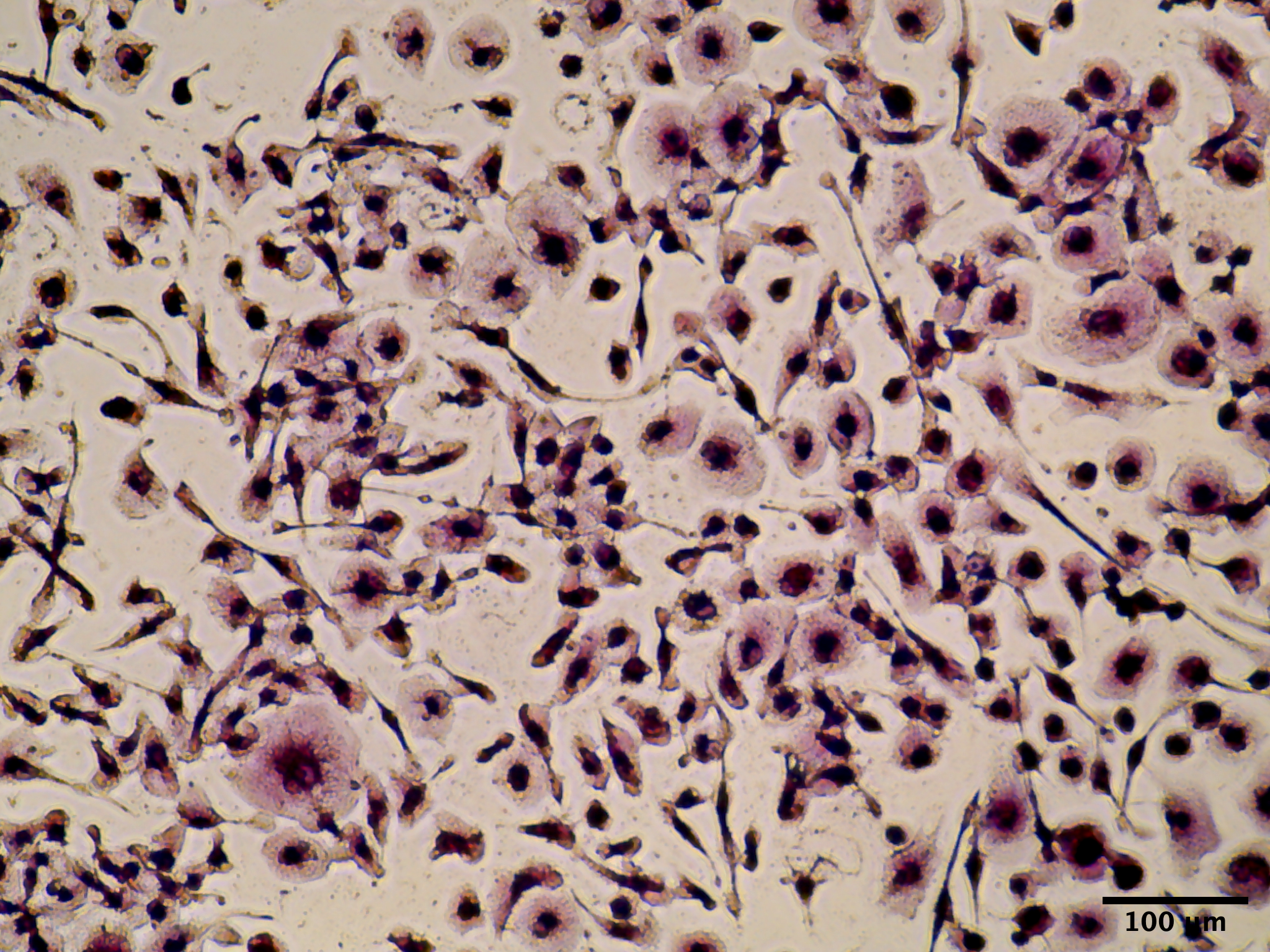

Supplement: Supplementary file 6 — Source Data Fig. 5 [file 44321_2024_35_MOESM6_ESM.zip › Figure 5/5B/Day8 PAM 12 M-CSF+RANKL.tif]

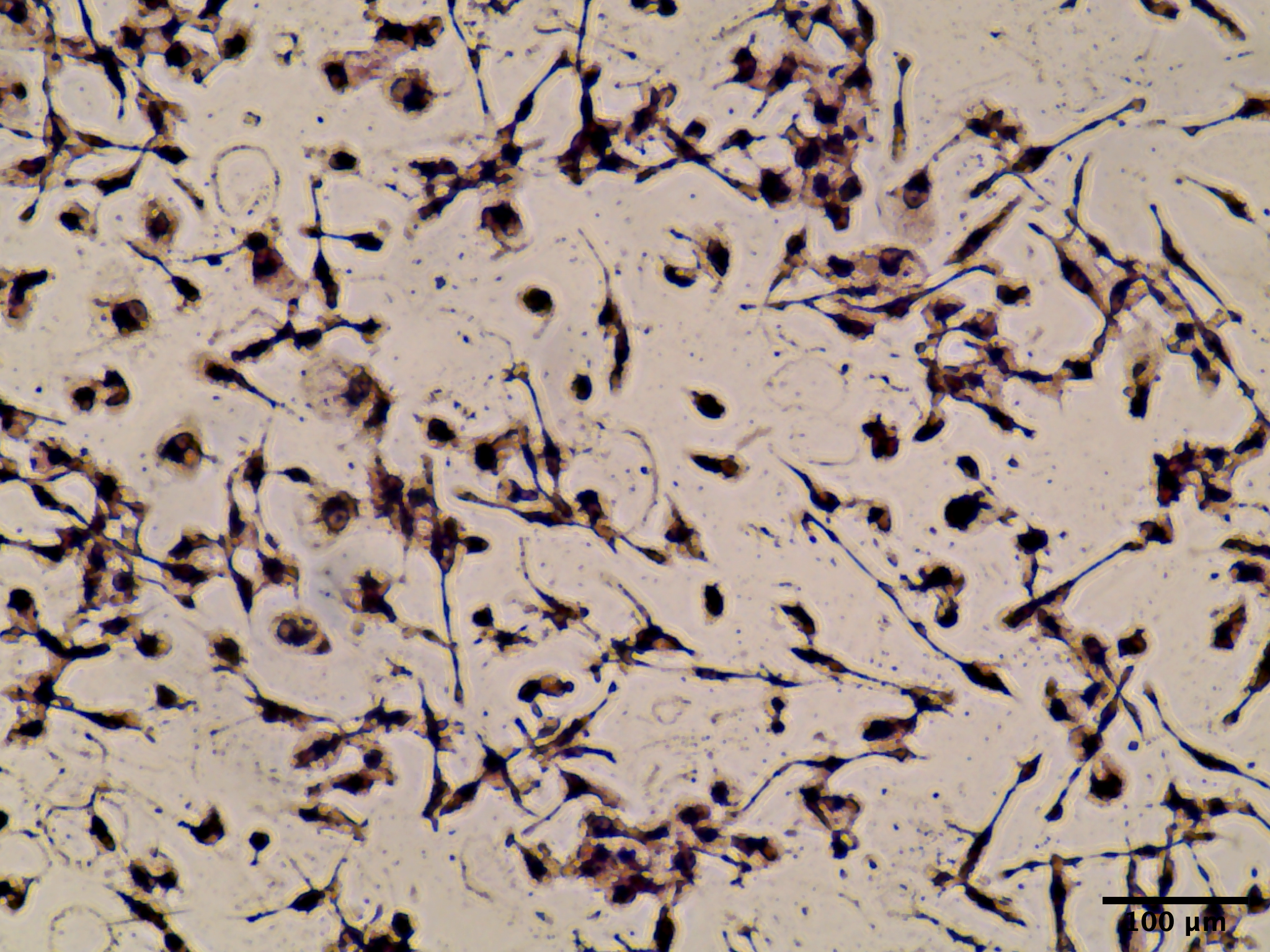

Supplement: Supplementary file 6 — Source Data Fig. 5 [file 44321_2024_35_MOESM6_ESM.zip › Figure 5/5B/Day8 PAM 12 M-CSF.tif]

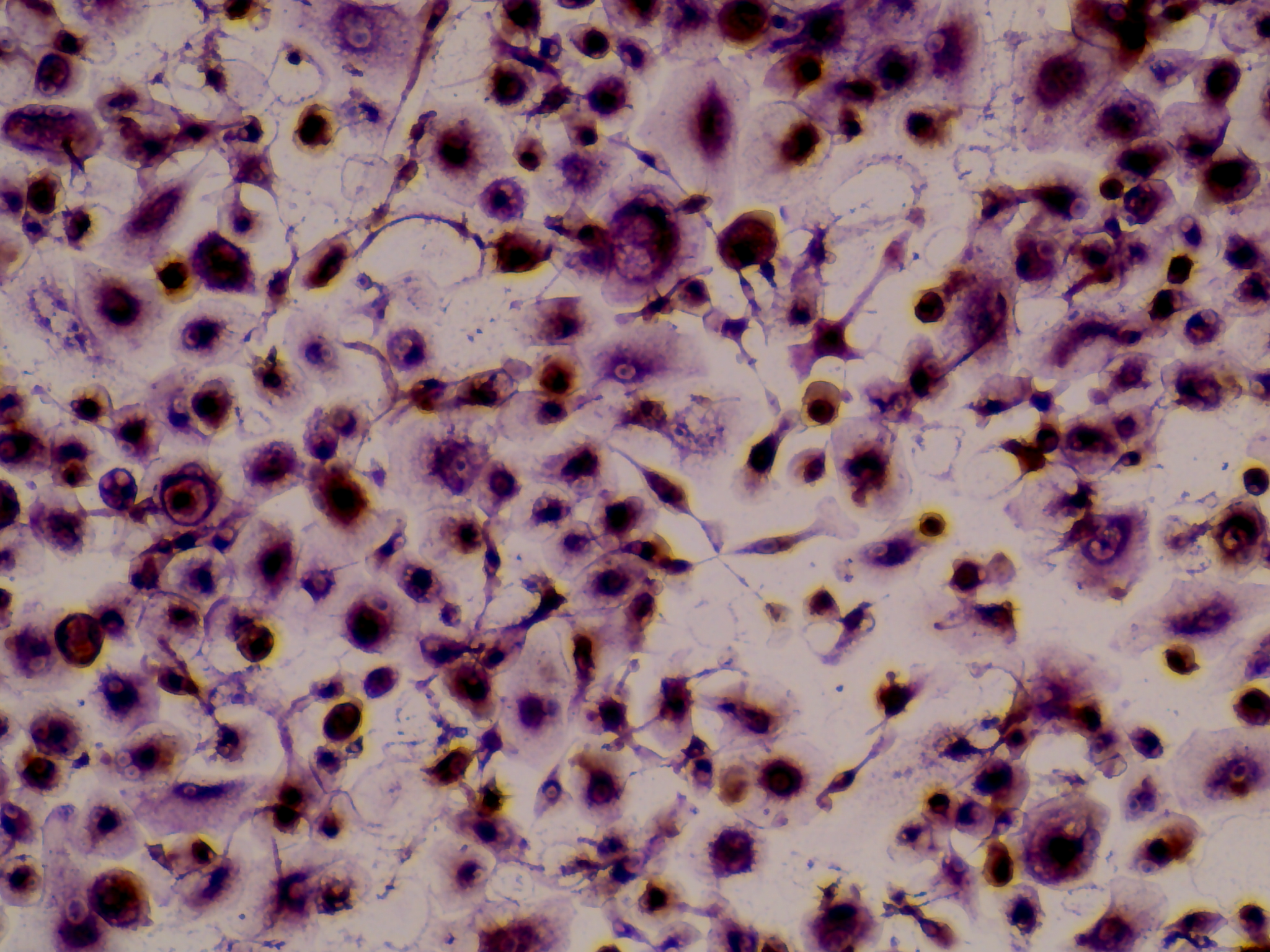

Supplement: Supplementary file 6 — Source Data Fig. 5 [file 44321_2024_35_MOESM6_ESM.zip › Figure 5/5B/Day12 C12 M-CSF+RANKL.tif]

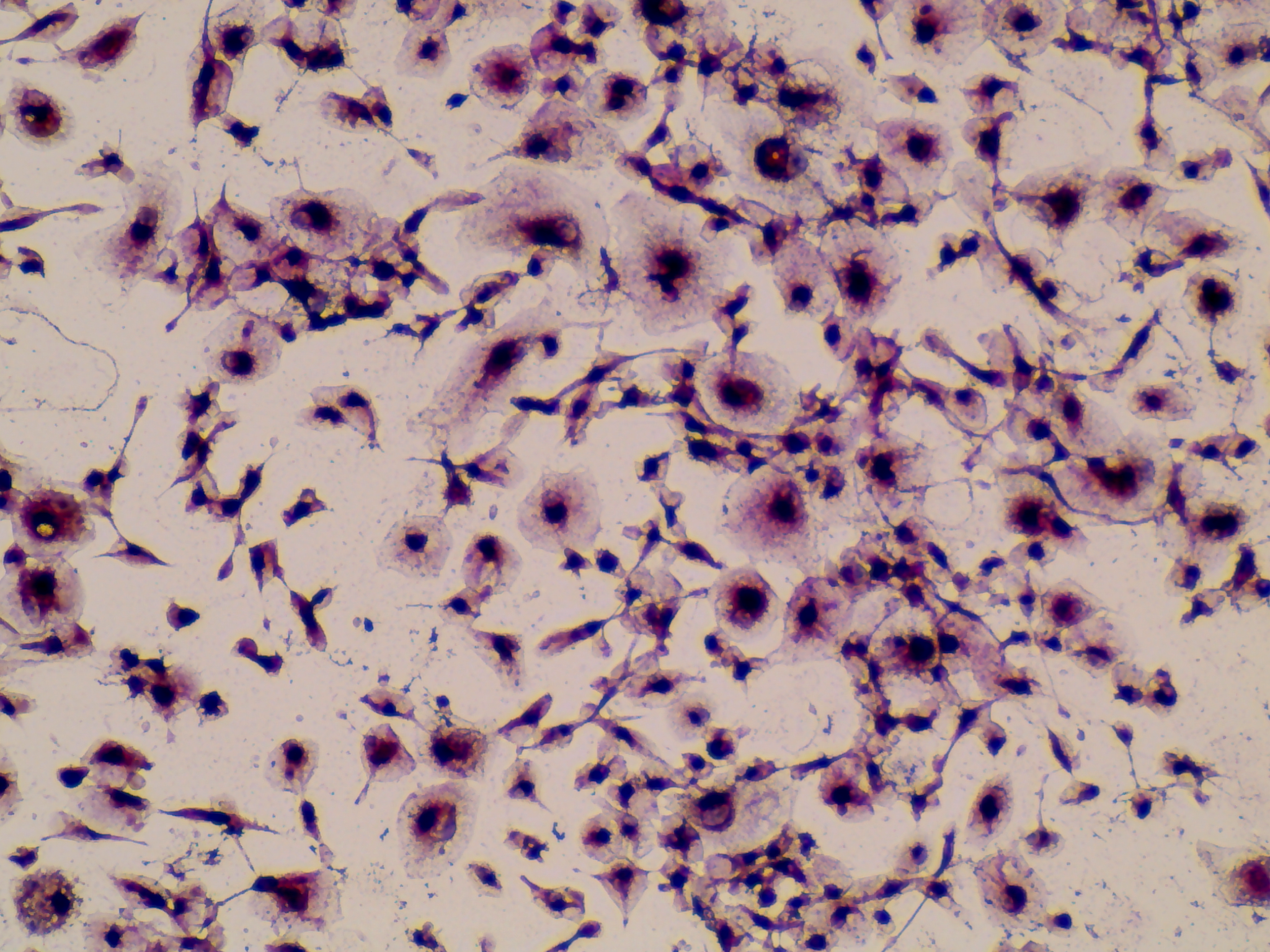

Supplement: Supplementary file 6 — Source Data Fig. 5 [file 44321_2024_35_MOESM6_ESM.zip › Figure 5/5B/Day12 PAM12 M-CSF.tif]

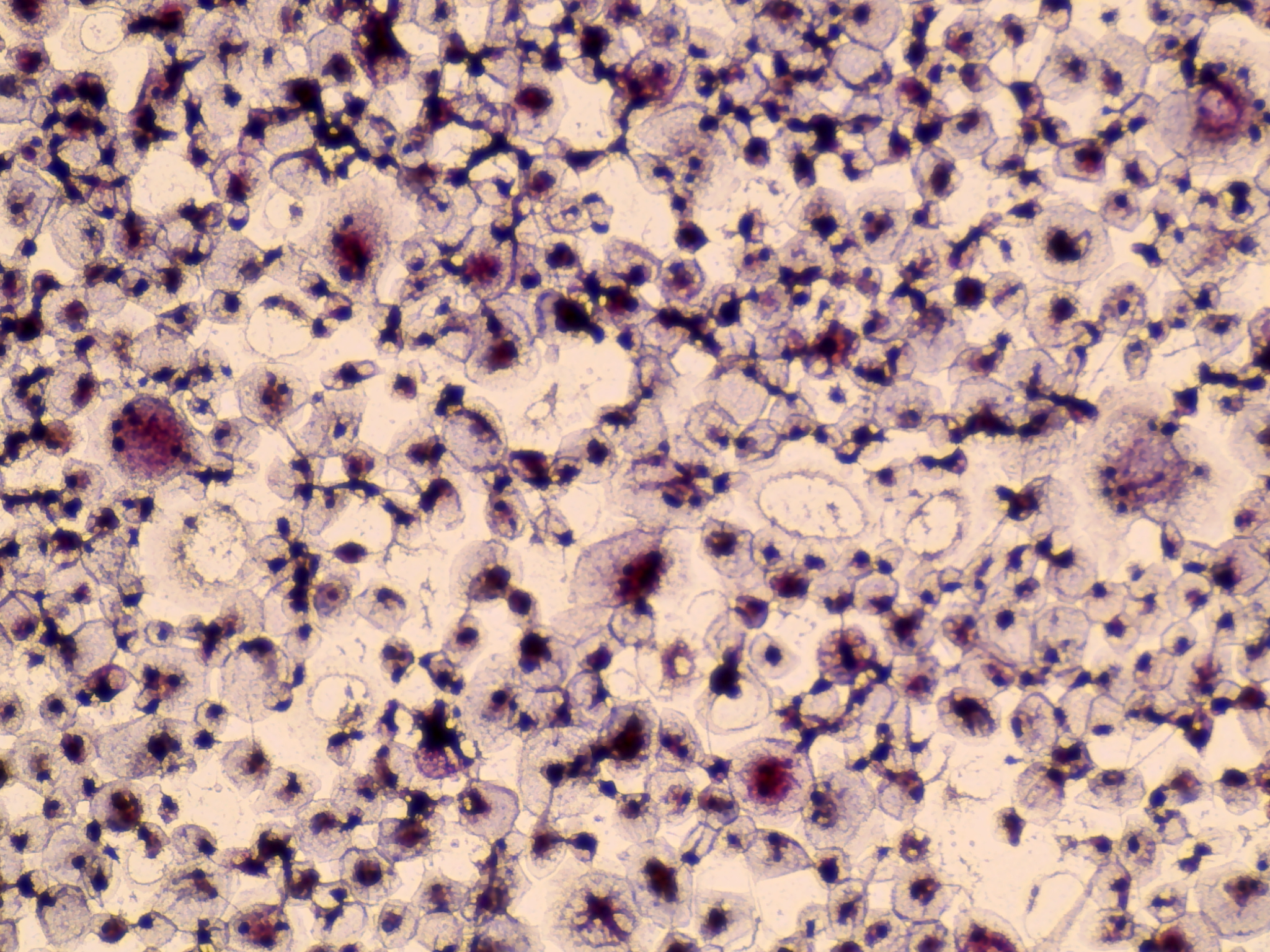

Supplement: Supplementary file 7 — Source Data Fig. 6 [file 44321_2024_35_MOESM7_ESM.zip › Figure 6/6A/Day11 C17-M-R.tif]

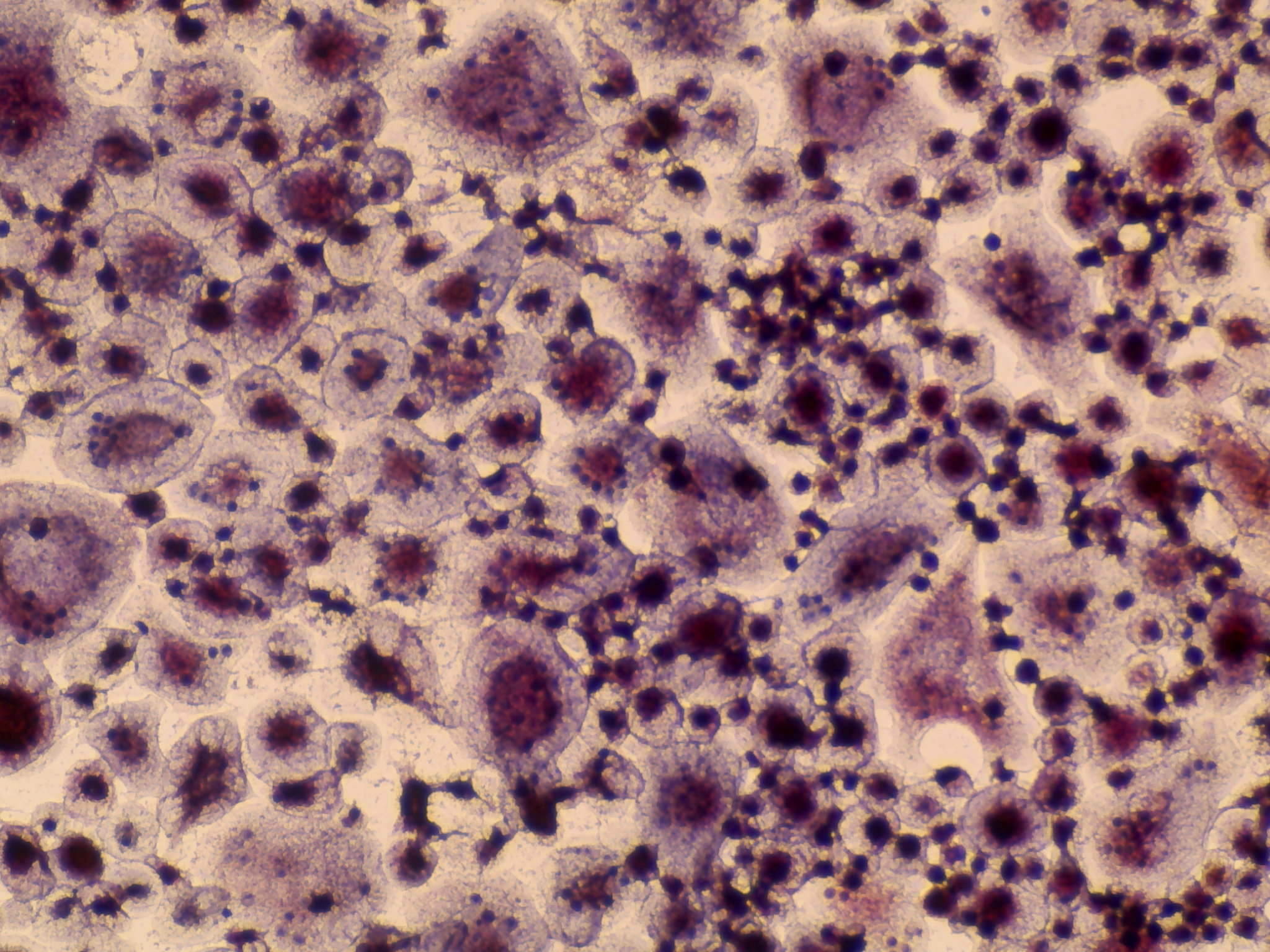

Supplement: Supplementary file 7 — Source Data Fig. 6 [file 44321_2024_35_MOESM7_ESM.zip › Figure 6/6A/Day11 PsA961-M-R.tif]

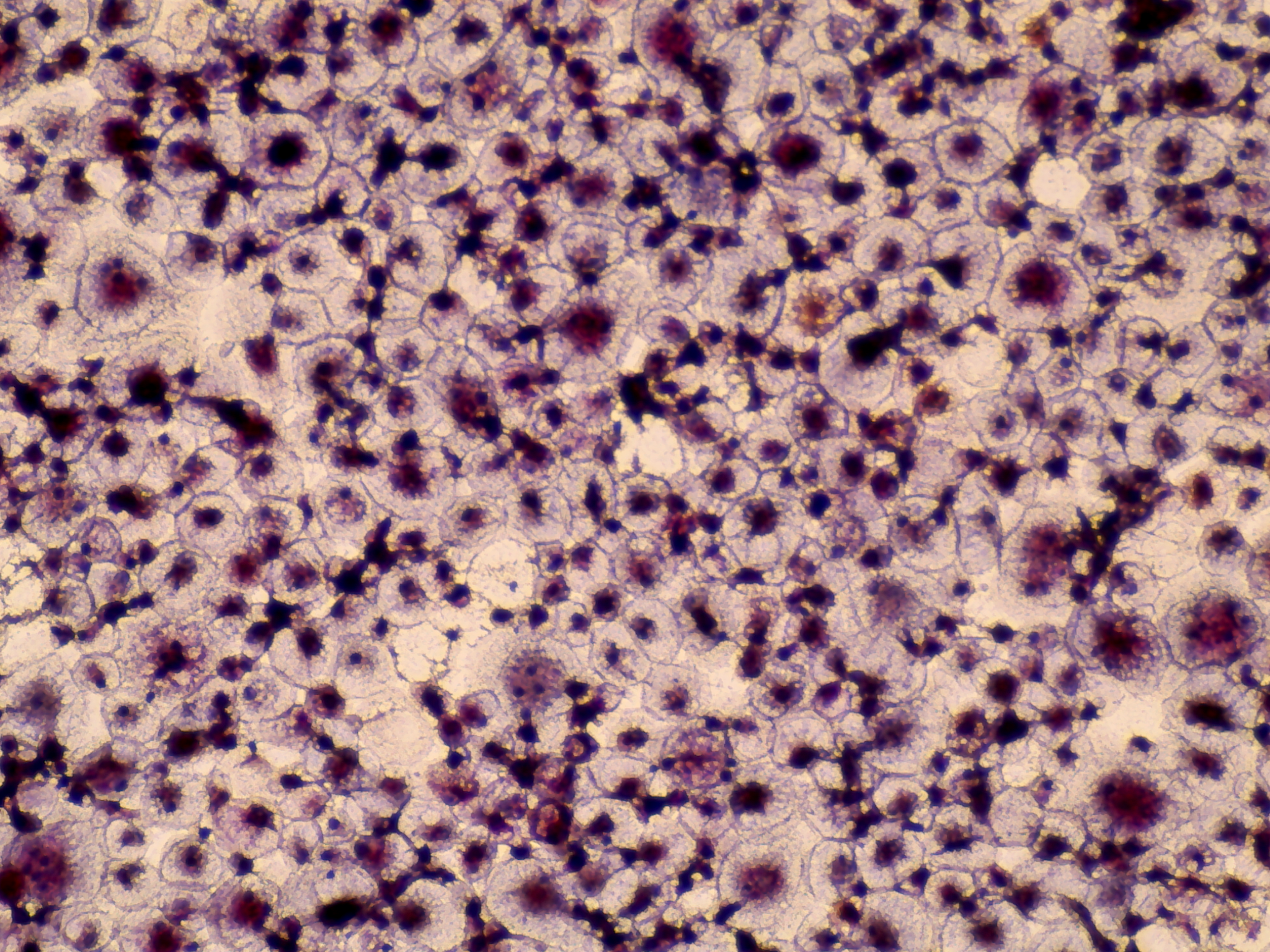

Supplement: Supplementary file 7 — Source Data Fig. 6 [file 44321_2024_35_MOESM7_ESM.zip › Figure 6/6A/Day11 PsA77-M -R.tif]

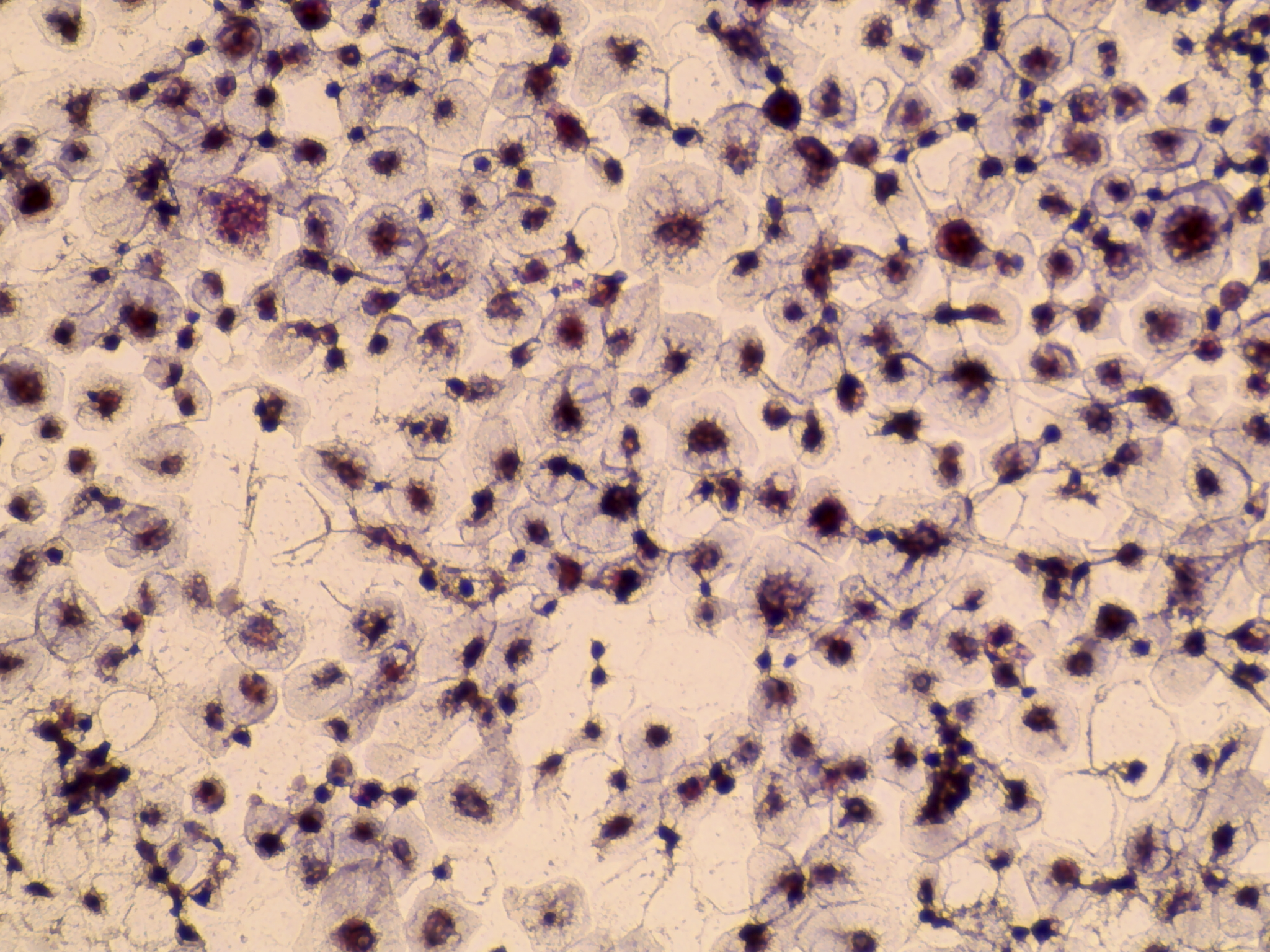

Supplement: Supplementary file 7 — Source Data Fig. 6 [file 44321_2024_35_MOESM7_ESM.zip › Figure 6/6A/Day11 C17-M.tif]

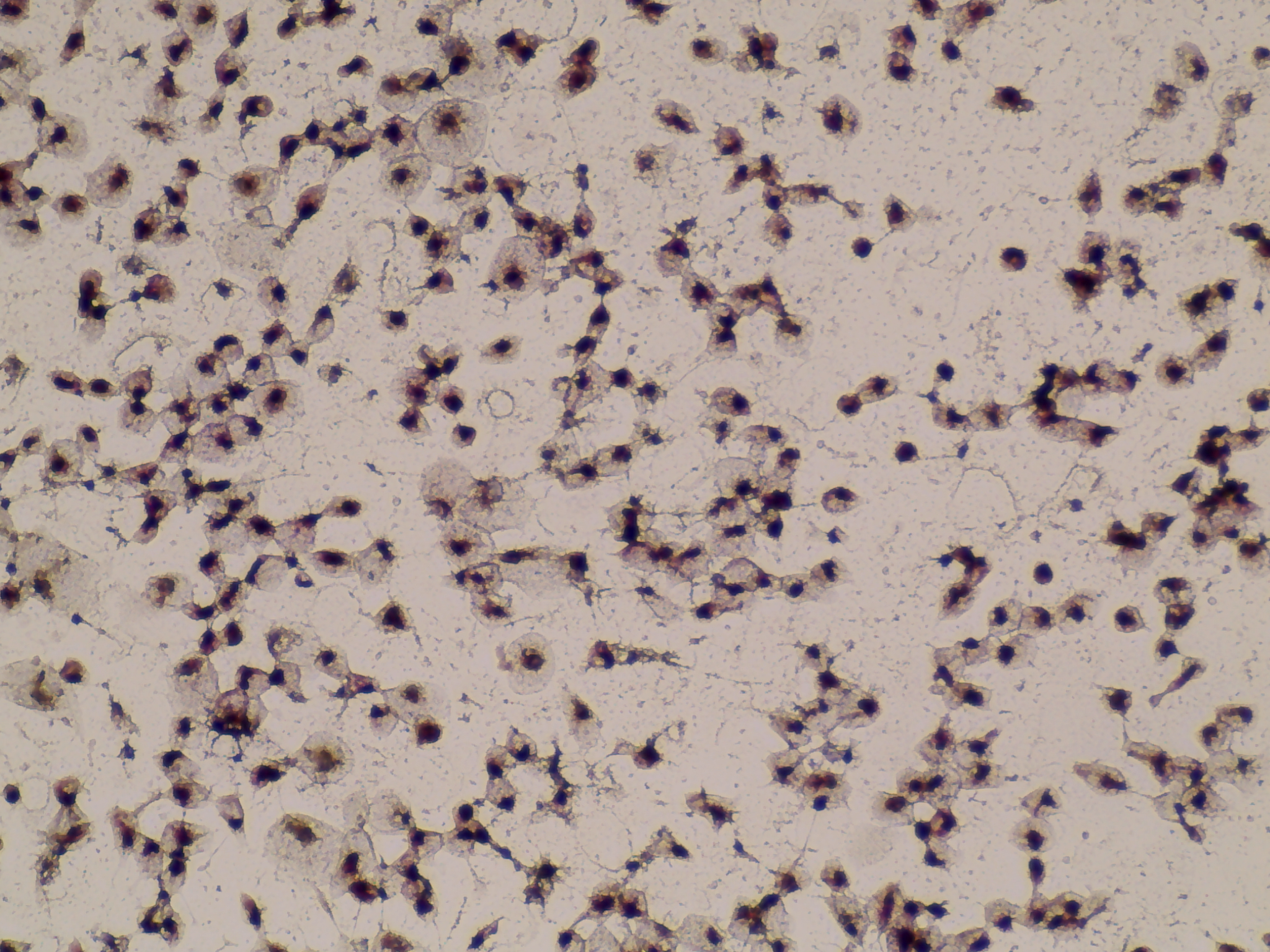

Supplement: Supplementary file 7 — Source Data Fig. 6 [file 44321_2024_35_MOESM7_ESM.zip › Figure 6/6A/Day7 PsA77-M.tif]

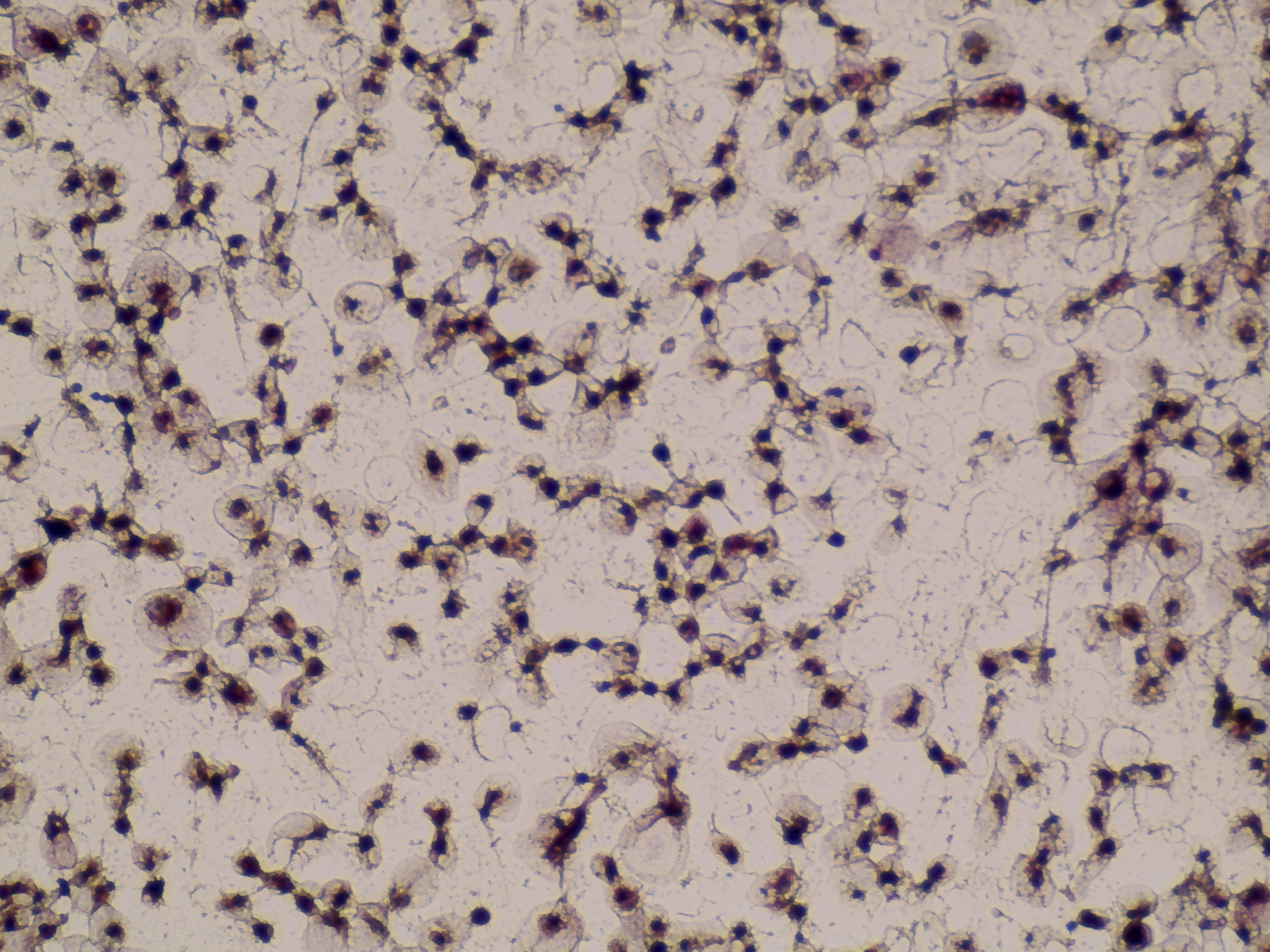

Supplement: Supplementary file 7 — Source Data Fig. 6 [file 44321_2024_35_MOESM7_ESM.zip › Figure 6/6A/Day7 PsA 961-M.tif]

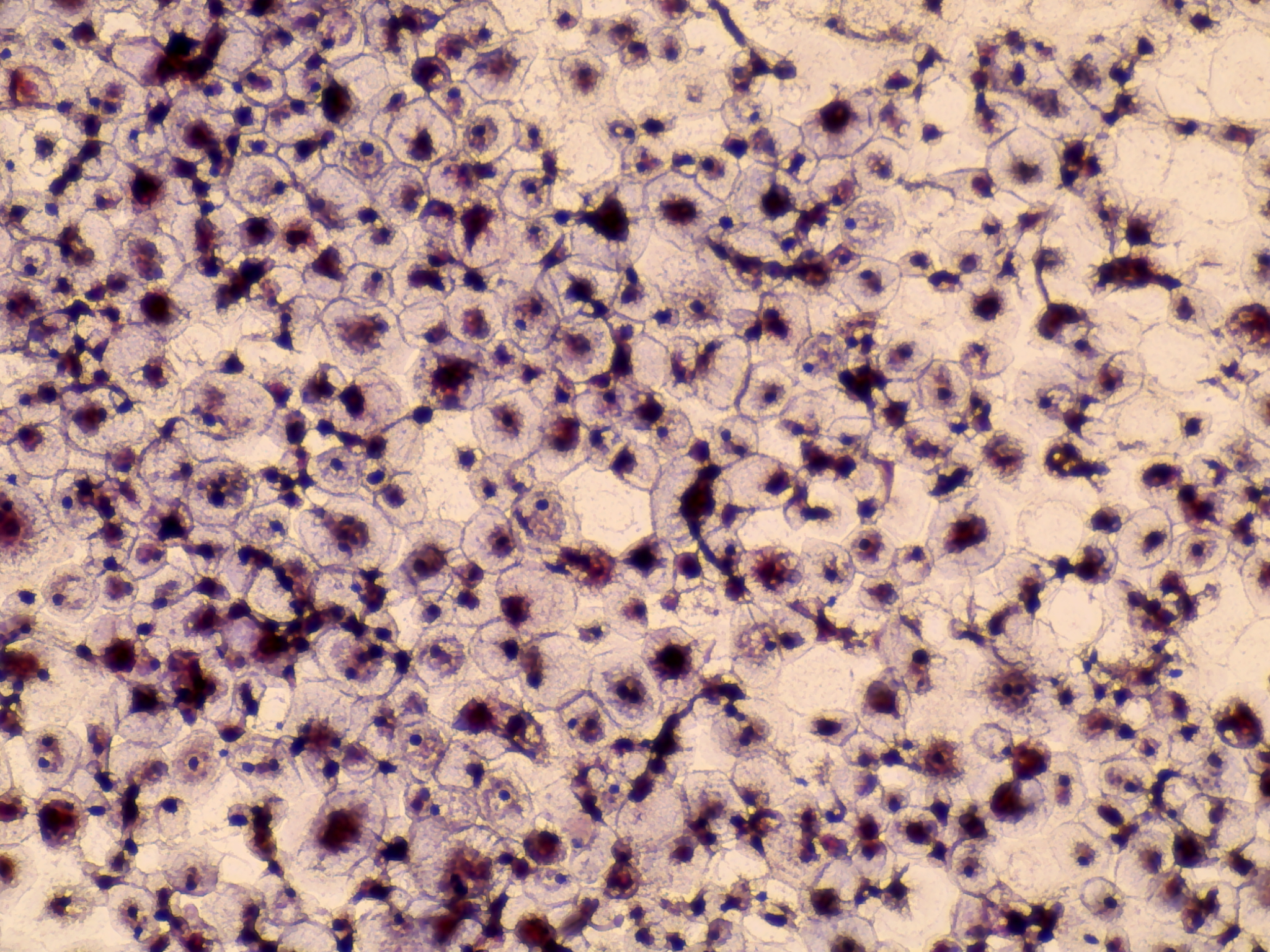

Supplement: Supplementary file 7 — Source Data Fig. 6 [file 44321_2024_35_MOESM7_ESM.zip › Figure 6/6A/Day11 PsA77-M.tif]

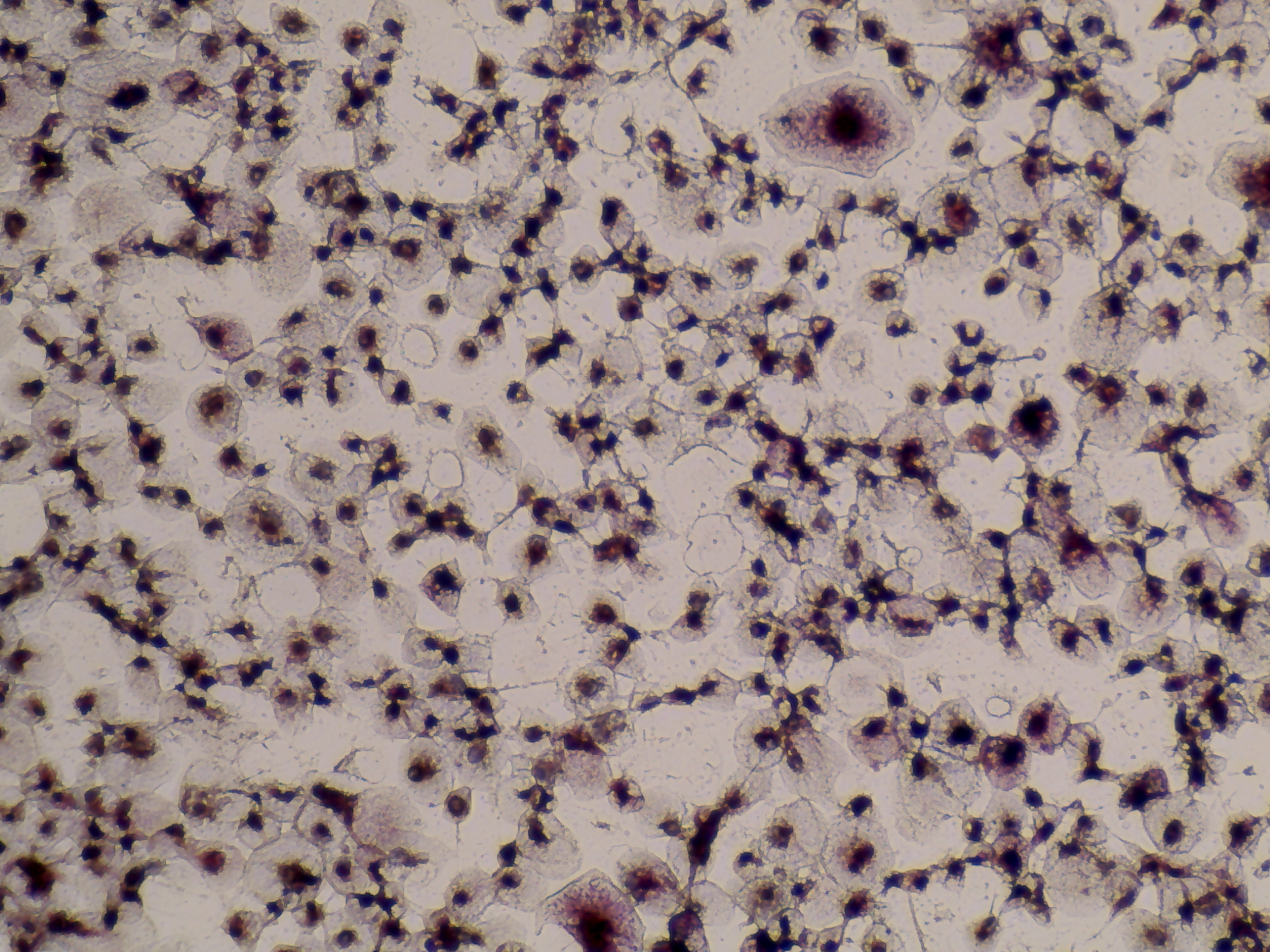

Supplement: Supplementary file 7 — Source Data Fig. 6 [file 44321_2024_35_MOESM7_ESM.zip › Figure 6/6A/Day7 PsA77-M-R.tif]

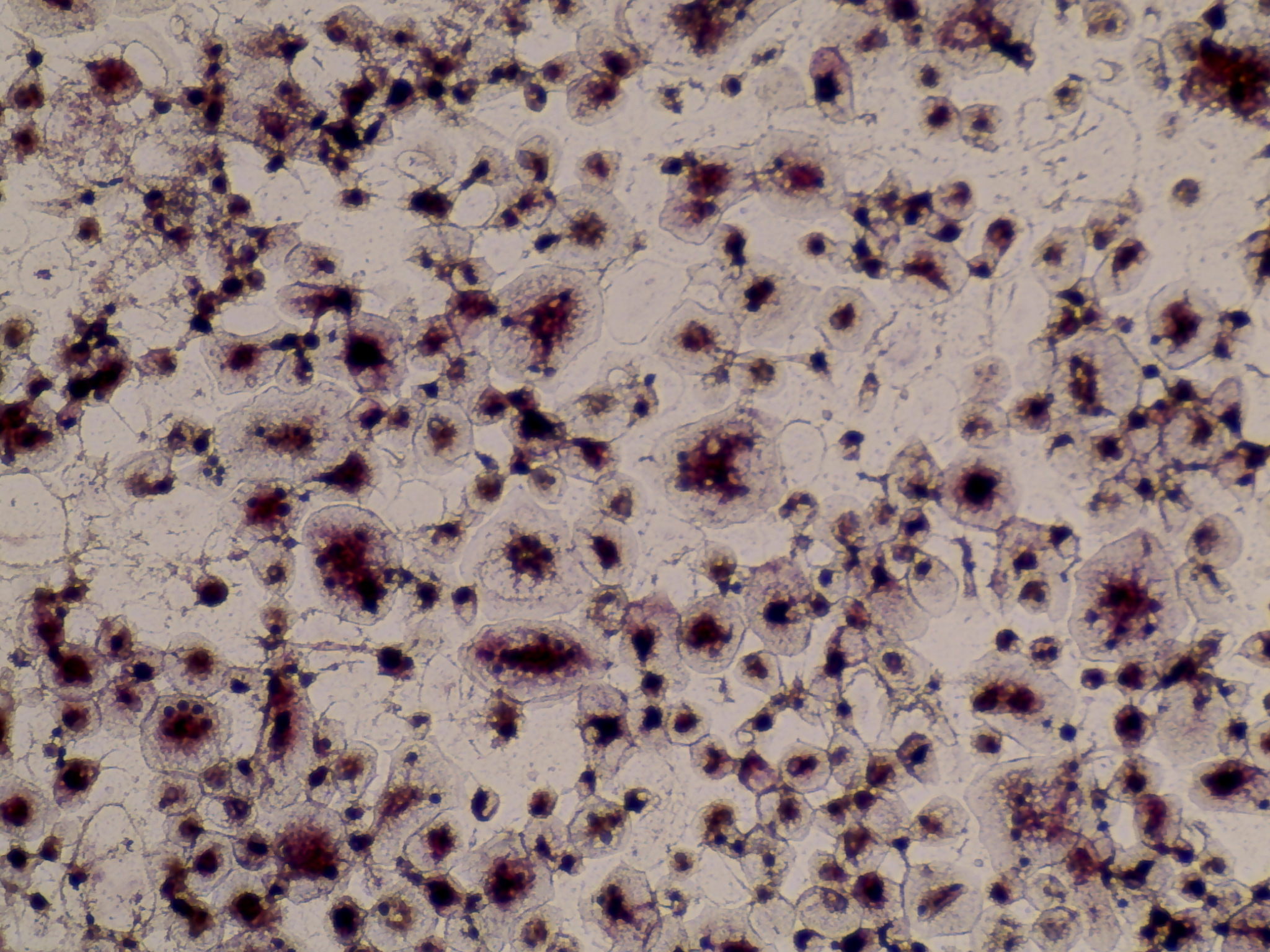

Supplement: Supplementary file 7 — Source Data Fig. 6 [file 44321_2024_35_MOESM7_ESM.zip › Figure 6/6A/Day7 PsA 961-M-R.tif]

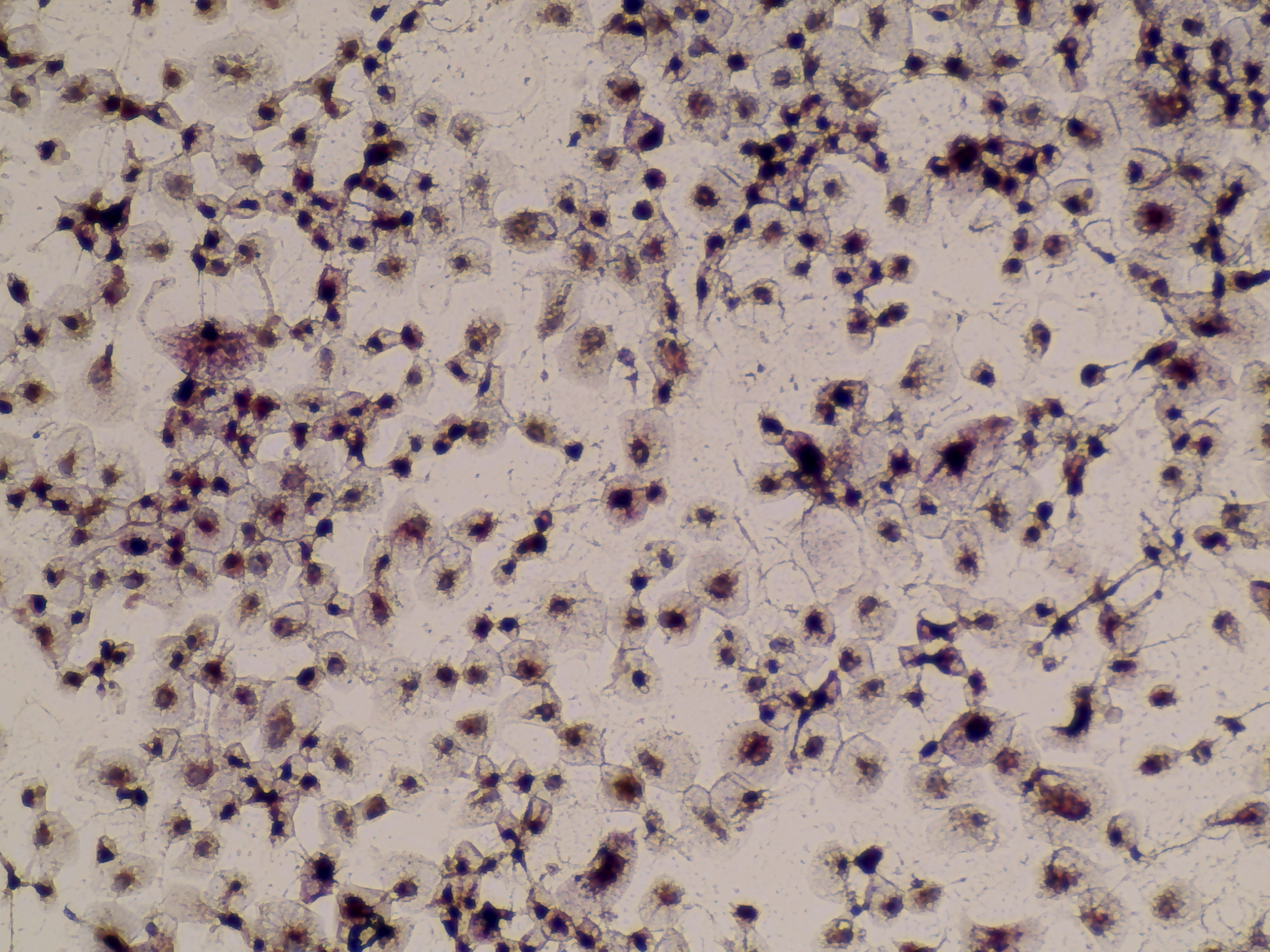

Supplement: Supplementary file 7 — Source Data Fig. 6 [file 44321_2024_35_MOESM7_ESM.zip › Figure 6/6A/Day7 C17-M-R .tif]

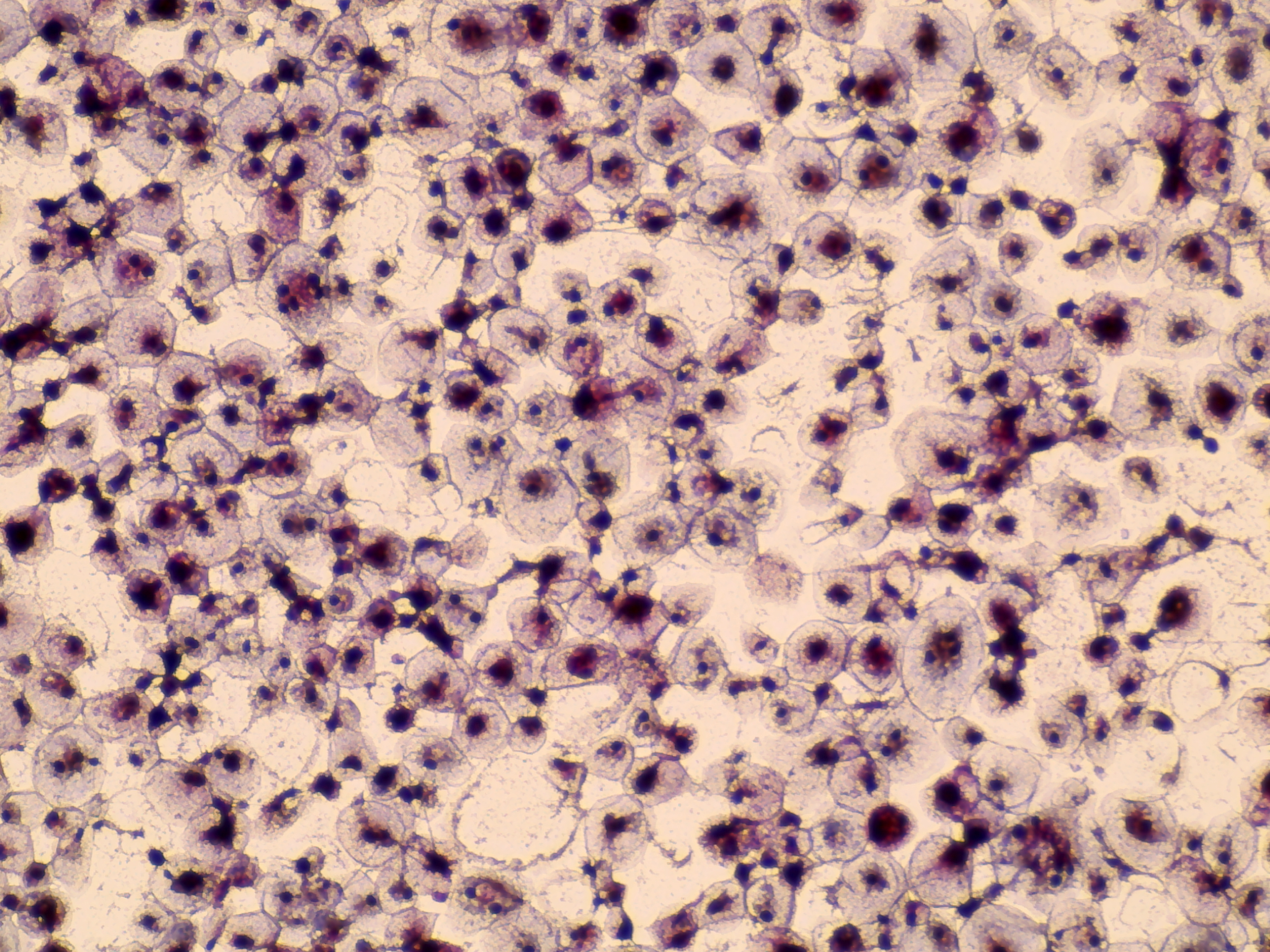

Supplement: Supplementary file 7 — Source Data Fig. 6 [file 44321_2024_35_MOESM7_ESM.zip › Figure 6/6A/Day11 PsA961-M.tif]

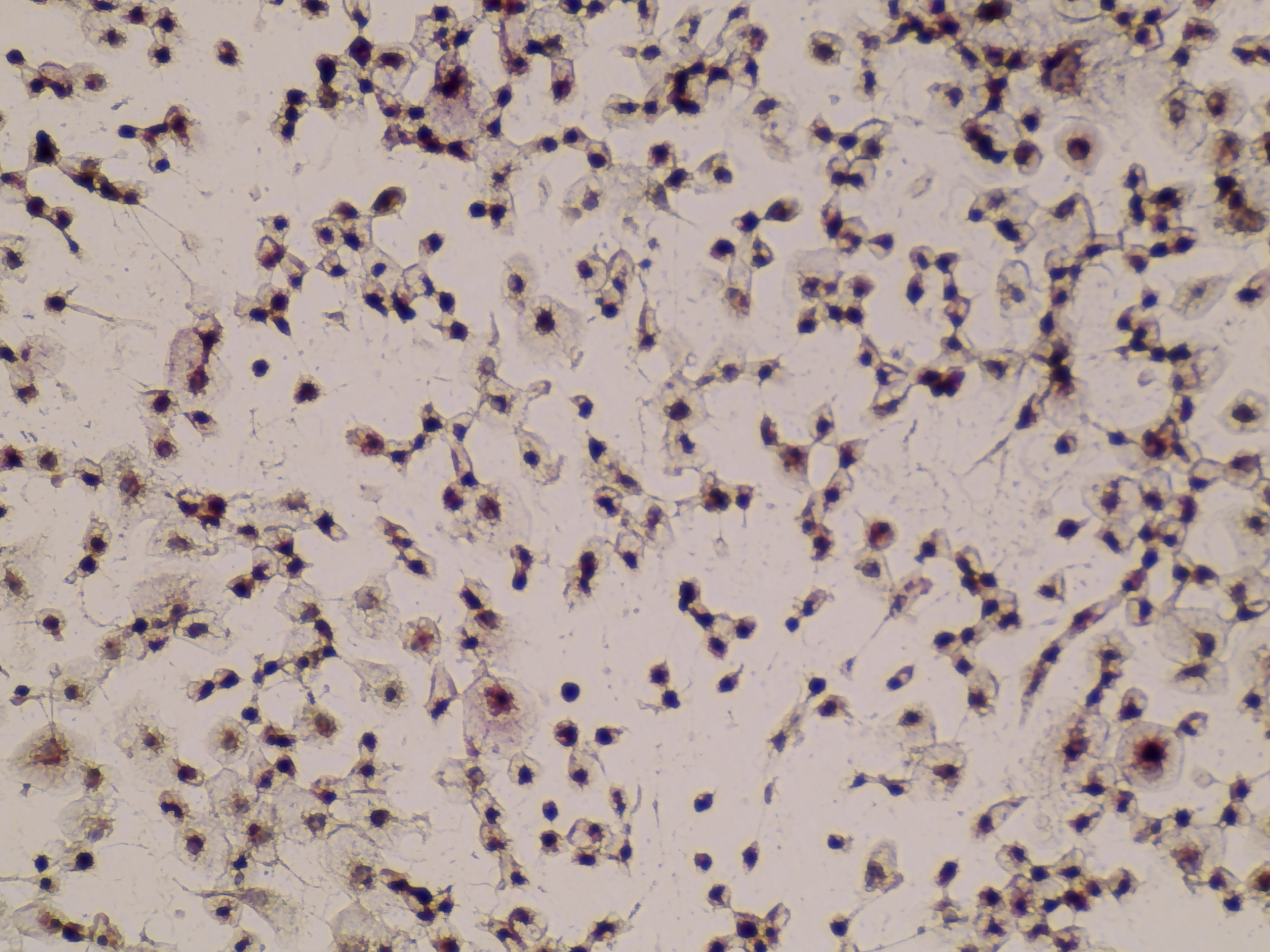

Supplement: Supplementary file 7 — Source Data Fig. 6 [file 44321_2024_35_MOESM7_ESM.zip › Figure 6/6A/Day7 C17-M.tif]

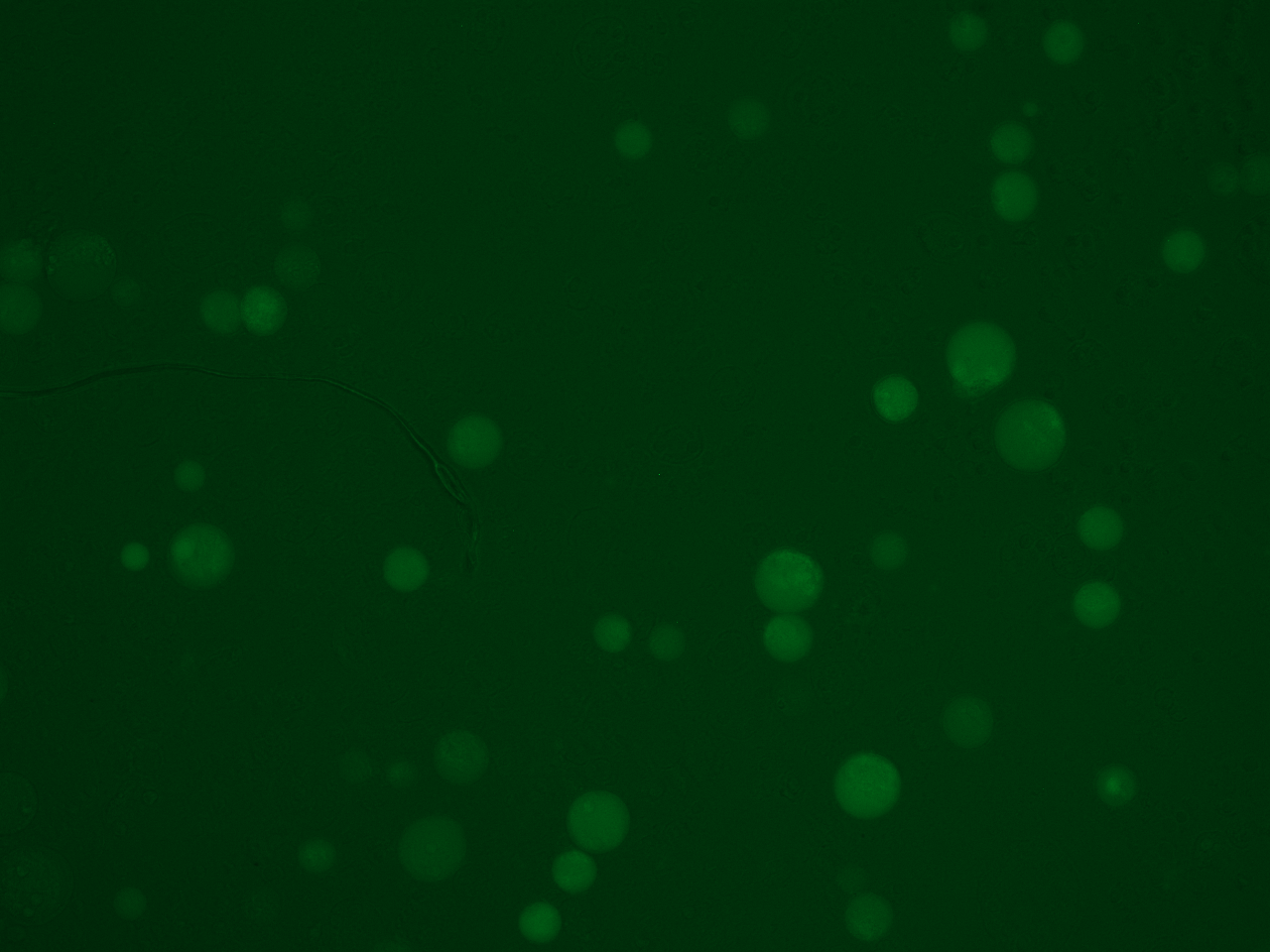

Supplement: Supplementary file 7 — Source Data Fig. 6 [file 44321_2024_35_MOESM7_ESM.zip › Figure 6/6B/Day 9 PsA77+M+R.tif]

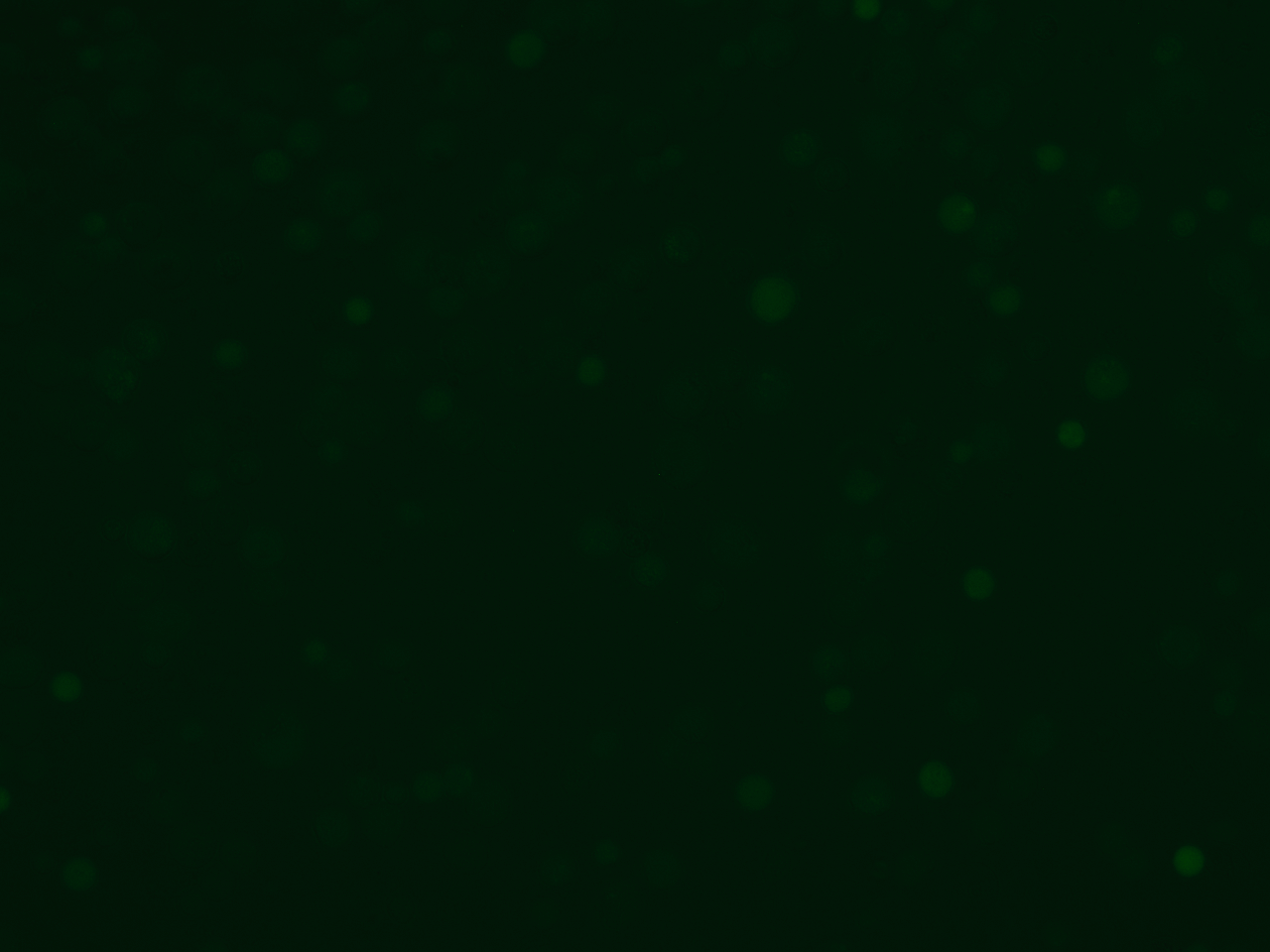

Supplement: Supplementary file 7 — Source Data Fig. 6 [file 44321_2024_35_MOESM7_ESM.zip › Figure 6/6B/Day 7 PsA961+ M.tif]

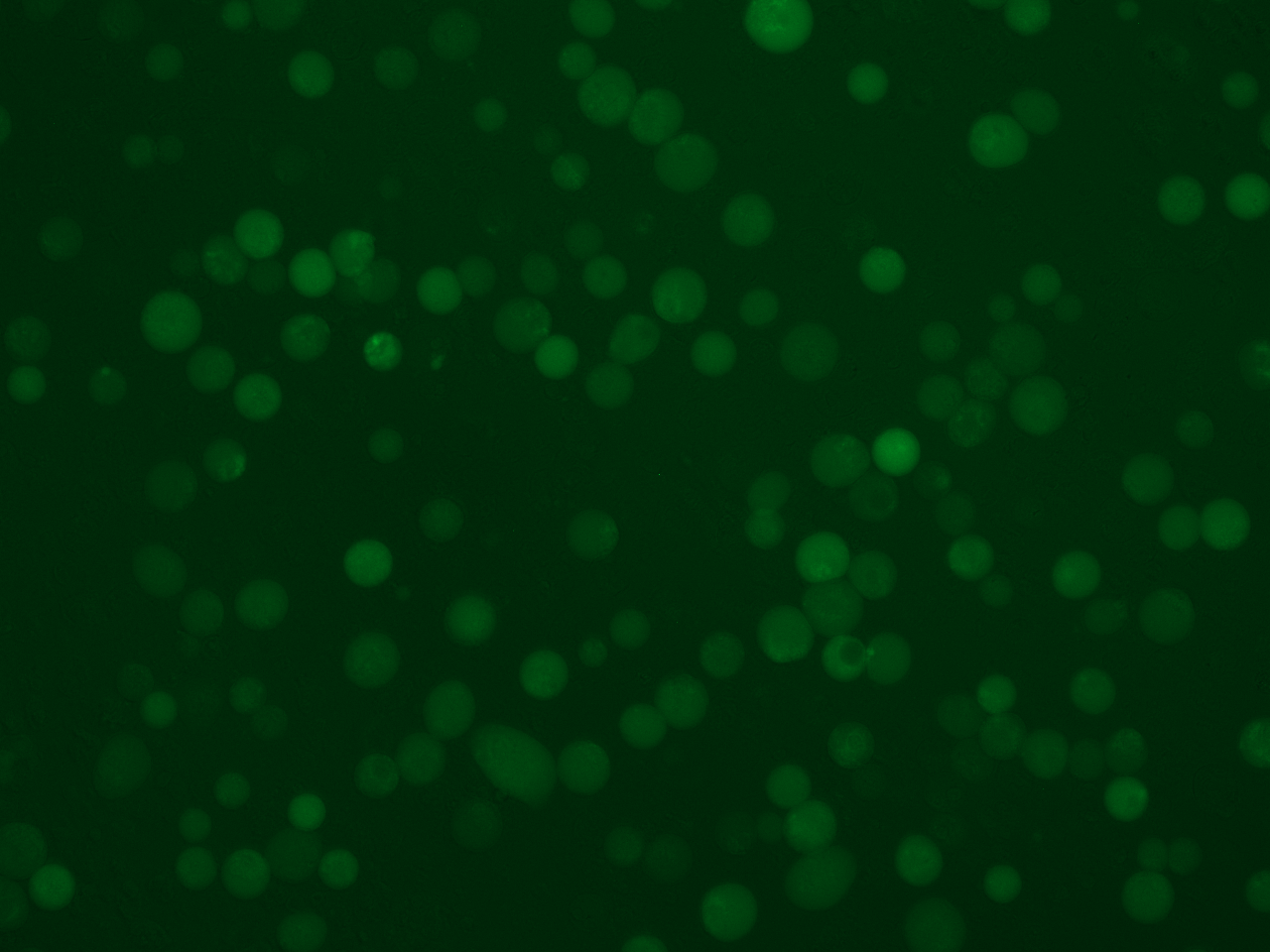

Supplement: Supplementary file 7 — Source Data Fig. 6 [file 44321_2024_35_MOESM7_ESM.zip › Figure 6/6B/Day 9 PsA961+M.tif]

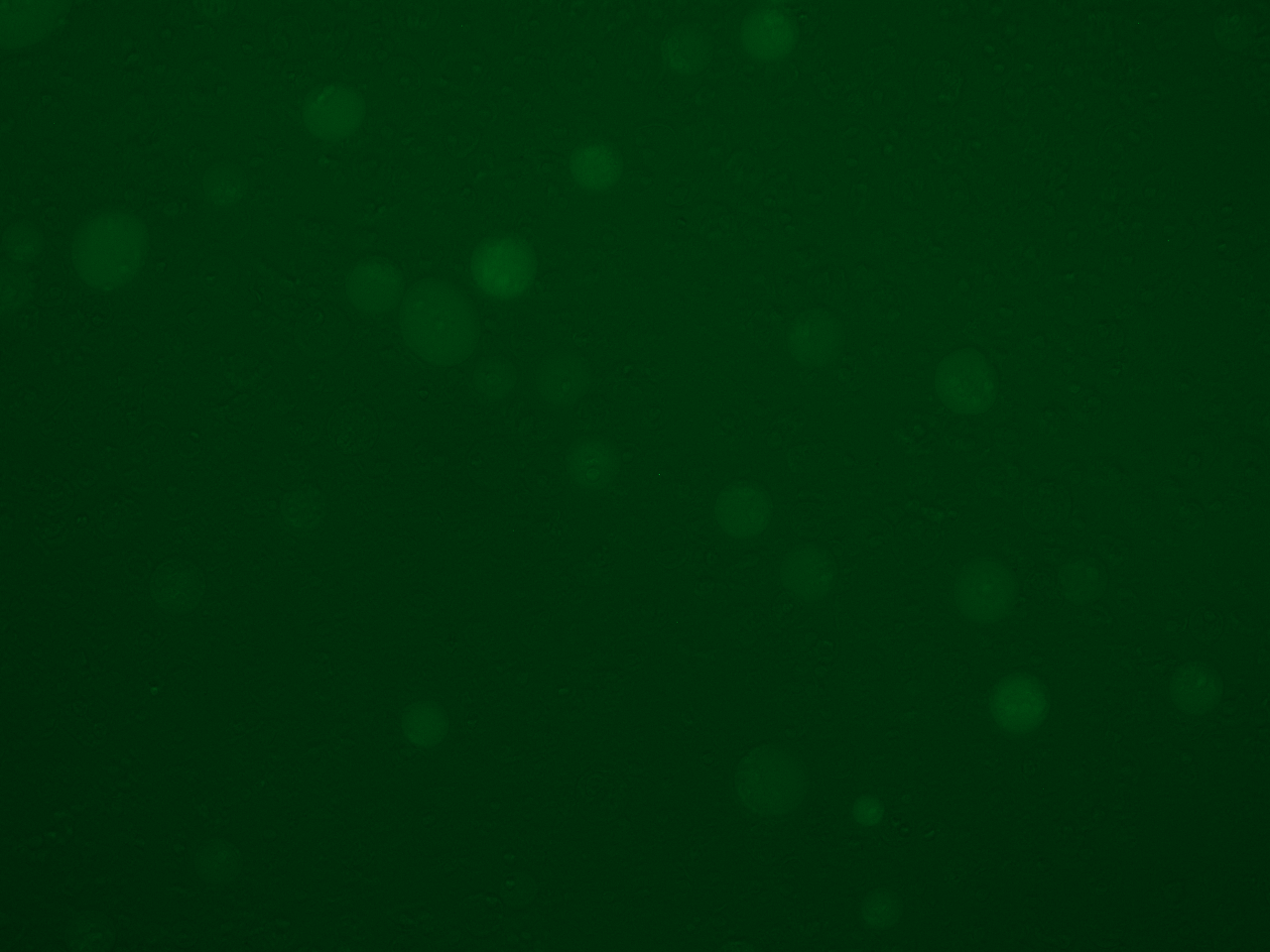

Supplement: Supplementary file 7 — Source Data Fig. 6 [file 44321_2024_35_MOESM7_ESM.zip › Figure 6/6B/Day 11 PsA961+M.tif]

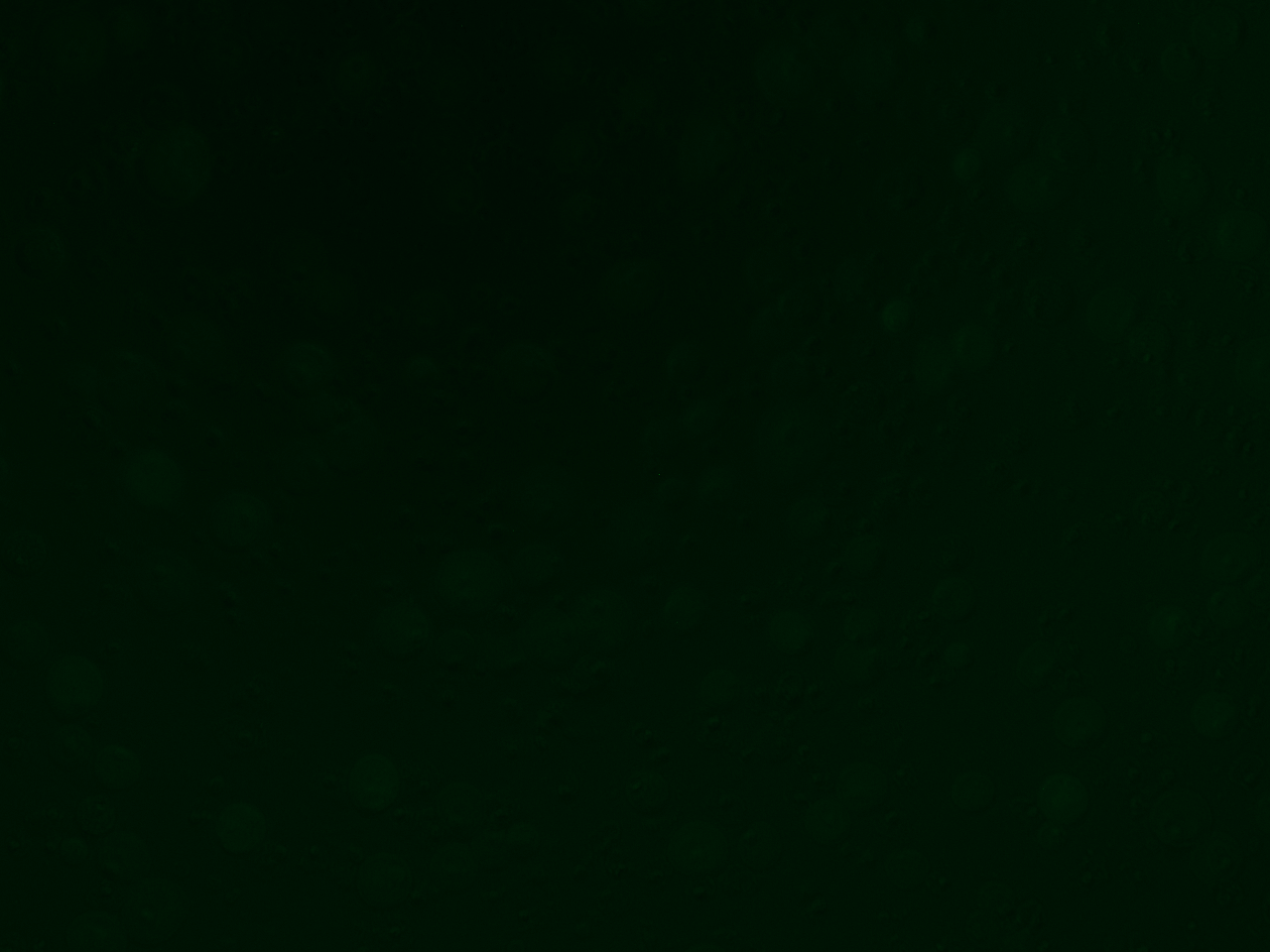

Supplement: Supplementary file 7 — Source Data Fig. 6 [file 44321_2024_35_MOESM7_ESM.zip › Figure 6/6B/Day 7 PsA77-M.tif]

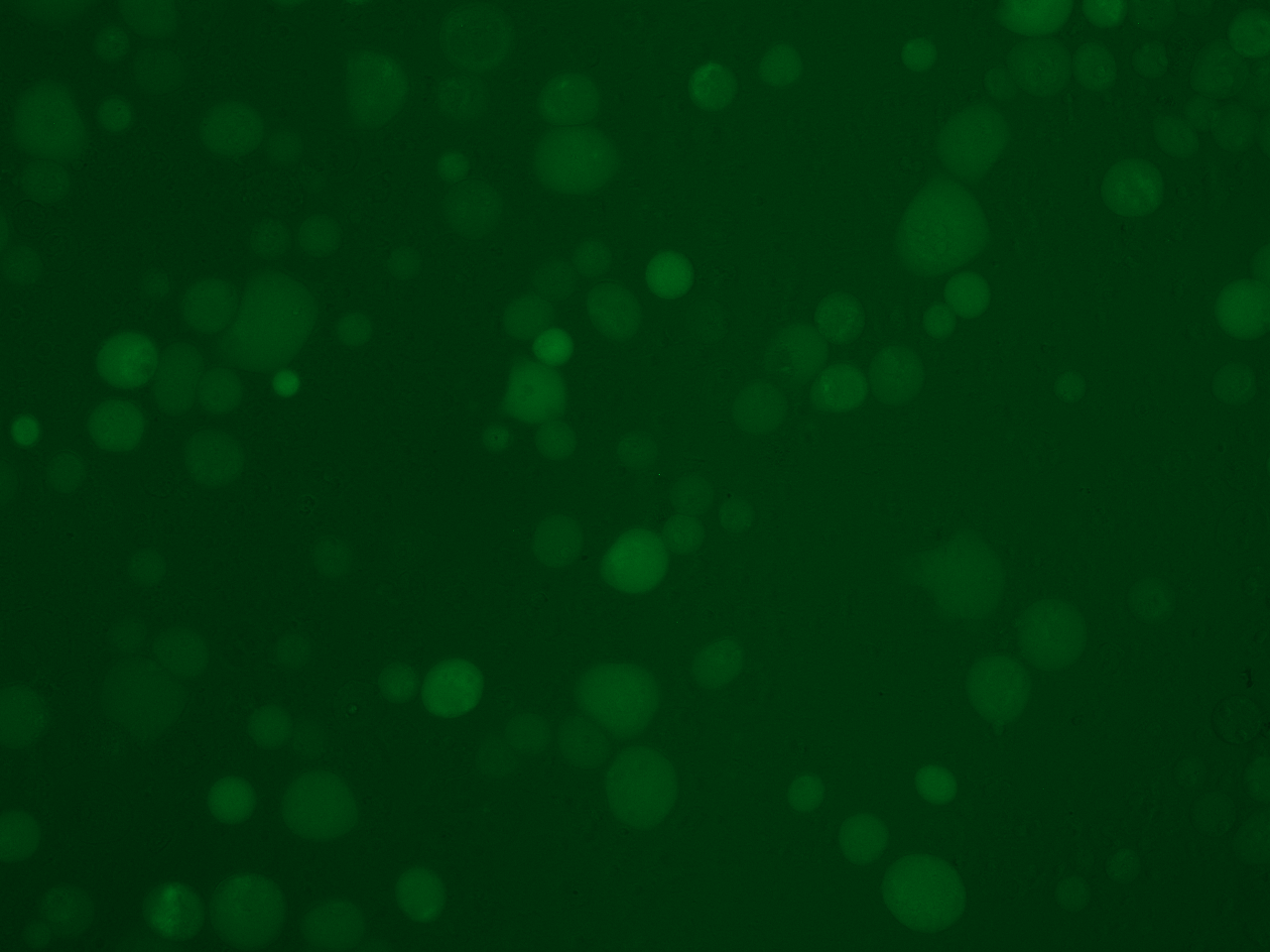

Supplement: Supplementary file 7 — Source Data Fig. 6 [file 44321_2024_35_MOESM7_ESM.zip › Figure 6/6B/Day 11 PsA77+M+R.tif]

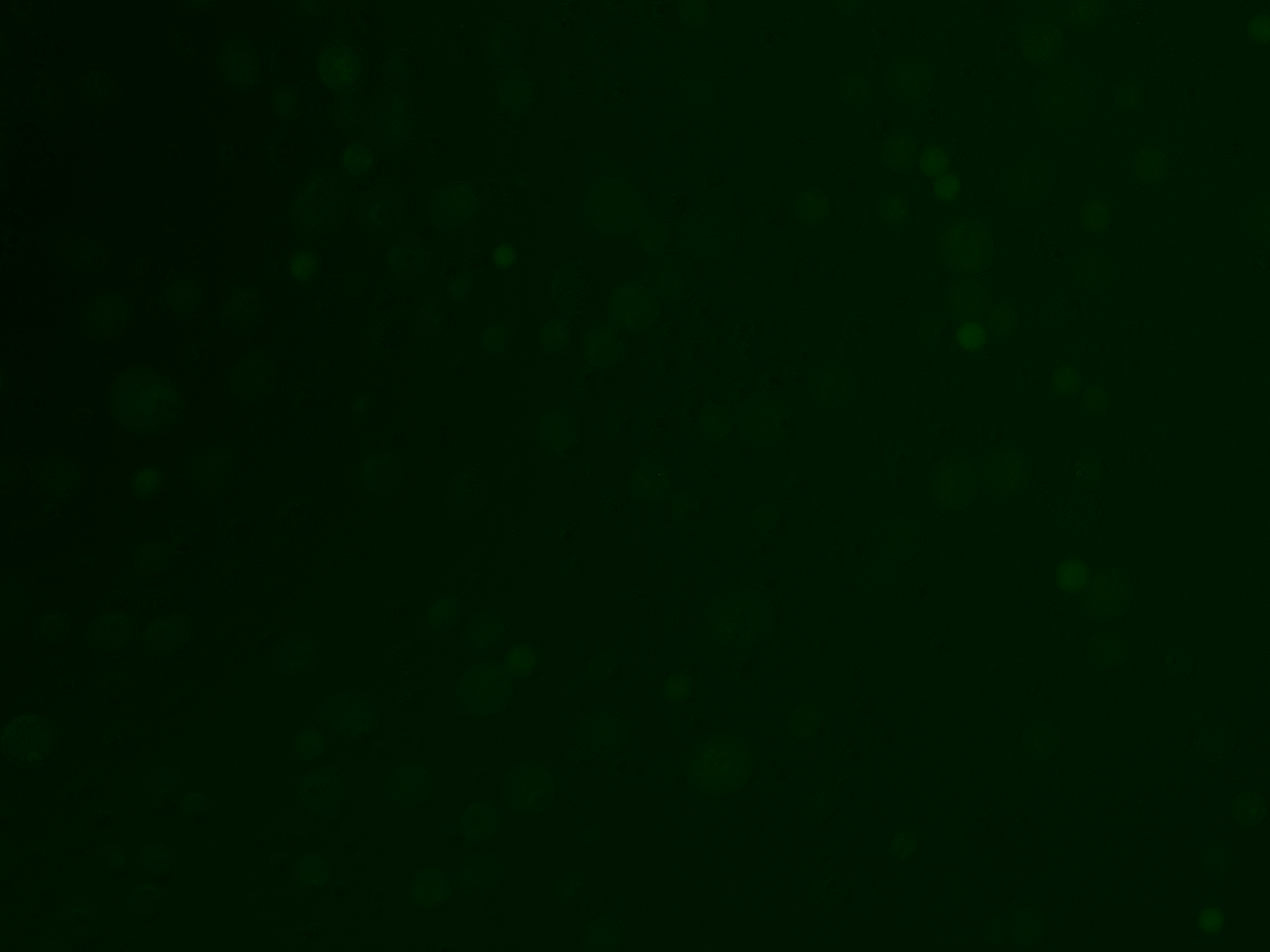

Supplement: Supplementary file 7 — Source Data Fig. 6 [file 44321_2024_35_MOESM7_ESM.zip › Figure 6/6B/Day 7 PsA77+M+R.tif]

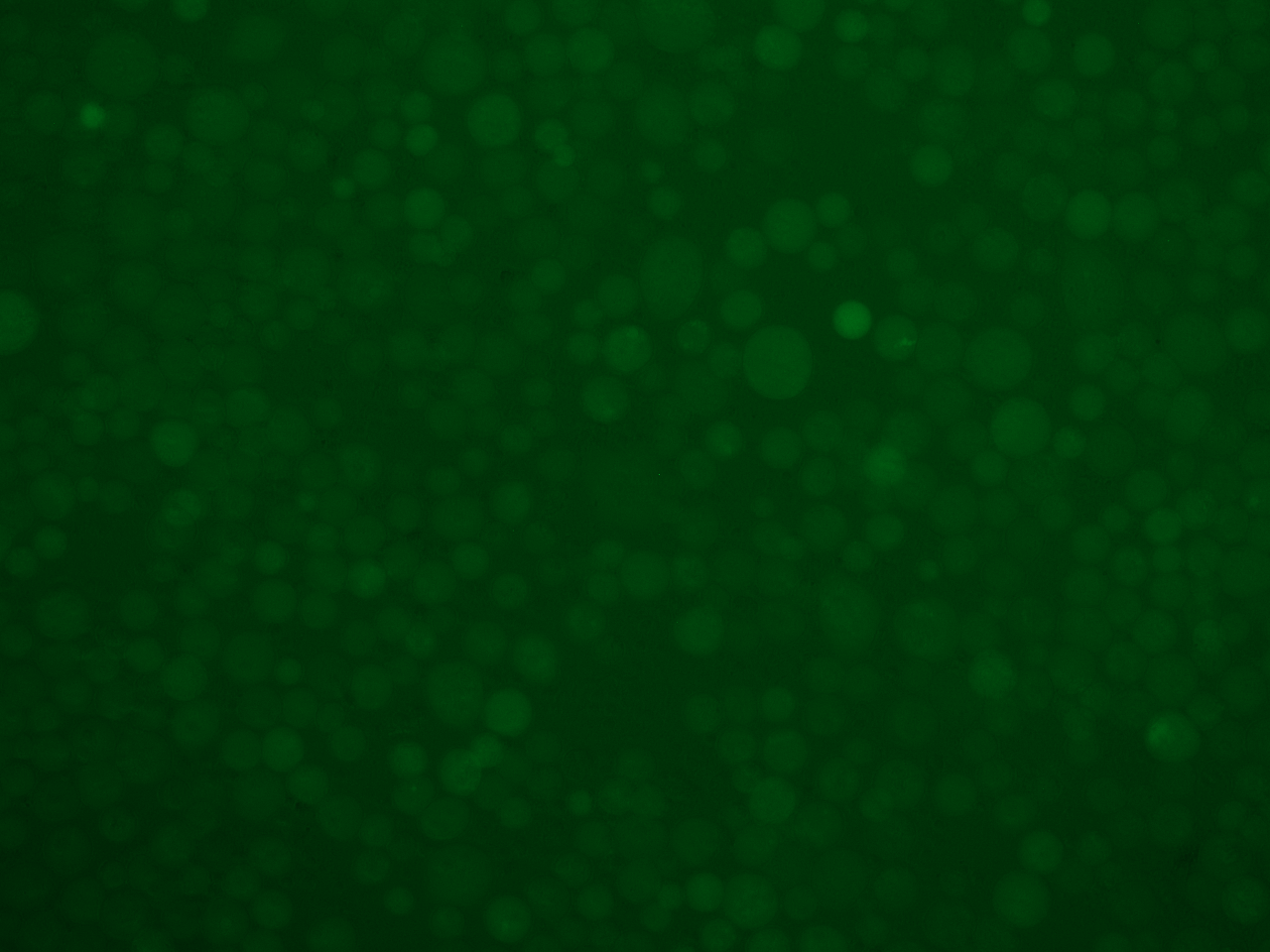

Supplement: Supplementary file 7 — Source Data Fig. 6 [file 44321_2024_35_MOESM7_ESM.zip › Figure 6/6B/Day 9 control+M+R.tif]

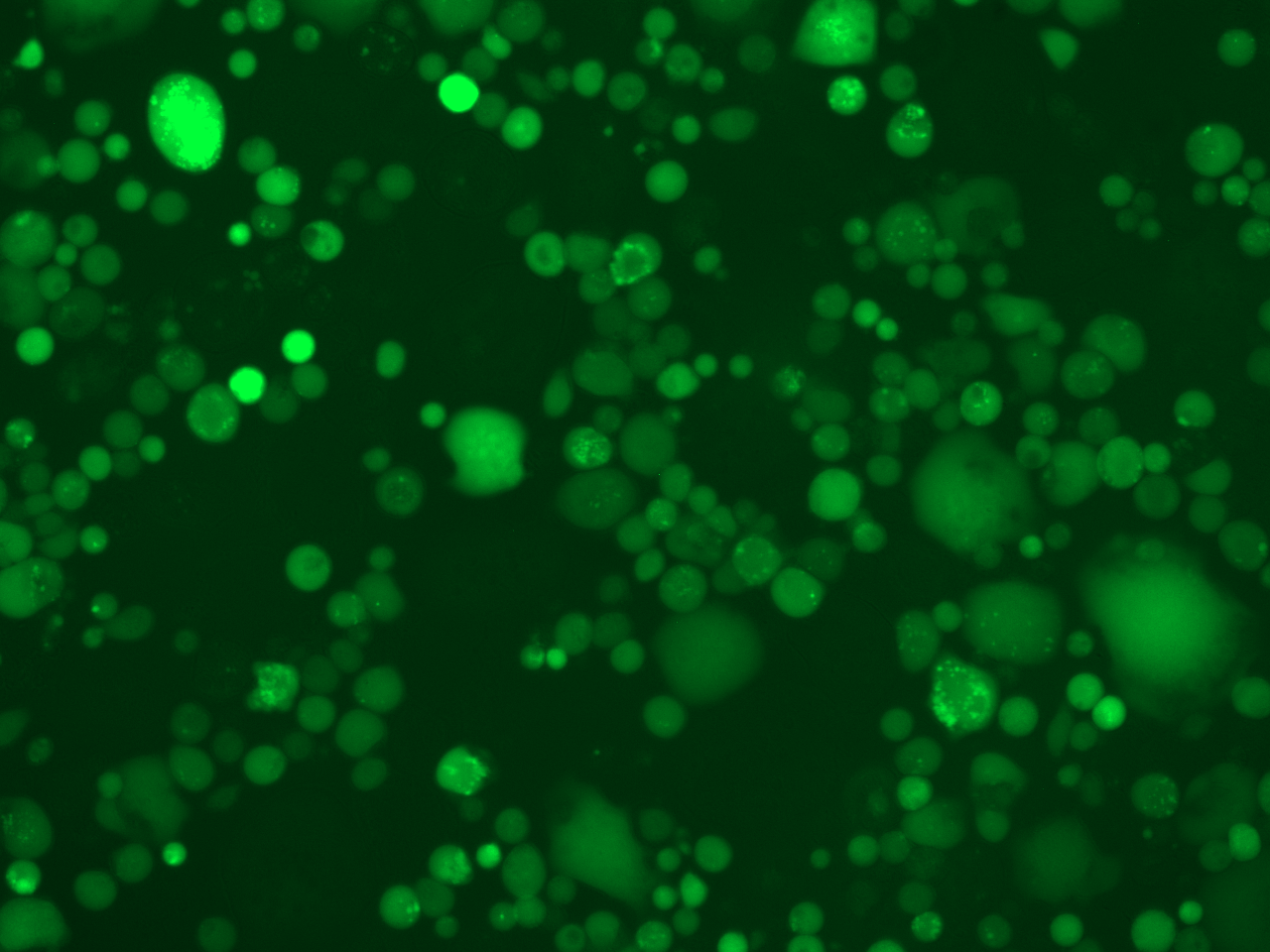

Supplement: Supplementary file 7 — Source Data Fig. 6 [file 44321_2024_35_MOESM7_ESM.zip › Figure 6/6B/Day 11 PsA961+M+R.tif]

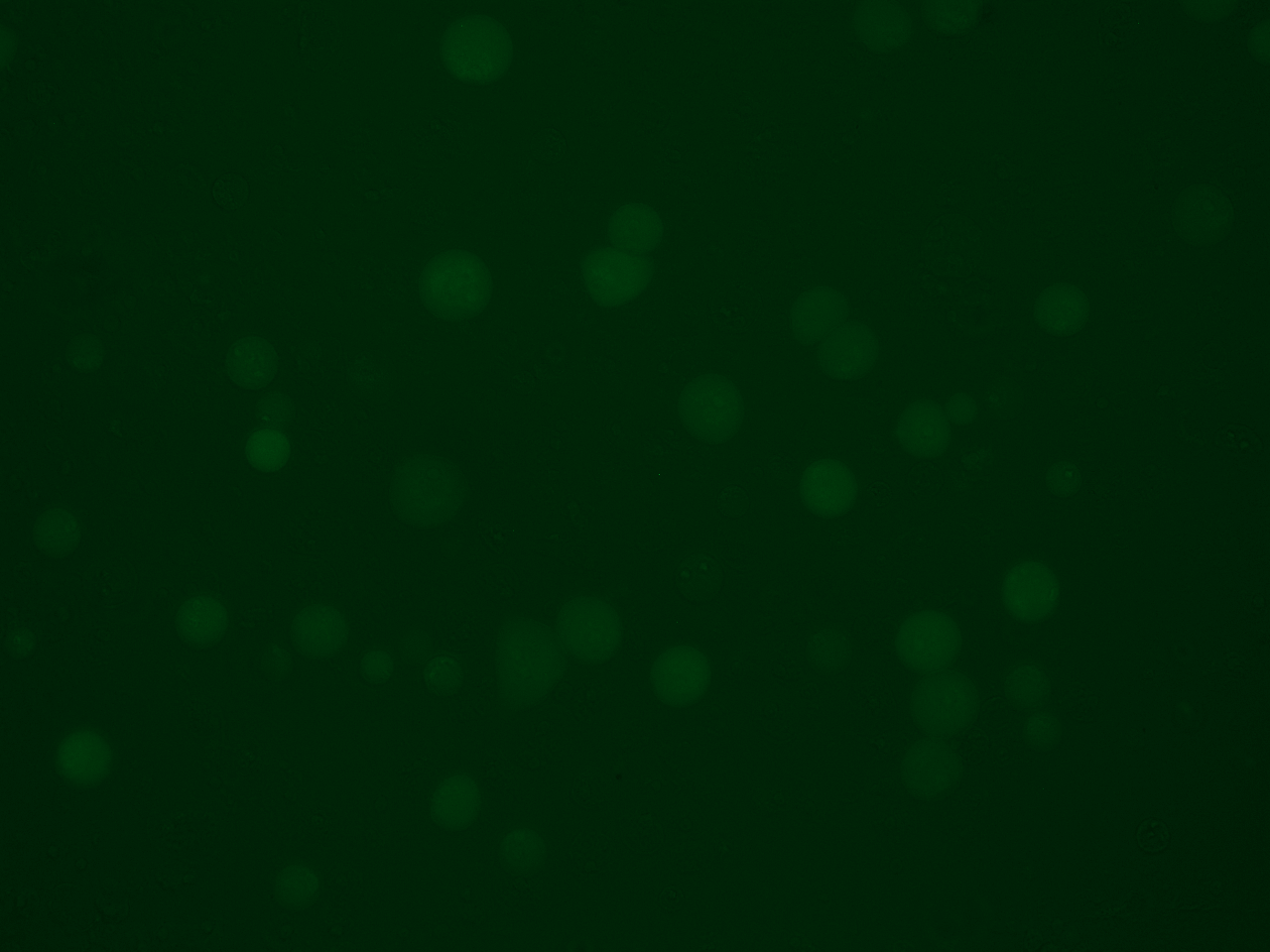

Supplement: Supplementary file 7 — Source Data Fig. 6 [file 44321_2024_35_MOESM7_ESM.zip › Figure 6/6B/Day 11 control+M.tif]

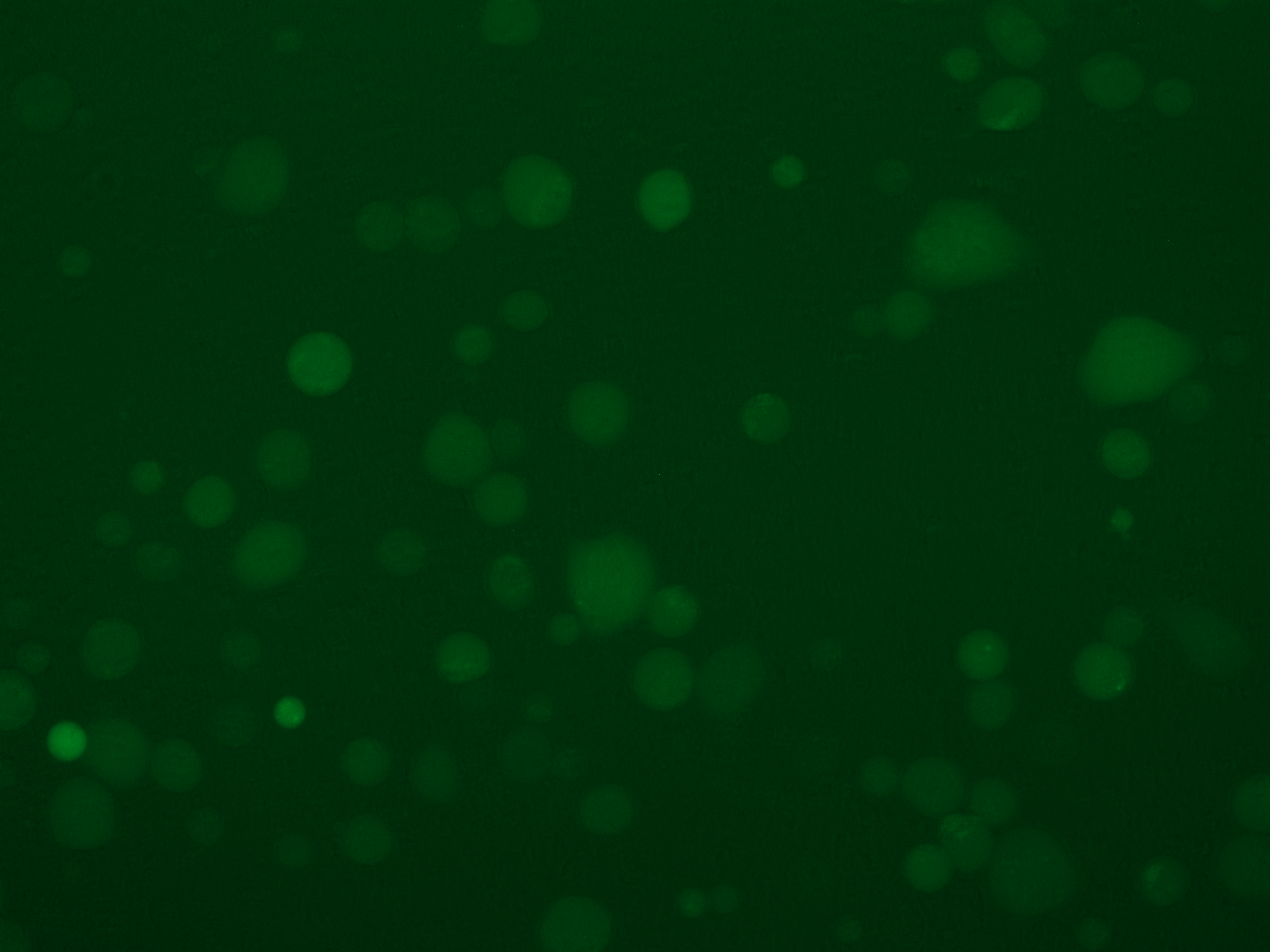

Supplement: Supplementary file 7 — Source Data Fig. 6 [file 44321_2024_35_MOESM7_ESM.zip › Figure 6/6B/Day 11 PsA77+M.tif]

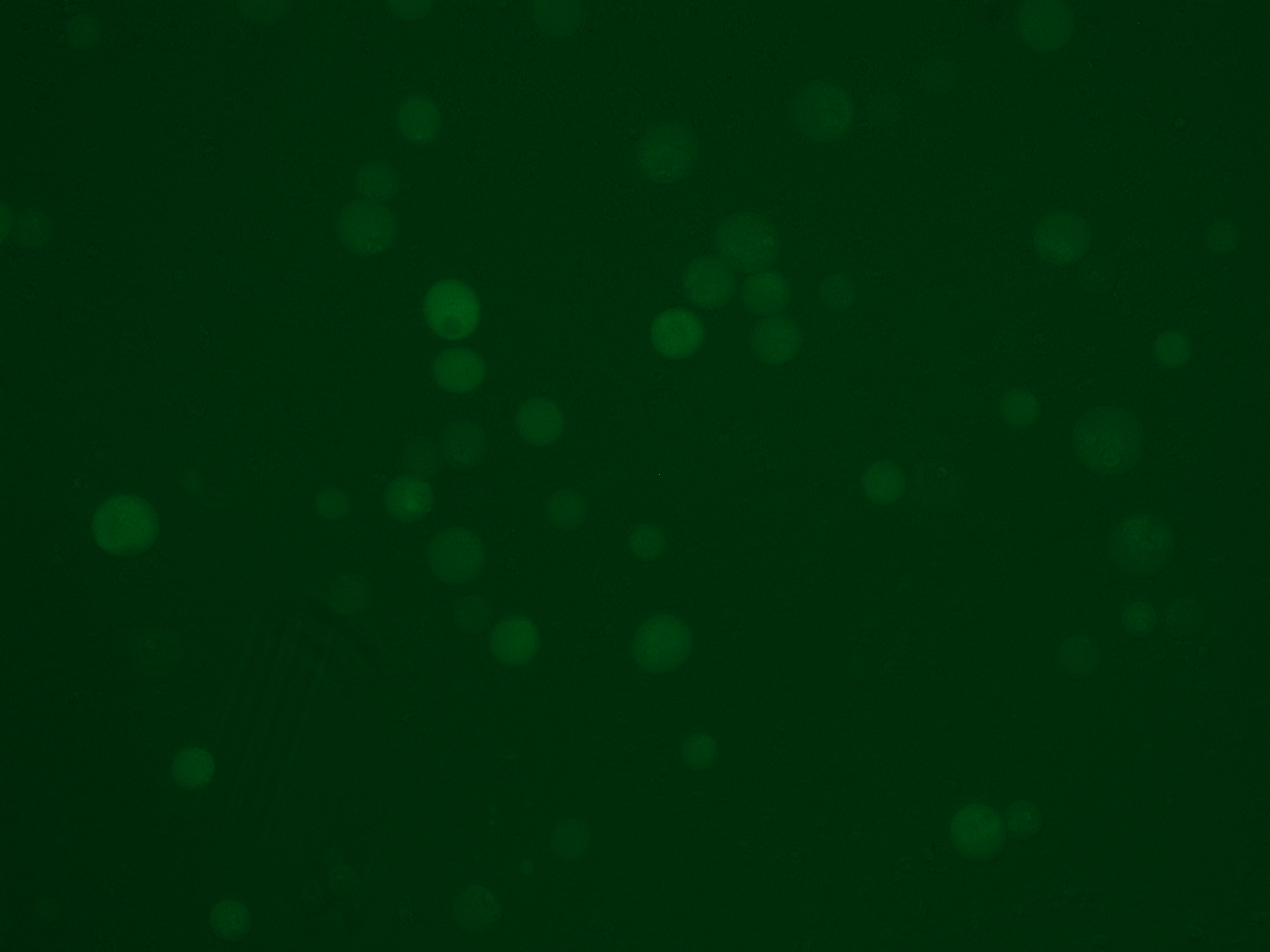

Supplement: Supplementary file 7 — Source Data Fig. 6 [file 44321_2024_35_MOESM7_ESM.zip › Figure 6/6B/Day 9 PsA77+M.tif]

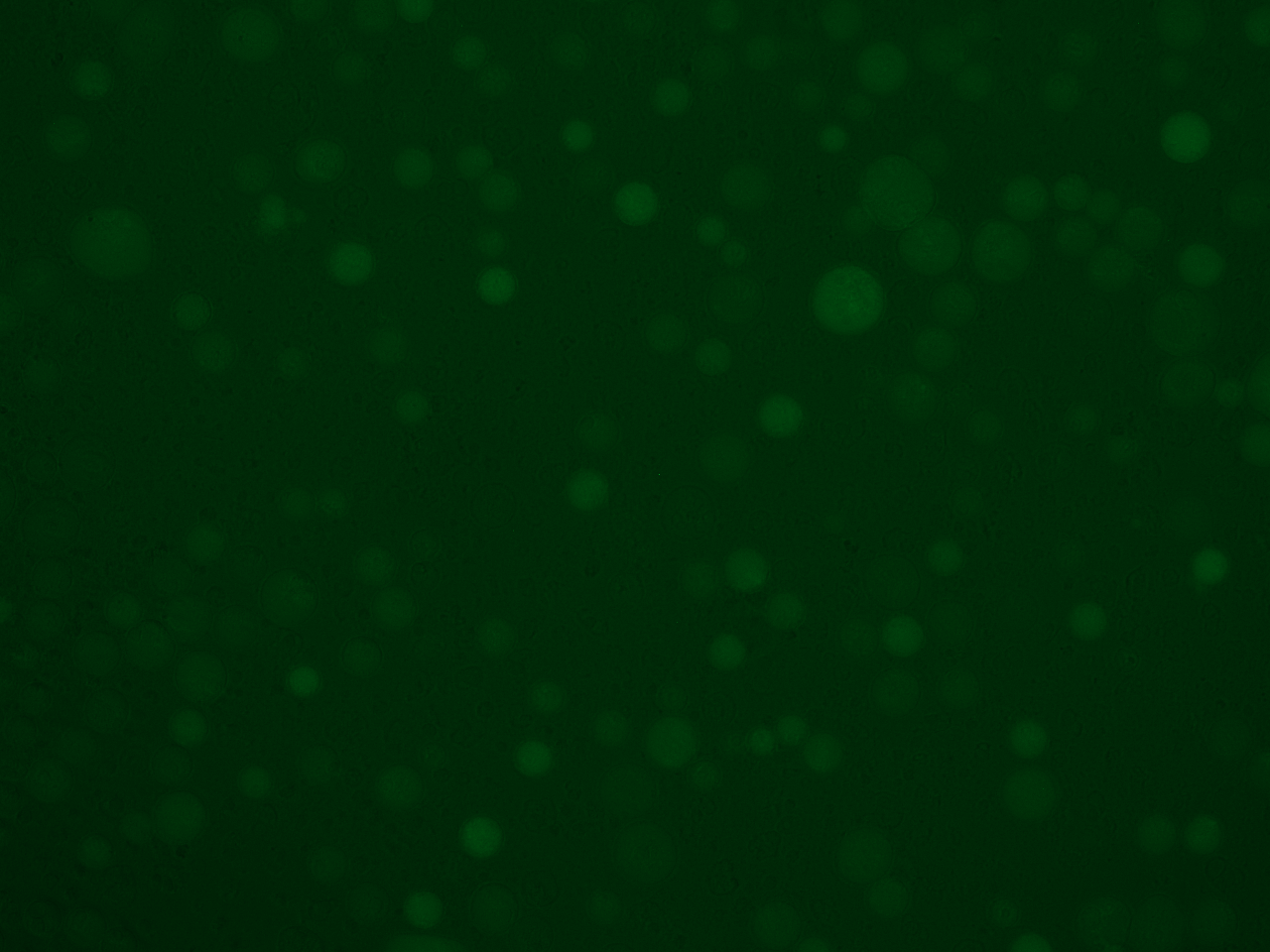

Supplement: Supplementary file 7 — Source Data Fig. 6 [file 44321_2024_35_MOESM7_ESM.zip › Figure 6/6B/Day 9 control+M.tif]

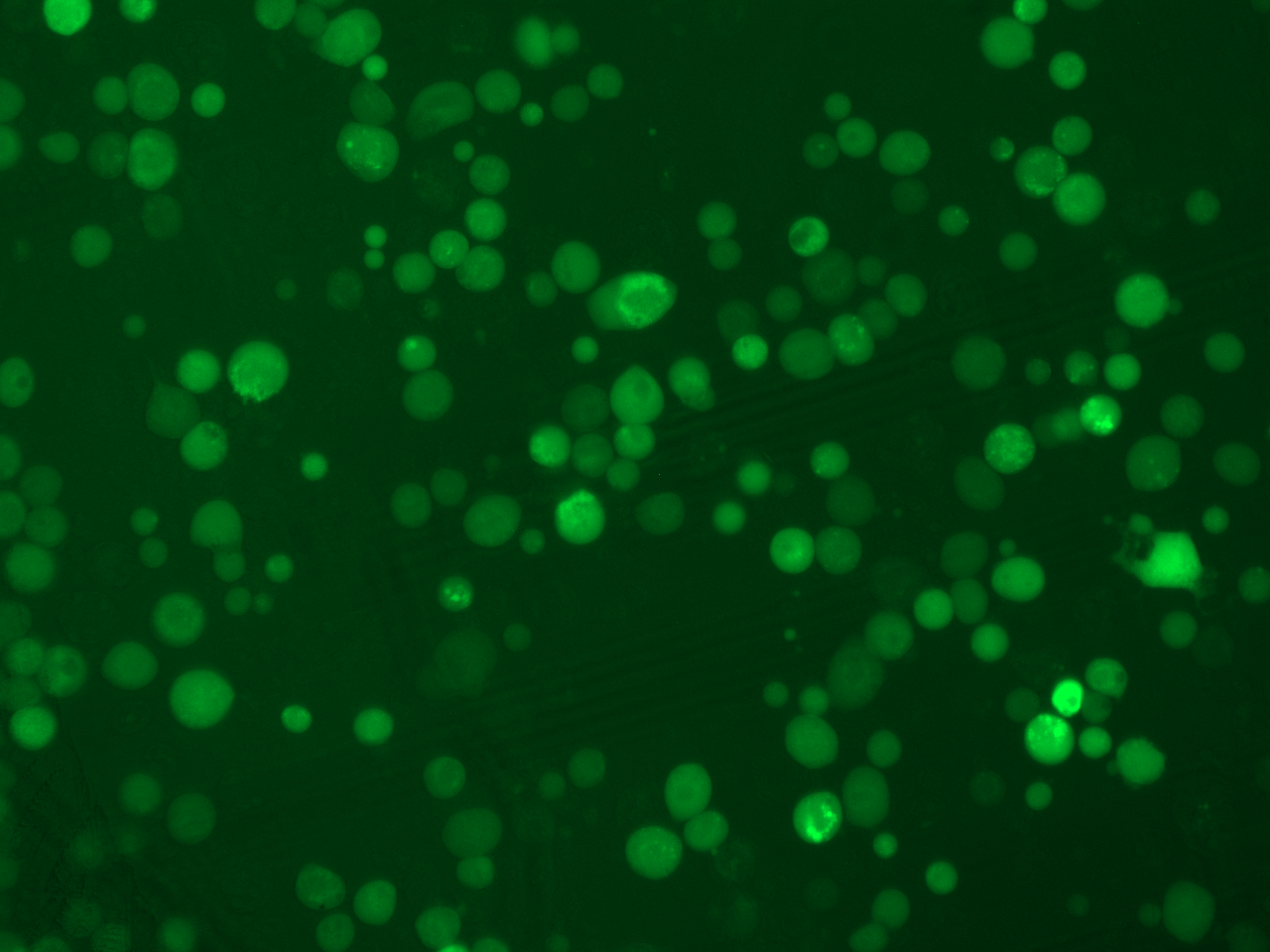

Supplement: Supplementary file 7 — Source Data Fig. 6 [file 44321_2024_35_MOESM7_ESM.zip › Figure 6/6B/Day 9 PsA961+M+R.tif]

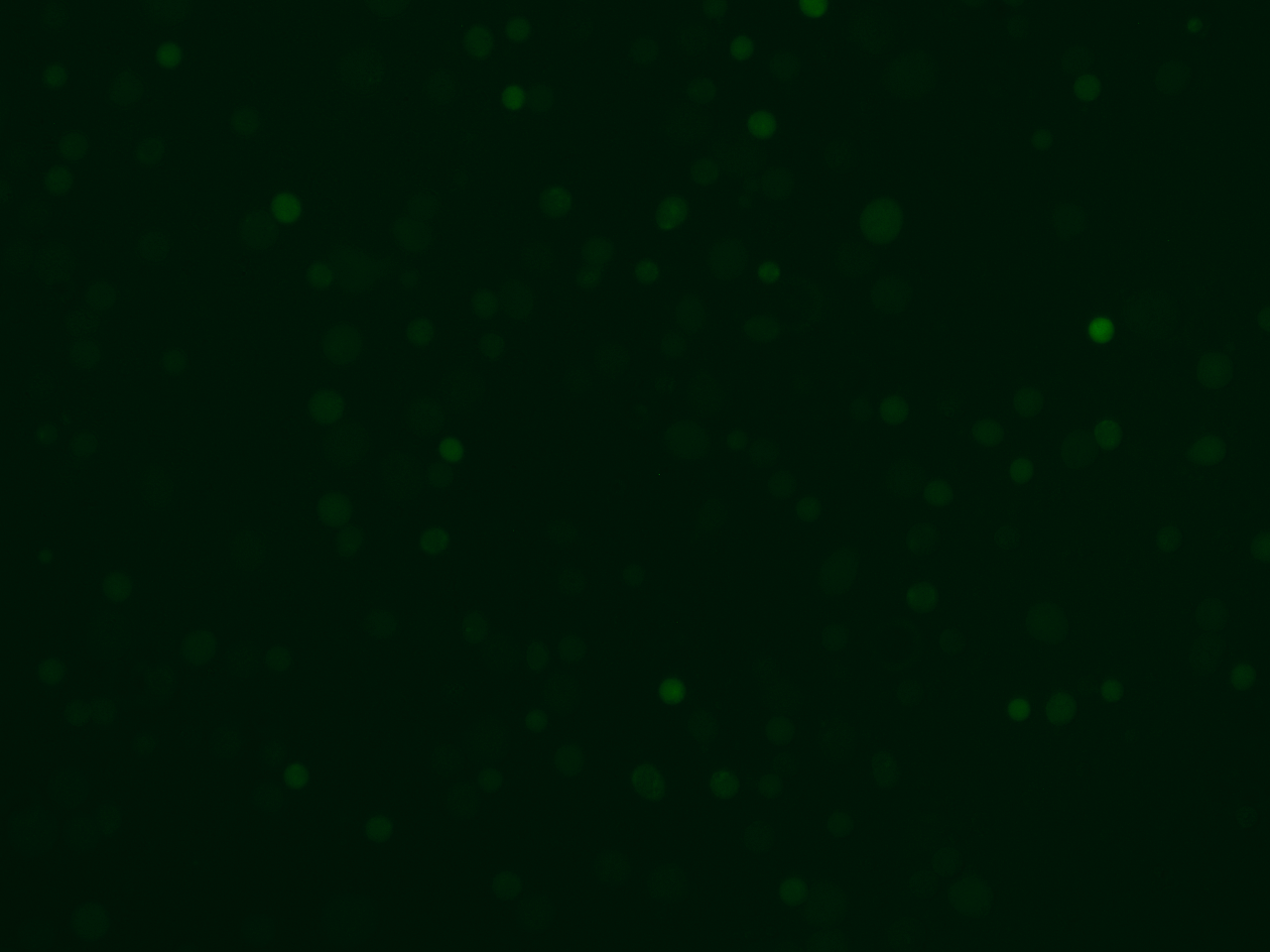

Supplement: Supplementary file 7 — Source Data Fig. 6 [file 44321_2024_35_MOESM7_ESM.zip › Figure 6/6B/Day 7 PsA961+ M+R.tif]

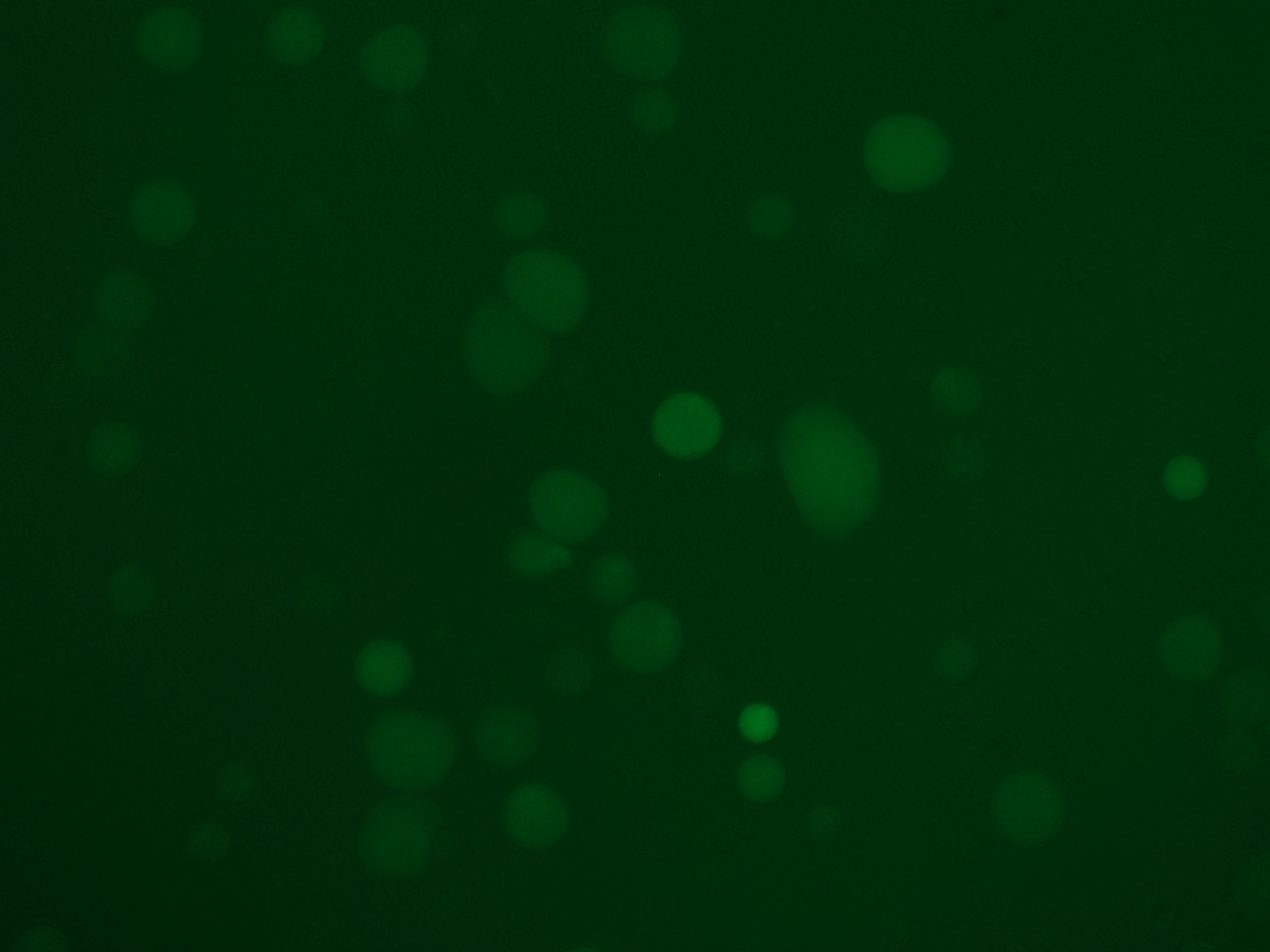

Supplement: Supplementary file 7 — Source Data Fig. 6 [file 44321_2024_35_MOESM7_ESM.zip › Figure 6/6B/Day 11 ccontrol+M+R.tif]

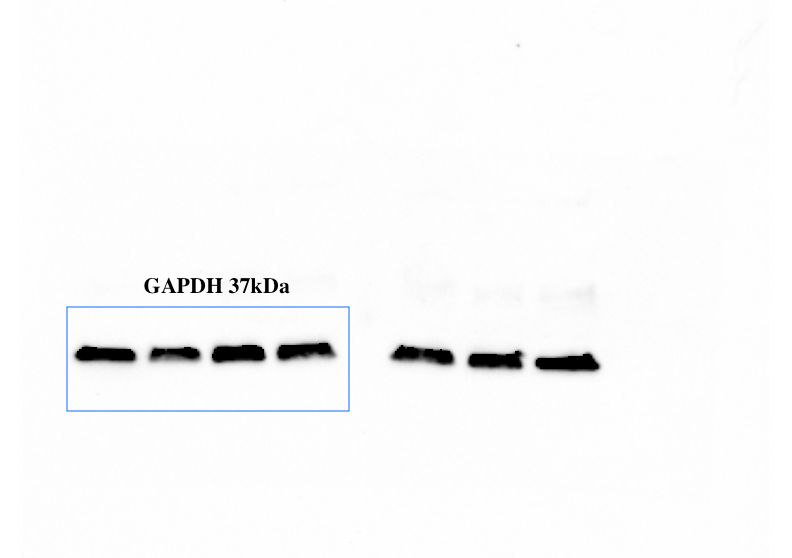

Supplement: Supplementary file 7 — Source Data Fig. 6 [file 44321_2024_35_MOESM7_ESM.zip › Figure 6/6D/western GAPDH left.tif]

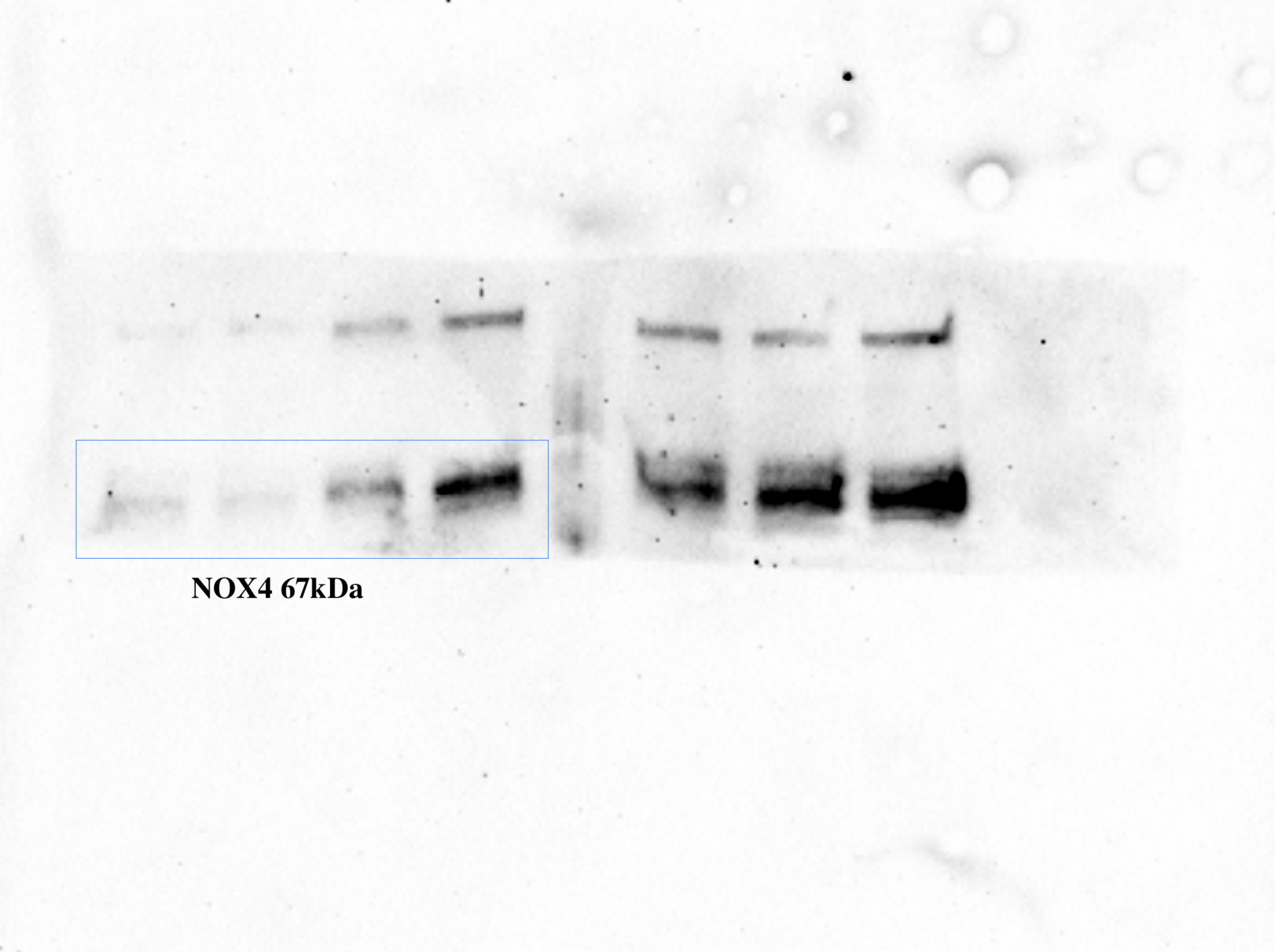

Supplement: Supplementary file 7 — Source Data Fig. 6 [file 44321_2024_35_MOESM7_ESM.zip › Figure 6/6D/western NOX4 left.tif]

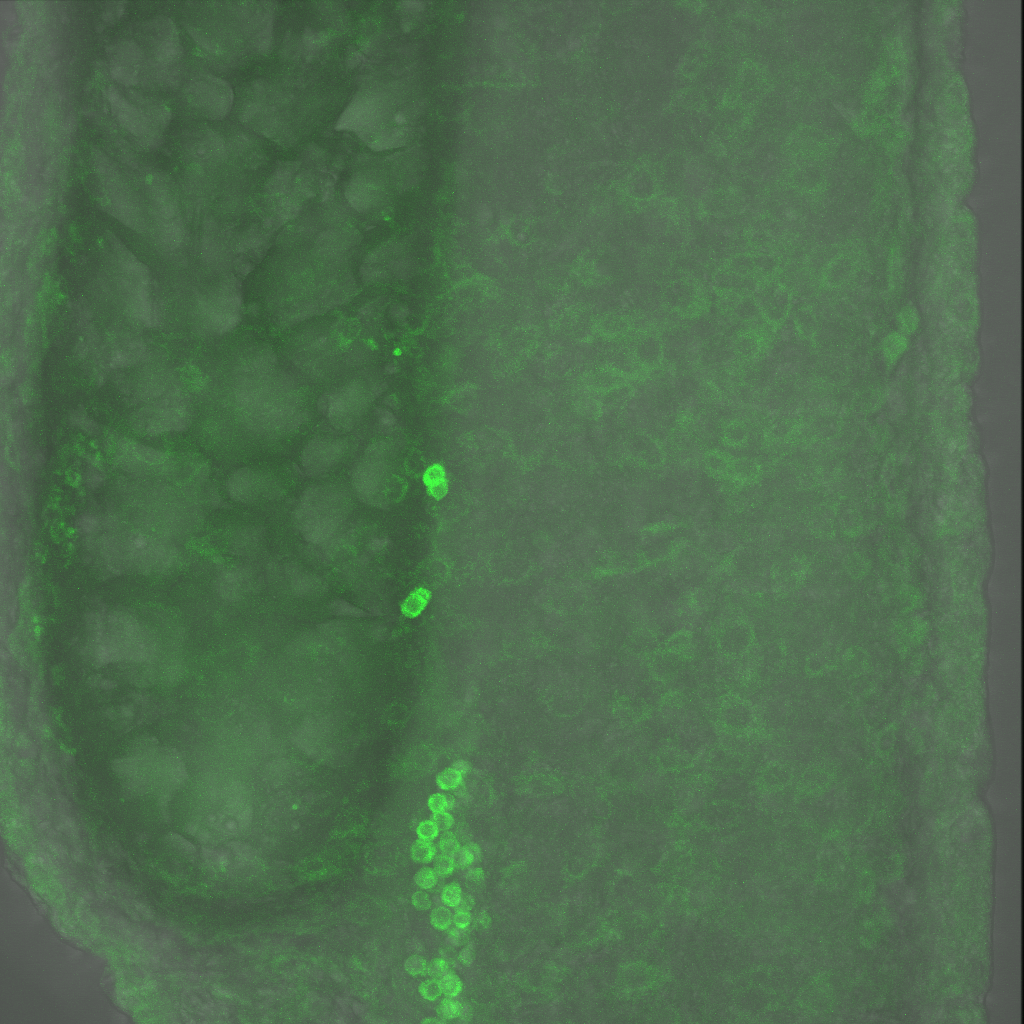

Supplement: Supplementary file 8 — Source Data Fig. 7 [file 44321_2024_35_MOESM8_ESM.zip › Figure 7/7D/Merge NOX4Y512C.tif]

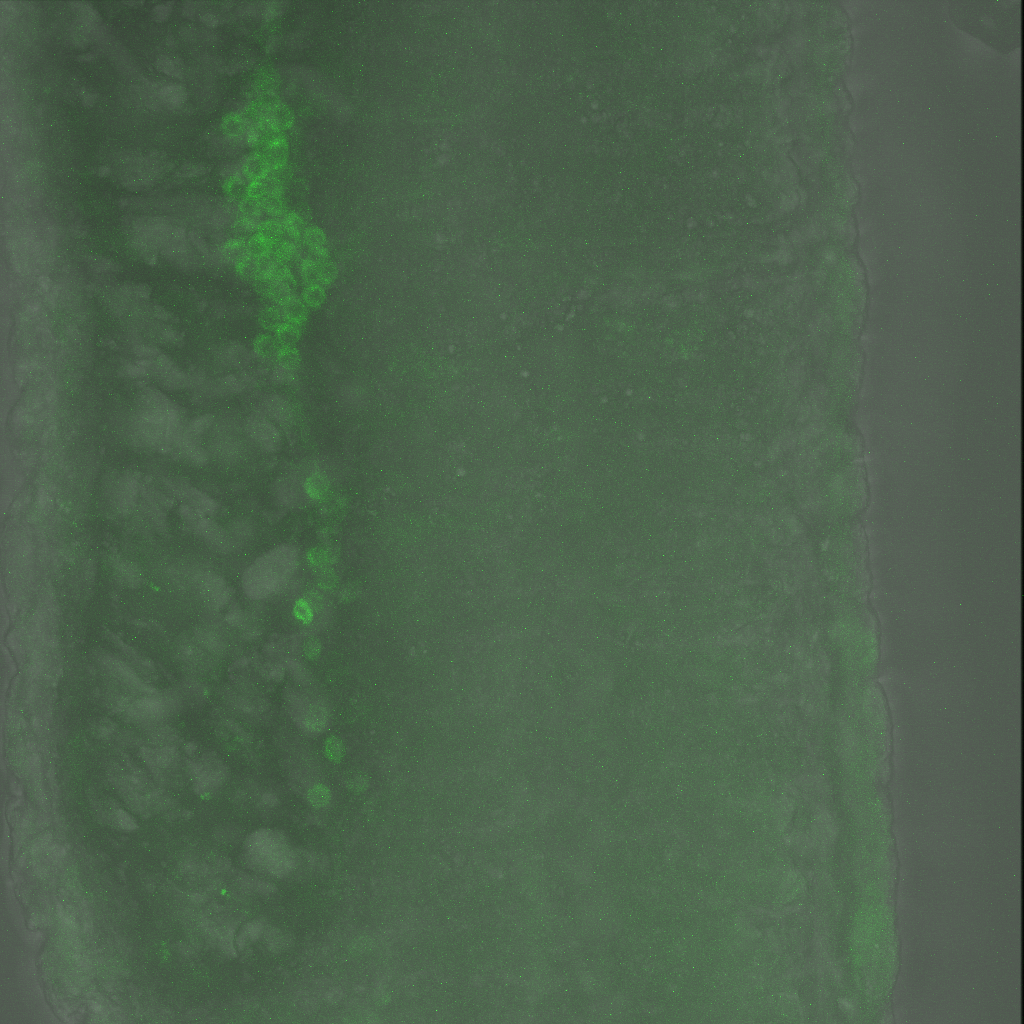

Supplement: Supplementary file 8 — Source Data Fig. 7 [file 44321_2024_35_MOESM8_ESM.zip › Figure 7/7D/Merge NOX4Y512fsX20.tif]

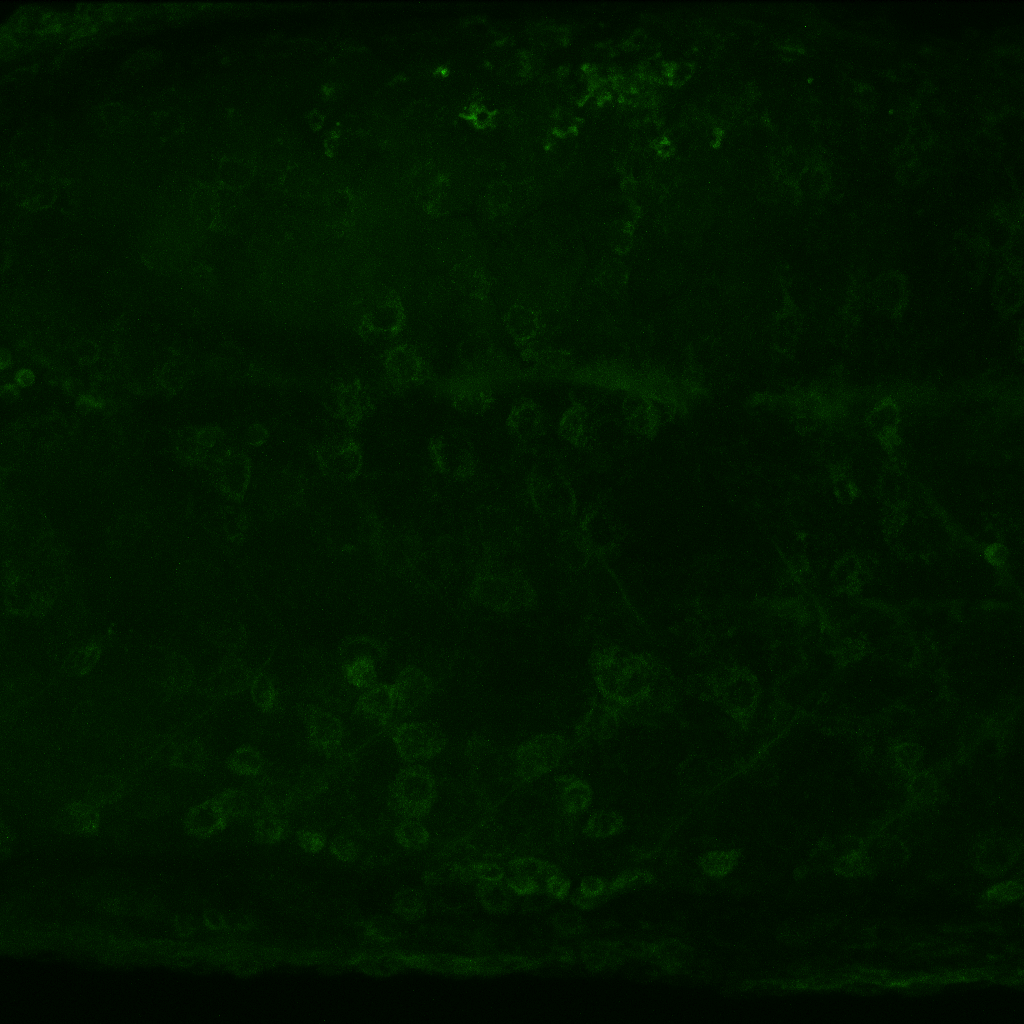

Supplement: Supplementary file 8 — Source Data Fig. 7 [file 44321_2024_35_MOESM8_ESM.zip › Figure 7/7D/DCFH-DA NOX4V369F.tif]

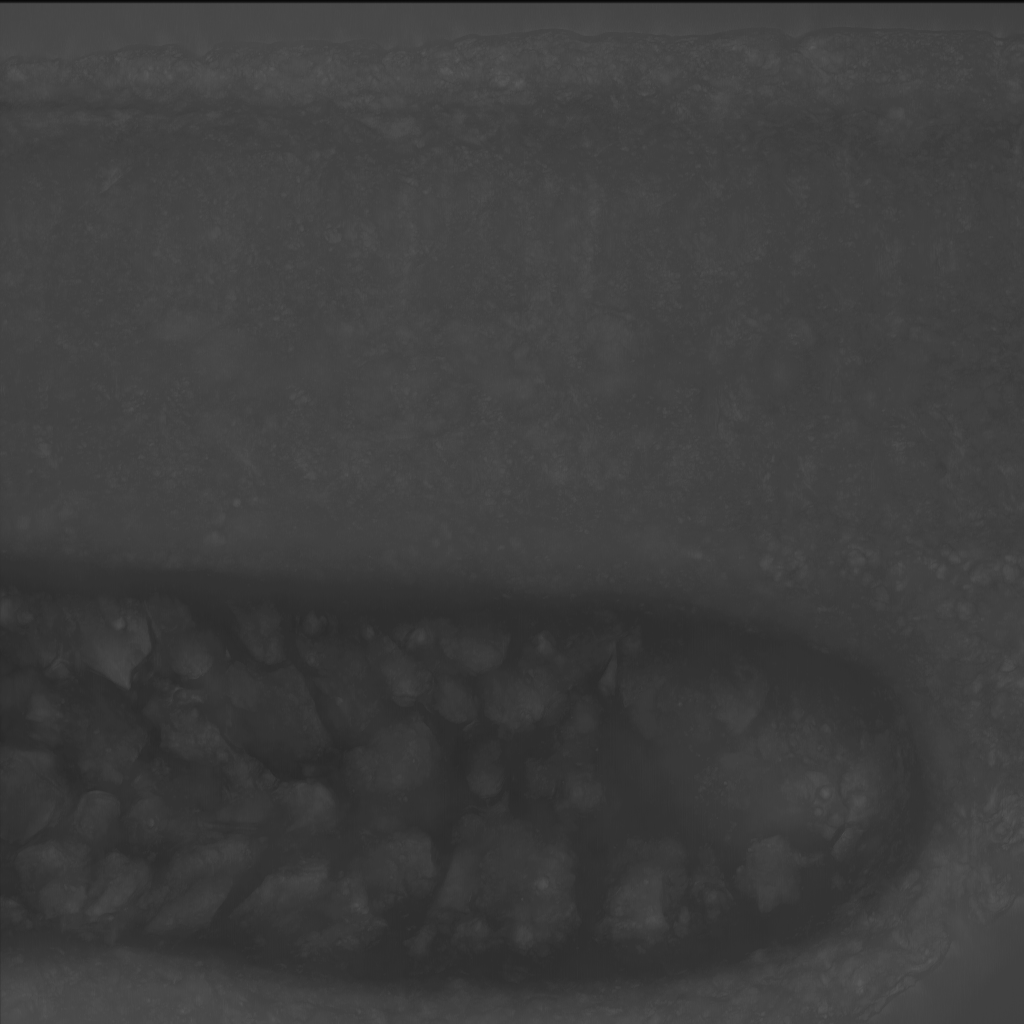

Supplement: Supplementary file 8 — Source Data Fig. 7 [file 44321_2024_35_MOESM8_ESM.zip › Figure 7/7D/bright field NOX4Y512C.tif]

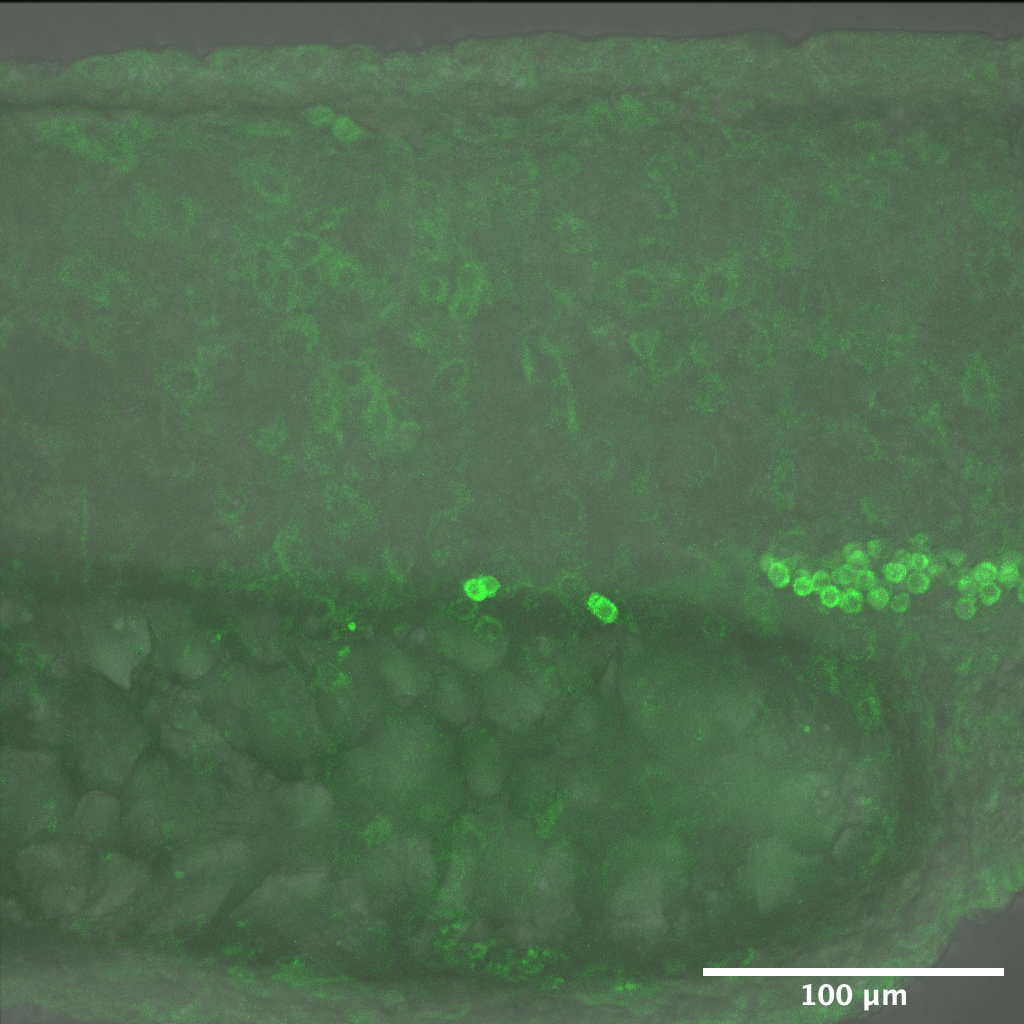

Supplement: Supplementary file 8 — Source Data Fig. 7 [file 44321_2024_35_MOESM8_ESM.zip › Figure 7/7D/Merge NOX4Y512C with bar.tif]

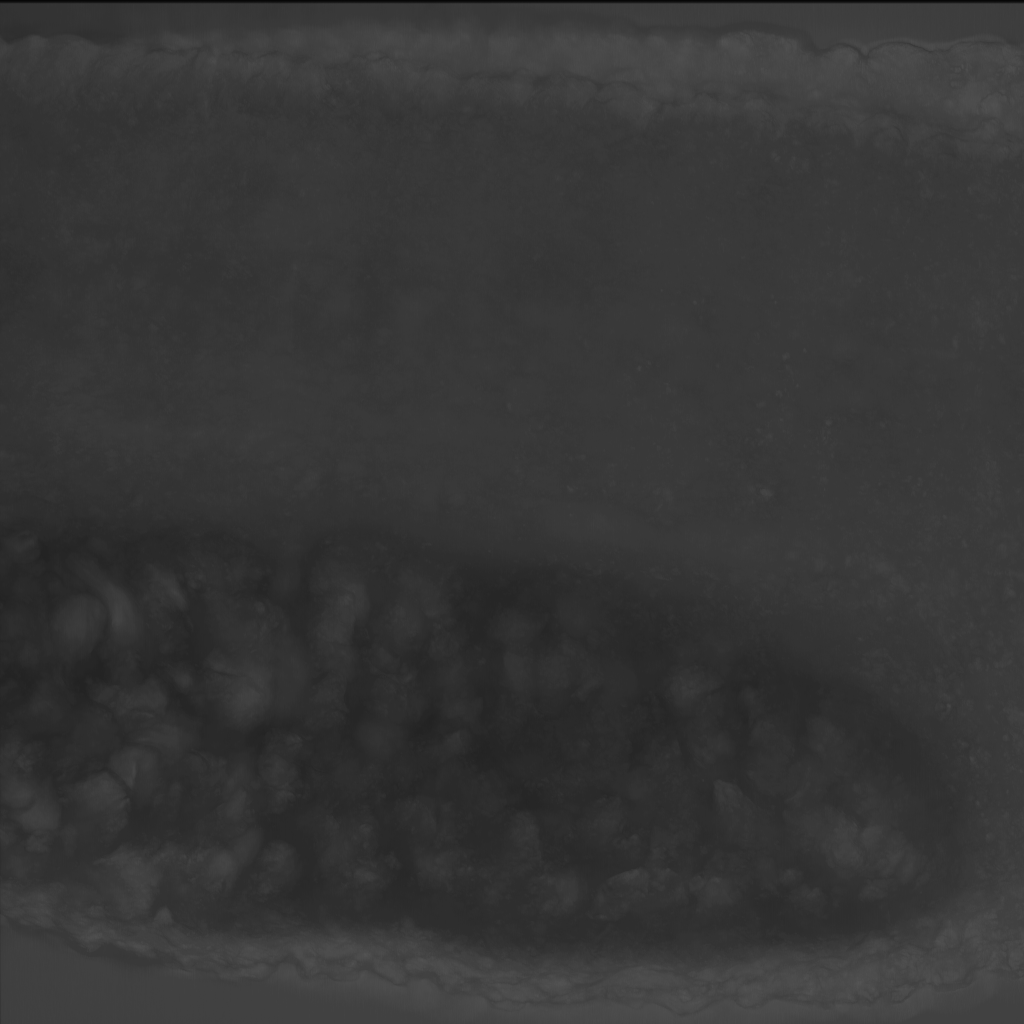

Supplement: Supplementary file 8 — Source Data Fig. 7 [file 44321_2024_35_MOESM8_ESM.zip › Figure 7/7D/bright field NOX4WT.tif]

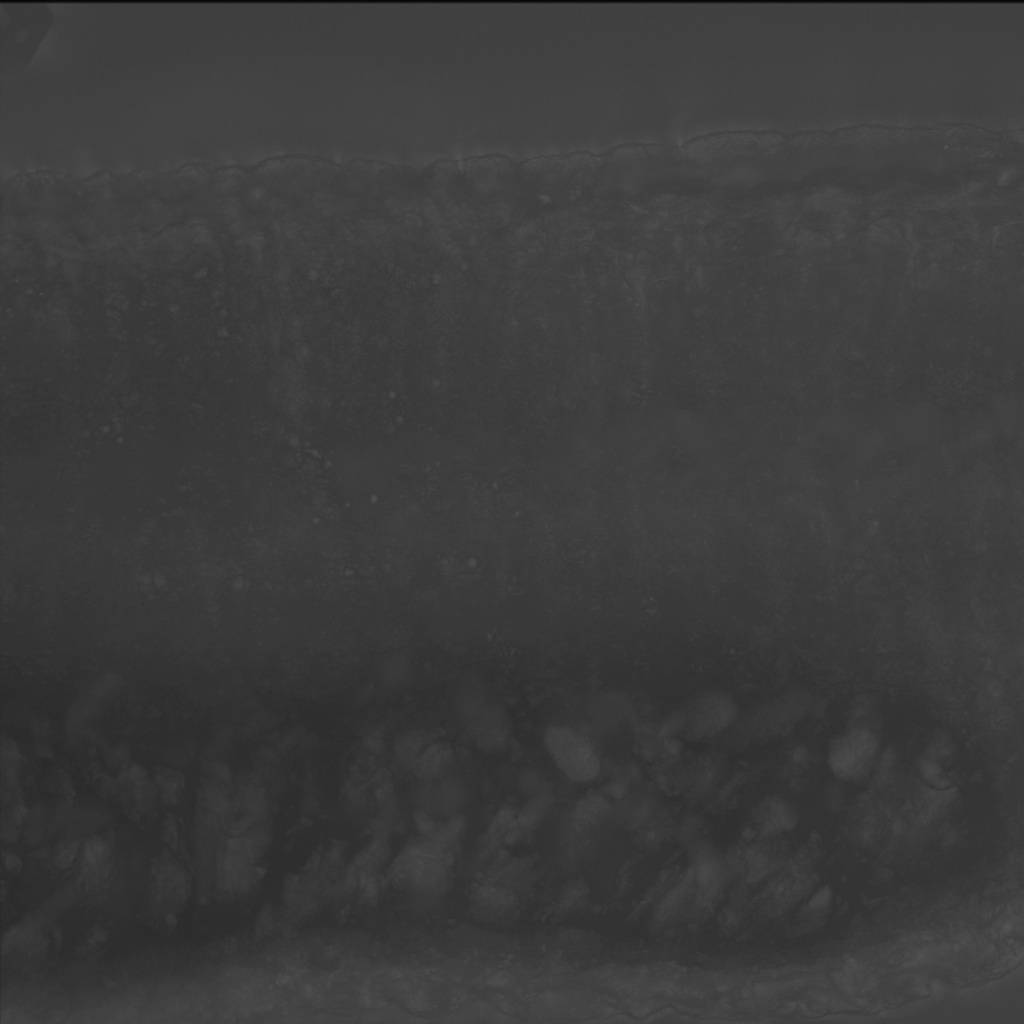

Supplement: Supplementary file 8 — Source Data Fig. 7 [file 44321_2024_35_MOESM8_ESM.zip › Figure 7/7D/bright field NOX4Y512fsX20.tif]

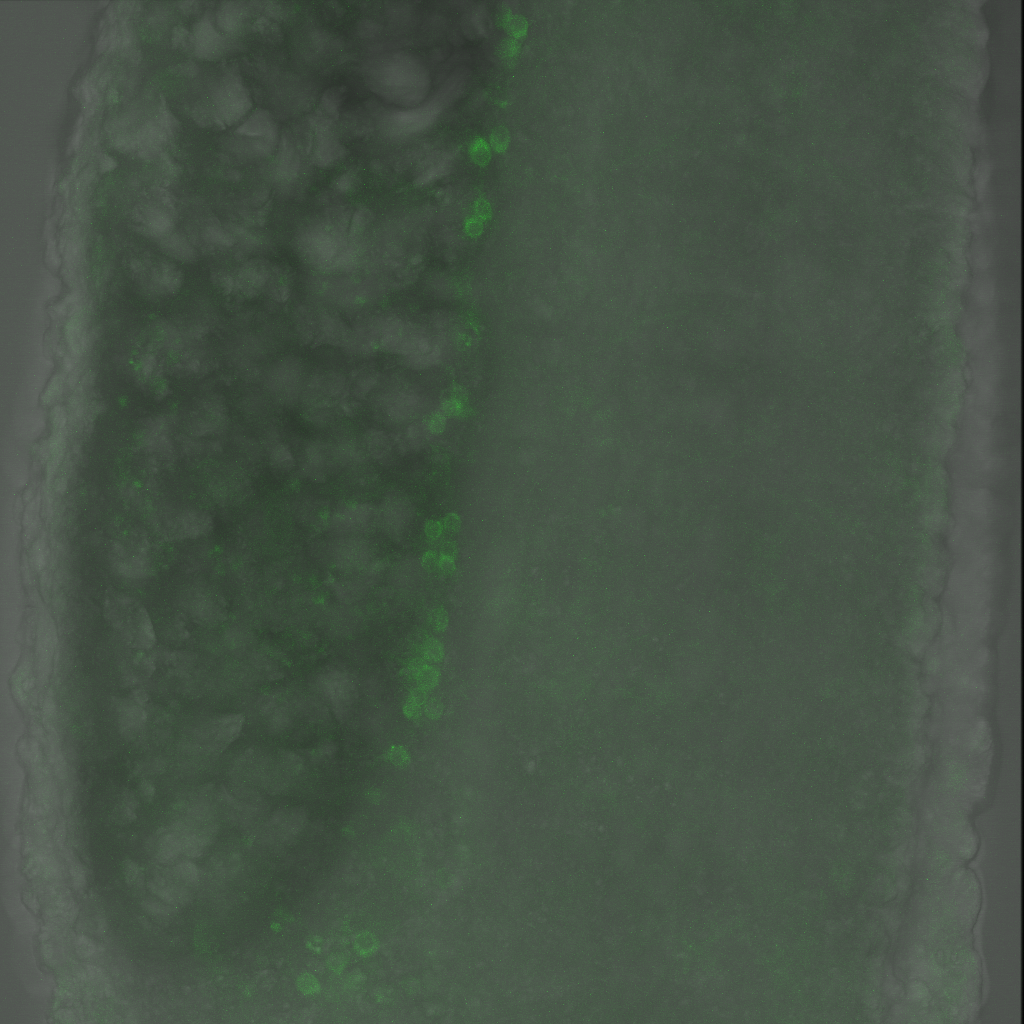

Supplement: Supplementary file 8 — Source Data Fig. 7 [file 44321_2024_35_MOESM8_ESM.zip › Figure 7/7D/Merge NOX4WT.tif]

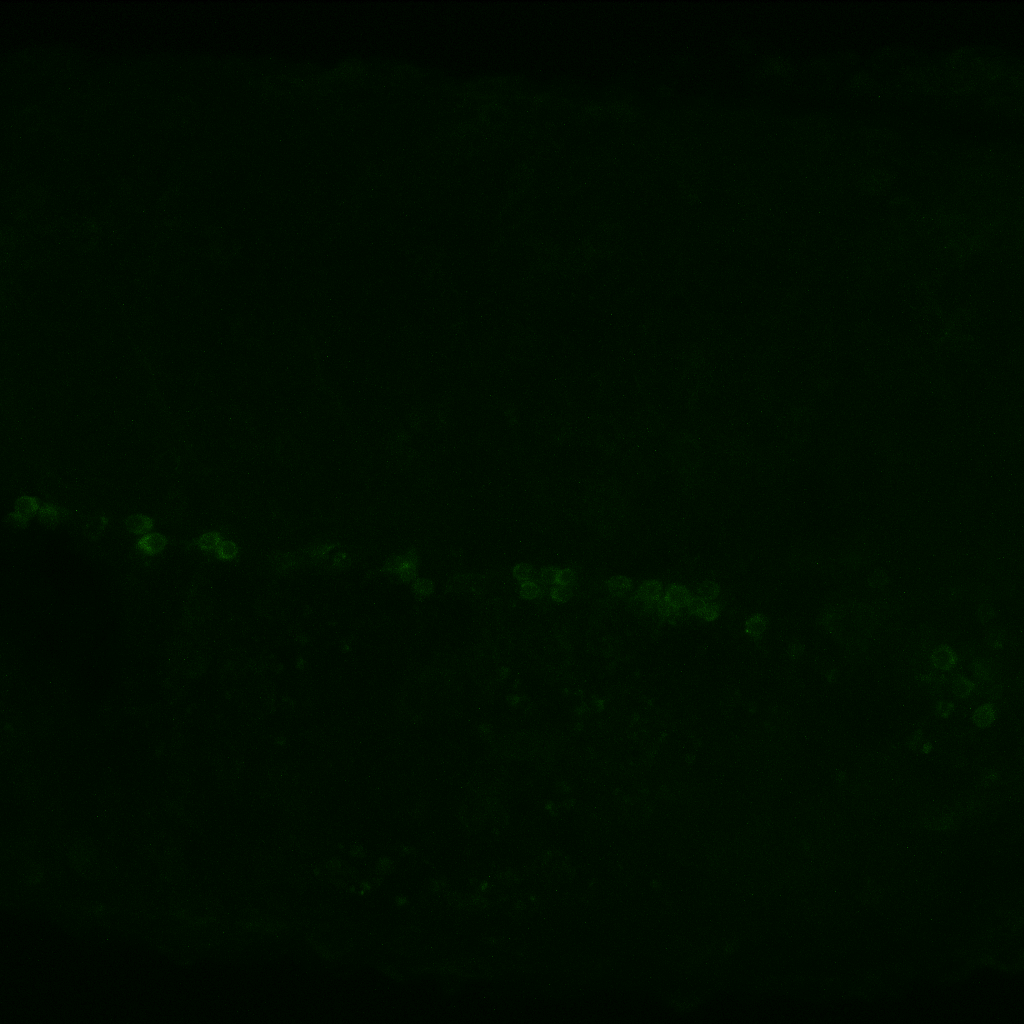

Supplement: Supplementary file 8 — Source Data Fig. 7 [file 44321_2024_35_MOESM8_ESM.zip › Figure 7/7D/DCFH-DA NOX4WT.tif]

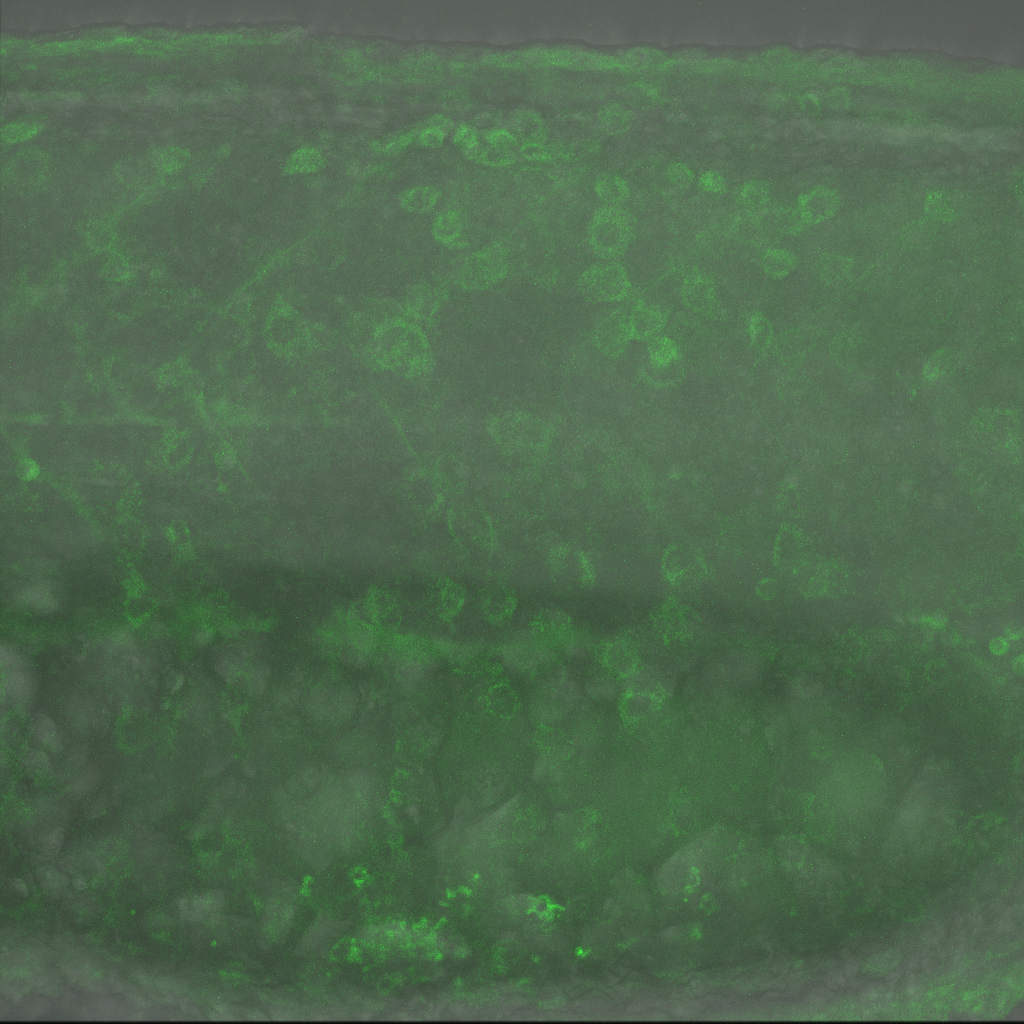

Supplement: Supplementary file 8 — Source Data Fig. 7 [file 44321_2024_35_MOESM8_ESM.zip › Figure 7/7D/Merge NOX4V369F.tif]

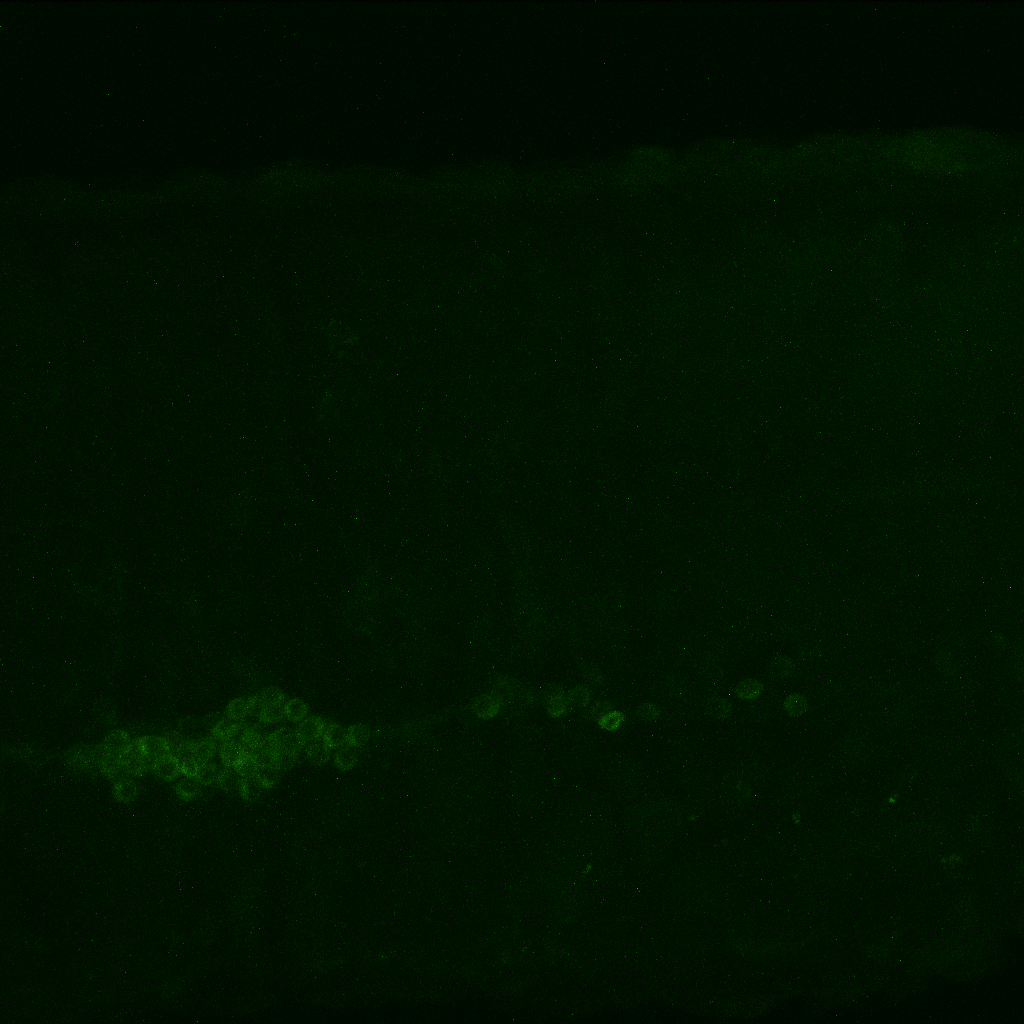

Supplement: Supplementary file 8 — Source Data Fig. 7 [file 44321_2024_35_MOESM8_ESM.zip › Figure 7/7D/DCFH-DA NOX4Y512fsX20.tif]

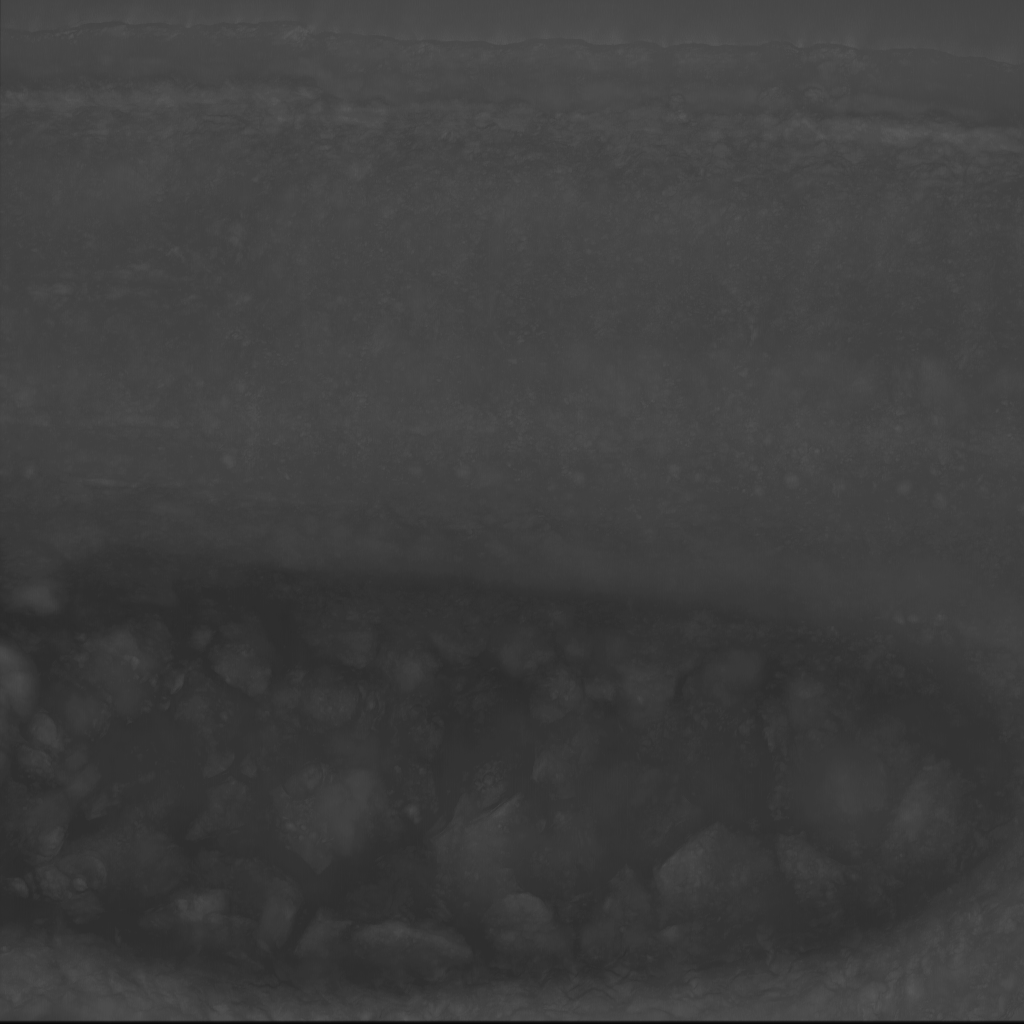

Supplement: Supplementary file 8 — Source Data Fig. 7 [file 44321_2024_35_MOESM8_ESM.zip › Figure 7/7D/bright field NOX4V369F.tif]

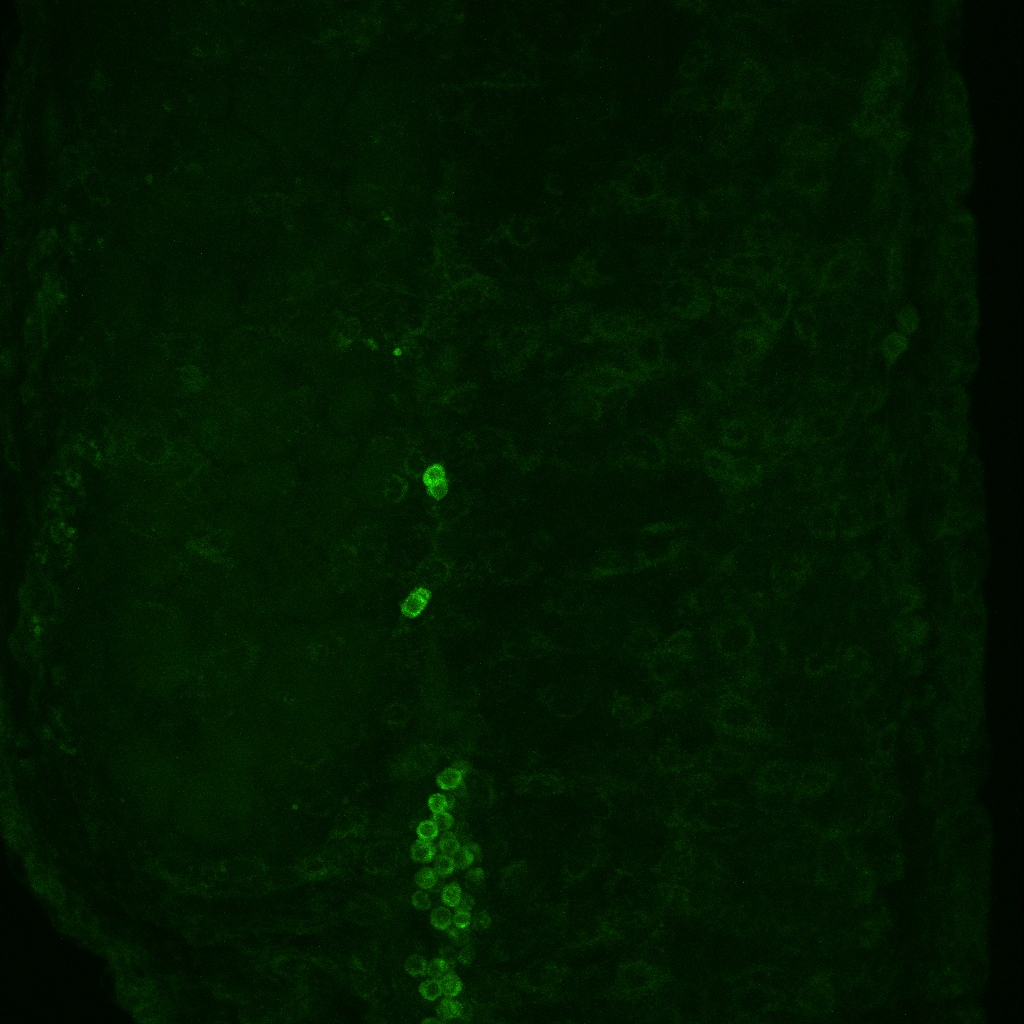

Supplement: Supplementary file 8 — Source Data Fig. 7 [file 44321_2024_35_MOESM8_ESM.zip › Figure 7/7D/DCFH-DA NOX4Y512C.tif]

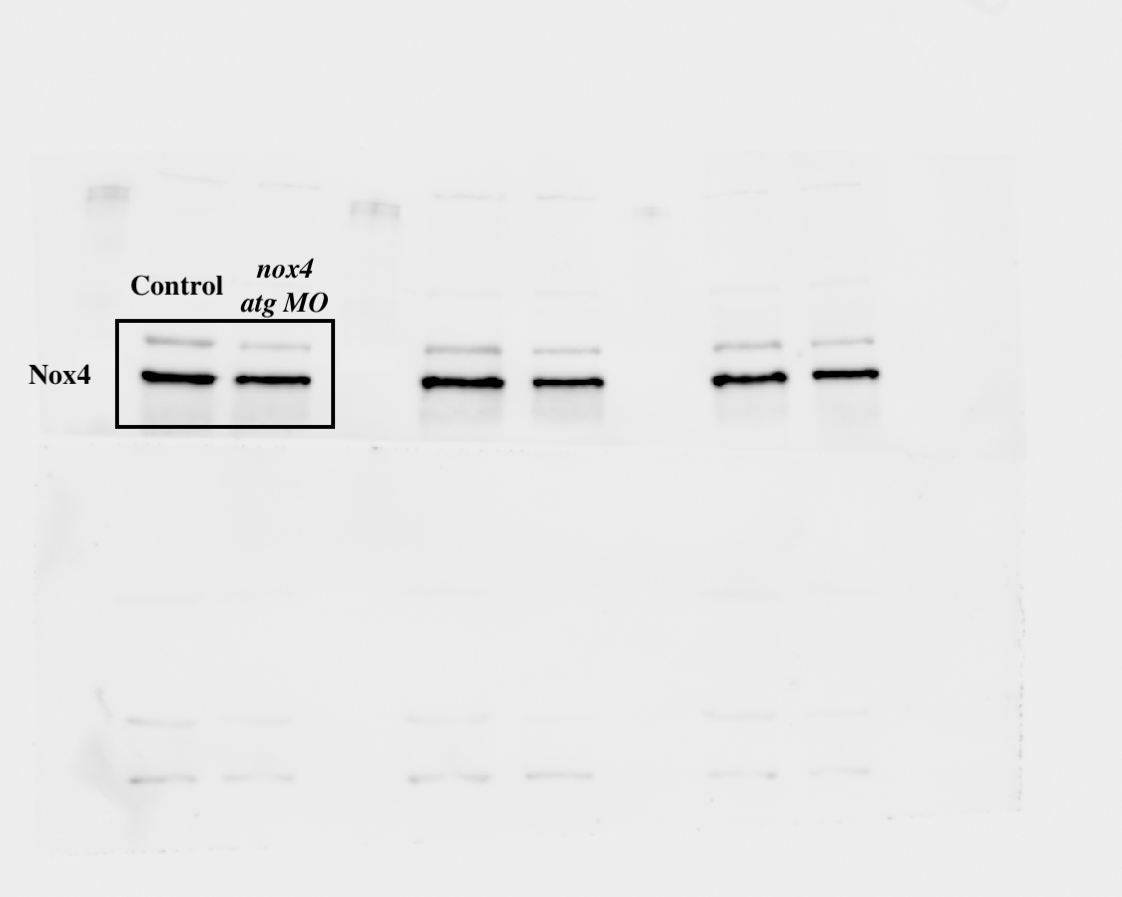

Supplement: Supplementary file 8 — Source Data Fig. 7 [file 44321_2024_35_MOESM8_ESM.zip › Figure 7/7A/western Nox4.tif]

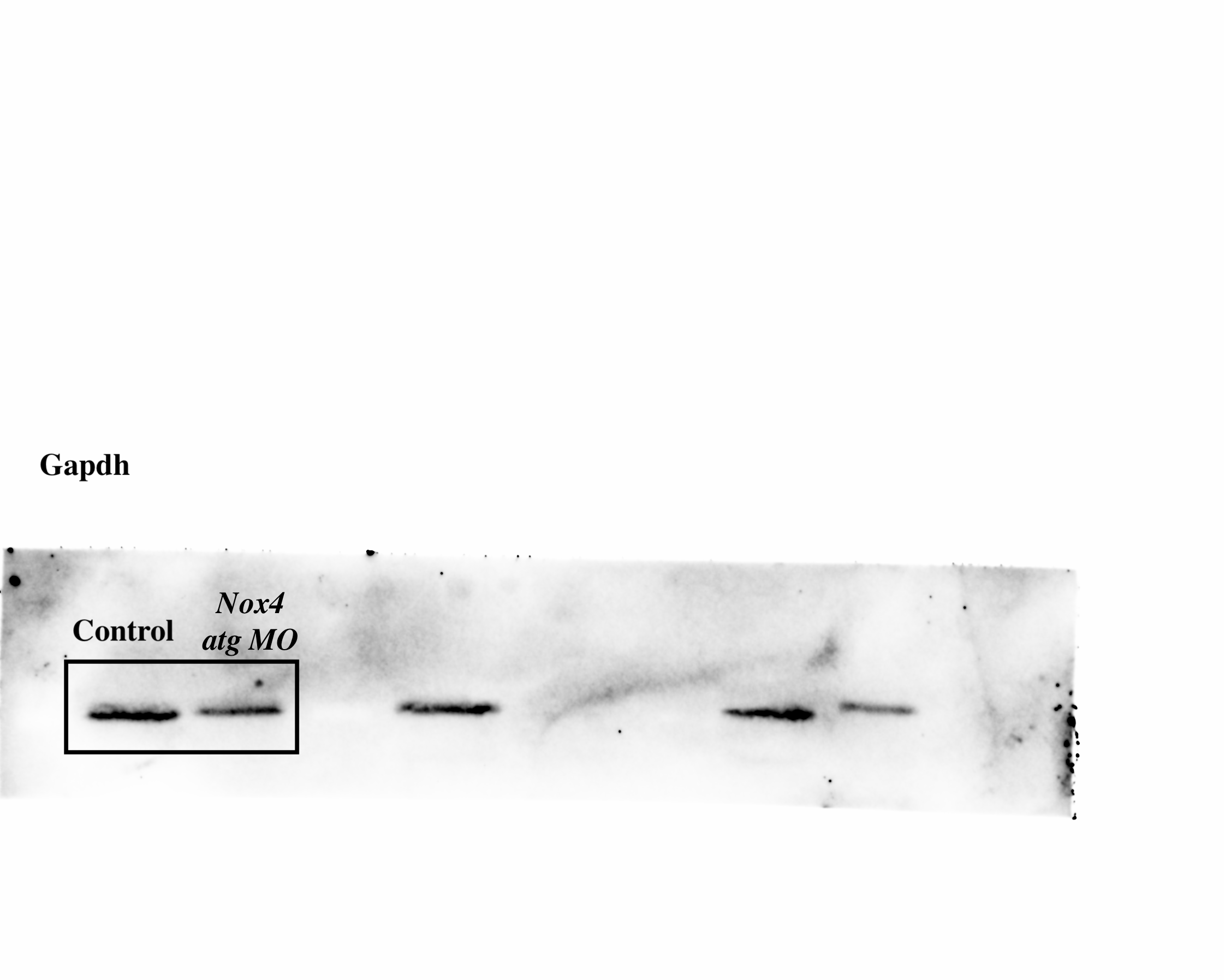

Supplement: Supplementary file 8 — Source Data Fig. 7 [file 44321_2024_35_MOESM8_ESM.zip › Figure 7/7A/western Gapdh.tif]
