# Supplementary material for: Rare coding variants in NOX4 link high ROS levels to psoriatic arthritis mutilans
Source: EMBO Mol Med. 2024 Feb 20;16(3):9. doi: 10.1038/s44321-024-00035-z (PMC10940640; doi:10.1038/s44321-024-00035-z)
Supplement: Supplementary file 11 — Expanded View Figures [file 44321_2024_35_MOESM11_ESM.pdf]

# Expanded View Figures

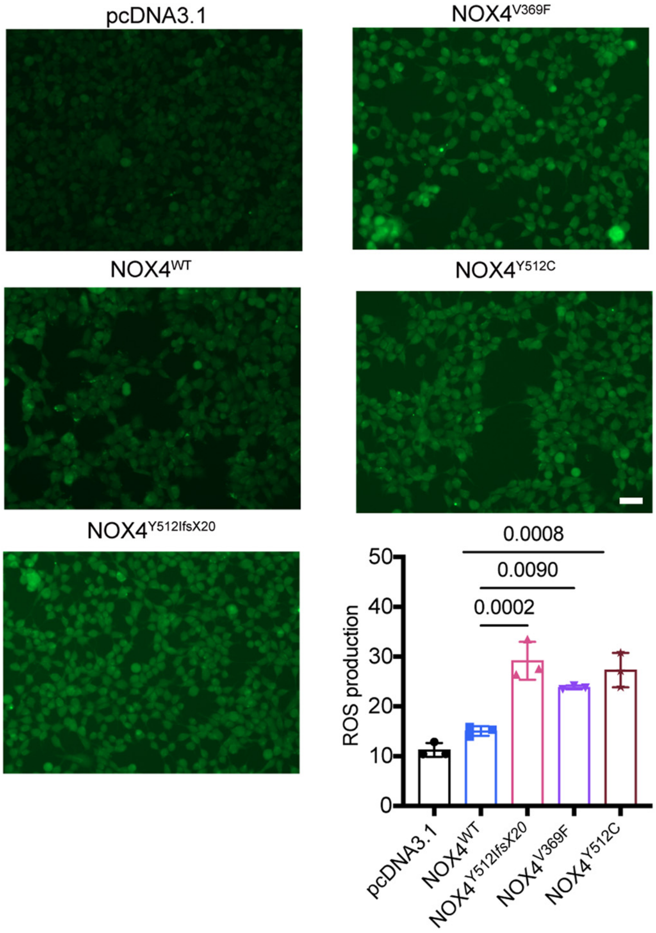


**Figure EV1. Elevated ROS levels in HEK293 stable transfected cell lines expressing *NOX4* variants.**

HEK293 cells were subjected to 12 h of serum starvation after stably transfection with following plasmids: pcDNA3.1, *NOX4$^{wt}$*, *NOX4$^{Y512IfsX20}$*, *NOX4$^{Y512C}$*, and *NOX4$^{V369F}$*. Fluorescence imaging was conducted following the incubation with 10 μM DCFH-DA. Representative photomicrographs of the fluorescence are displayed and quantification of the mean fluorescence intensity was performed with ImageJ software. $N = 3$. Scale bars: 100 μm. Data information: DCFH-DA 2′,7′-dichlorofluorescein diacetate. Data presented as mean ± SD. $N =$ biological replicates. The $P$ value was calculated by the ordinary one-way ANOVA multiple comparisons with Turkey correction of multiple hypothesis tests. Source data are available online for this figure.

 © The Author(s)

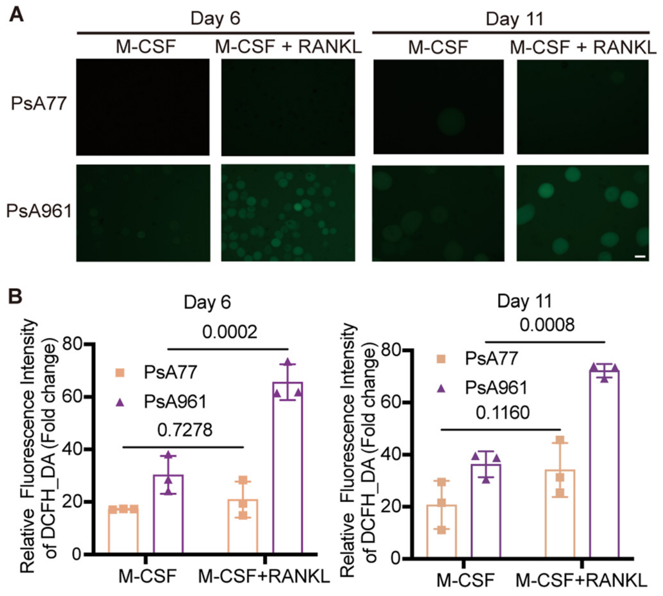


**Figure EV2. Impact of ROS on osteoclasts differentiation from PsA961 (*NOX4*$^{Y512C}$).**

(**A**) Cells were cultured in the presence of M-CSF or M-CSF and RANKL. Visualization of ROS detected by DCFH-DA on Day 6 and Day 11, indicating a higher ROS effect in PsA961-differentiated osteoclasts. Scale bar: 100 μm. (**B**) The relative fluorescence intensity of ROS was quantified by GraphPad Prism9.0.0. $N = 3$. Data information: DCFH-DA 2′,7′-dichlorofluorescein diacetate. $N =$ biological replicates. For graph (**B**), error bars in figure represent mean ± SD (two-way ANOVA with Tukey's multiple comparisons tests). Source data are available online for this figure.

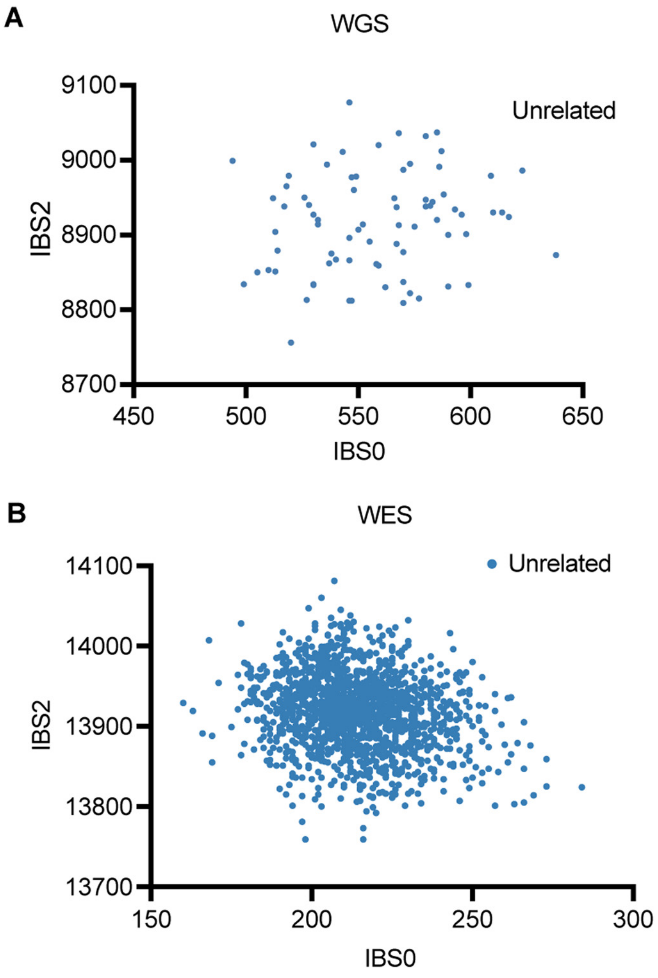


**Figure EV3. Relatedness plot for WGS (A) and WES (B) samples.**

(**A**) Relatedness plot for WGS samples. Six samples from the SweGene database (https://swefreq.nbis.se/) were added to the WGS analysis. Each dot represents a pair of samples. (**B**) Relatedness analysis of all WES samples. Data information: IBS0 is the number of sites where 1 sample is homozygous for the reference allele and the other is homozygous for the alternate allele. IBS2, is the count of sites where a pair of samples were both homozygous or both heterozygous. WGS Whole-Genome Sequencing, WES Whole-Exome Sequencing. Source data are available online for this figure.

 © The Author(s)
